# Niche‐neutral theoretic approach to mechanisms underlying the biodiversity and biogeography of human microbiomes

**DOI:** 10.1111/eva.13116

**Published:** 2020-10-13

**Authors:** Zhanshan (Sam) Ma

**Affiliations:** ^1^ Computational Biology and Medical Ecology Lab State Key Laboratory of Genetic Resources and Evolution Kunming Institute of Zoology Chinese Academy of Sciences Kunming China; ^2^ Center for Excellence in Animal Evolution and Genetics Chinese Academy of Sciences Kunming China

**Keywords:** Hierarchical Dirichlet process, human microbiome biogeography, multisite neutral (MSN) model, niche‐neutral hybrid model, neutral drifts, niche differentiations, niche‐neutral continuum

## Abstract

The human microbiome consists of five major regional biomes distributed in or on our five body sites including skin, oral, lung, gut, and reproductive tract. Its biogeography (the spatial and temporal distribution of its biodiversity) has far‐reaching implications to our health and diseases. Nevertheless, we currently have very limited understanding on the *mechanisms* shaping the biogeography, since it is often rather difficult to determine the relative importance of drift, dispersal, speciation, and selection, the four processes (mechanisms) determining the patterns of microbial biogeography and community dynamics according to a recent synthesis in community ecology and biogeography. To disentangle these mechanisms, I utilize multisite neutral (MSN) model and niche‐neutral hybrid (NNH) model to analyze large number of truly multisite microbiome samples covering all five major human microbiome habitats, including 699 metacommunities and 5,420 local communities. Approximately 89% of metacommunities and 92% local communities exhibit patterns indistinguishable from neutral, and 20% indistinguishable from niche‐neutral hybrid model, indicating the relative significance of stochastic neutral forces versus deterministic niche selection in shaping the biogeography of human microbiome. These findings cast supporting evidence to van der Gast's revision to classic Bass‐Becking doctrine of microbial biogeography: “*Some things are everywhere and some things are not. Sometimes the environment selects and sometimes it doesn't,*” offering the first educated guess for “*some*” and “*sometimes*” in the revised doctrine. Furthermore, the logistic/Cox regression models describing the relationships among community neutrality, niche differentiation, and key community/species characteristics (including community diversity, community/species dominance, speciation, and migration rates) were constructed to quantitatively describe the niche‐neutral continuum and the influences of community/species properties on the continuum.

## 1 INTRODUCTION

The importance of microbes is self‐evident for just one thing: Without microbes, the earth will be piled up with bodies and remains of plants and animals, and both its biogeochemical cycling and organic matter cycling will be broken (Grossart et al., 2010; Wilson, 1994). Human microbiome refers to microbes in the form of microbial biomes distributed on or in our bodies and consists of the five major regional biomes occupying five body sites including gut, oral, skin, lung, and reproductive tract, but its distribution is not limited to the five major habitats. In fact, human microbiome has also been found in body fluids such as tear, semen, breastmilk, some tissues, and even blood (Paisse et al., 2016). In analogy, the distribution of human microbiome has little difference from the various biomes of plants and animals distributed on the earth planet except for the following two differences: (a) The host of human microbiome or our body is a live organism, while the earth planet is largely inorganic; microbiome–human body is predominantly a symbiotic ecosystem. (b) While there is only one earth to host lives, there are approximately 7 billions of humans to host the human microbiomes. The first difference means that our interactions with the microbiome are not only far closer and direct but also more important than our interactions with other biomes or the environment. Indeed, the US‐NIH HMP (human microbiome project) and EU Meta‐HIT (metagenomics of the human intestinal tract) launched around 2008 jump‐started our deep understanding of the human microbiome (HMP Consortium, 2012; Lozupone et al., 2012; Turnbaugh et al., 2007; http://metahit.eu/). The extensive studies during the last decade have revealed far‐reaching influences of the human microbiome on our health and disease. While traditional clinic medicine and ecology appear to be two rather distant fields, human microbiome research is largely an ecology problem. There is an urgent need for emerging *medical ecology*, which lies in the interdisciplinary intersections between ecology, medical microbiology, bioinformatics, and clinical medicine (Ma, 2017, 2019, 2020; Ma et al., 2016). Therefore, ecology, especially theoretical ecology, has been playing a critical role in microbiome research. The second difference (*i.e*., one earth for earth biomes vs. seven billion humans for human microbiomes) implies that human microbiome research offers us unprecedented opportunities to test existing ecological theories and to develop an inclusive ecology across scales from molecules to ecosystems.

Understanding the mechanisms governing microbial community dynamics can provide critical insights into understanding the forces shaping microbial biogeography, which can be defined as the spatial and temporal distribution of microbial biodiversity (*see* excellent reviews by Martiny et al., 2006, Hanson et al., 2012, van der Gast, 2015). For decades, niche theory, neutral theory, and their hybrid integrations have been the premier ecological theories for investigating the mechanisms underlying community assembly and dynamics in macro‐ecology (Fisher & Mehta, 2014; Haegeman & Etienne, 2017; Hubbell, 2001; Jeraldo et al., 2012; Kalyuzhny et al., 2014; Matthews & Whittaker, 2014; McGill, 2003; McGill et al., 2006; Noble & Fagan, 2015; Ofiteru et al., 2010; Pigolotti & Cencini, 2013; Rosindell et al., 2011, 2012; Stokes & Archer, 2010; Tang & Zhou, 2013; Tilman, 2004). Compared with the studies of neutral theory in macrobial ecology, there have been relatively few applications of neutral theory in microbial communities (*e.g*., Curtis & Sloan, 2004, Sloan et al., 2006; Sloan et al., 2007, Woodcock et al., 2007, Zhang et al., 2010, Costello et al., 2012, Venkataraman et al., 2015, Li & Ma, 2016, Chen et al., 2017, Dai et al., 2017, Ma, 2020).

**FIGURE 1:**
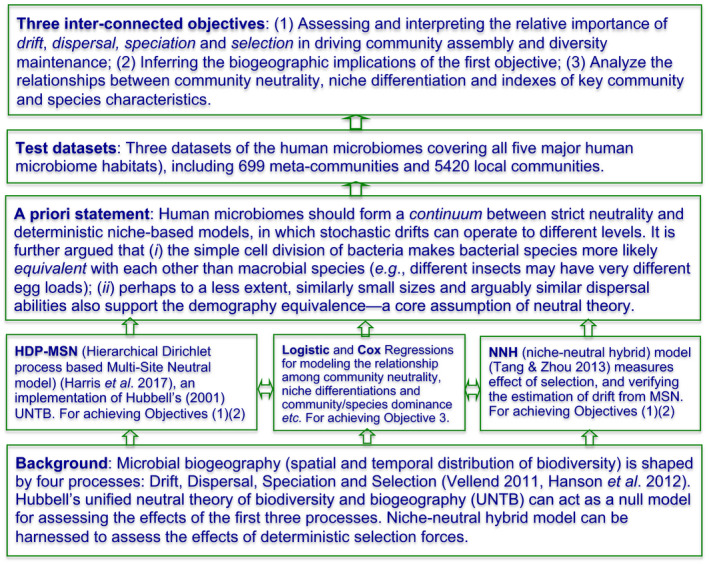
A diagram illustrating the study design and the relationships among its various aspects including theoretic background and models, priori, test datasets and the research objectives

I subscribe to the Vellend (2010) identification of the four key processes (mechanisms) that shape the community structure and dynamics, including *drift*, *dispersal*, *speciation,* and *selection*. Hanson et al. (2012) proposed that *drift*, *dispersal*, *mutation*, and *selection* govern the formation and maintenance of the microbial biogeographic patterns on ecological and evolutionary scales that are hardly separable. Hubbell’s (2001) unified neutral theory of biodiversity and biogeography (UNTB) was built on the combination of the former three in the Vellend (2010) and Hanson et al. (2012) lists, excluding selection (Rosindell et al., 2011, 2012). The first objective (Figure 1), also the primary objective, of this article was to evaluate the importance of *dispersal*, *drift, speciation,* and *selection* in driving the community assembly and diversity maintenance, in which lots of advances have been made in the last decade, but significant issues are still unsettled (Adams et al., 2013; Clark, 2009; Foissner, 2006; Gilbert & Levine, 2017; Grossart et al., 2010; He et al., 2012; Hu et al., 2006; Liu & Zhou, 2011; Lowe & McPeek, 2014; May et al., 2011; Svensson et al., 2018). For example, it is still controversial whether the neutral and niche theories can produce similar patterns *via* different mechanisms; alternatively, both the theories may interact to jointly produce the observed diversity patterns (Tang & Zhou, 2013). We realized the extensive challenges in resolving these and similar issues, and resorted to two recent additions of hybrid and neutral models (Harris et al. 2017, Tang & Zhou, 2013) supported with extensive truly multisite HMP datasets to get the best‐educated “guess” for the proposed objective.

The multisite neutral (MSN) model proposed by Harris et al. (2017) was used for implementing the UNTB requiring truly multisite samples; that is, the required samples are taken from spatially connected local communities (sites) on ecological timescales. Theoretically, Harris et al. (2017) represent a major advance in dealing with intractable computational algorithm for the parameter estimations of the multisite UNTB model with different immigration rates. The advance allows for more accurate parameter estimation and reliable testing of neutral theory. Obviously, its advantage can only be leveraged with truly multisite datasets. A minor issue in Harris et al. (2017) was that the gut microbiome datasets utilized to demonstrate their MSN model are not truly multisite samples. Instead, the gut microbiome samples they used were taken from independent individuals, and at ecological timescales, the occurrence of microbial migrations among those individuals may be unrealistic, even though the gene exchanges at evolutionary timescales are possible. Hence, this study should represent the first rigorous application of the Harris et al. (2017) MSN model, fitted to extensive truly multisite human microbiome datasets (including 699 metacommunities and 5,420 local communities).

Natural communities are typically structured by stabilizing niche differences and competitive asymmetries among species, which are expected to modulate the effects of drift and generate communities that are distinctly non‐neutral (Gilbert & Levine, 2017). Consequently, the level of neutrality exhibited by a community can act as a measure of *drift effect* in the community, and we take this advantage of the UNTB in this study. Therefore, my estimation of the drift effect with neutral theory is expected to be conservative (reliable). Furthermore, it is virtually a consensus that there is a *continuum* between strict neutrality and deterministic niche‐based models, in which ecological drift can operate at different levels (Gotelli & McGill, 2006; He et al., 2012; Hu et al., 2006; Hubbell, 2001, 2005; Svensson et al., 2018; Vellend, 2010). With this consideration, the Tang and Zhou (2013) niche‐neutral hybrid model offers an ideal augment to the UNTB in determining the relative importance or balance of stochastic neutral drift and dispersal versus deterministic selection in the neutral‐niche continuum. Moreover, I argue that, in microbial communities, the stochastic neutral drifts (including stochasticities in demography and dispersal) should certainly exist, at least, for the following microbial characteristics: The simple cell division of bacterial reproduction makes bacterial species more likely *equivalent* with each other than macrobial species (*e.g*., different insect species may have very different egg loads); and perhaps to a less extent, similarly small sizes and arguably similar dispersal abilities also support the assumption of species equivalence—the core of neutral theory.

The second objective of this study was to look into the implications of the first objective (*i.e*., neutrality vs. niche balance) to microbial biogeography (Figure 1). A central theme still hotly debated in microbial biogeography research is whether microbes are cosmopolitanism or endemism. The traditional view in microbiology, first proposed by Beijerinck (1913) (cited in Foissner, 2006) and further refined by Baas‐Becking (1934), that “*everything is everywhere, but the environment selects who stays locally*” assumes high dispersal rates of microorganisms, leading to their ubiquity. The endemism (biogeographically restricted natives) is essentially about *selection*. Therefore, if microbial biogeography is totally determined by selection, then *dispersal* and *drift* are unlikely to be key processes in microbial communities. The present study with the neutral theory tool will help us evaluate the importance of dispersal and drift in driving microbial community assembly and dynamics. Furthermore, I also harness the power of niche‐neutral hybrid analysis to determine the importance of local selection *via* niche differentiations. This study is expected to offer the first educated guess for “*some things*” and “*sometimes*” in van der Gast (2015) recent revision to classic Bass‐Becking doctrine of microbial biogeography: “*Some things are everywhere and some things are not. Sometimes the environment selects and sometimes it doesn't*.”

The debate on the cosmopolitanism versus endemism also touches an even more fundamental issue in current growing acceptance and incorporation of traditional ecological principles and theories into microbial ecological research during the last decade (Carbonero et al., 2014; van der Gast, 2015). Much of the interests in translating principles and theories from macro‐ecology to microbial ecology has been focused on the question of microbial biogeography (van der Gast, 2015; Hanson et al., 2012; Martiny et al., 2006). Therefore, the issue surrounding the microbial biogeography becomes a test bed for developing an inclusive ecology. The core of the issue is whether or not the uniqueness of microbial biology and physiology is so strong that an inclusive ecology is valid. Testing the neutral theory and niche‐neutral balance, in particular the biogeography inferences from the test, should also be meaningful for developing the inclusive ecology theoretically. The third and less ambitious objective (Figure 1) of this article was to analyze the relationships between community neutrality, niche differentiation, and key community/species characteristic parameters including community diversity, community and species dominance, speciation, and migration rates.

## 2 MATERIALS AND METHODS

### 2.1 The study design and human microbiome datasets

Figure 1 illustrates the overall study design and the relationships among various aspects of the study including the three interconnected objectives, the integrated niche‐neutral theoretic approach [with the Harris et al. (2017) multisite neutral model and the Tang and Zhou (2013) niche‐neutral hybrid model], a conceptual priori based on the niche‐neutral continuum and four process (mechanism) synthesis of community dynamics and biogeography, and the human microbiome datasets used to implement the study design.

As shown in Table 1, I use three datasets of the human microbiome, including one from the HMP (human microbiome project). The datasets are essentially the *species abundance distribution* data in the form of marker gene abundance or 16S rRNA reads. A total of 699 metacommunities consisting of 5,420 local communities are included in the datasets. In this study, a metacommunity is referred to all microbiome samples (sites) belonging to a single individual subject, or to all samples belonging to one of the three major microbiome habitats (oral, skin, and vaginal) of the same individual subject; a local community is a "component" (corresponding to a sample or site) of metacommunity.

**TABLE 1:** The multisite human microbiome datasets utilized for testing the MSN (multisite neutral) and NNH (niche‐neutral hybrid) models

Datasets	*N* ^a^	*S* ^b^	Sample description	Source
HMP (oral)	146	9	A cohort of 242 healthy adults were sampled at 15 (male) or 18 (female) body sites up to three times, 5,177 samples were collected and sequenced, 3.5 Tera‐bases of metagenomic sequence reads were obtained, and 16S rRNA‐based OTU tables were computed.	The HMP Consortium (2012)
HMP (skin)	159	4		
HMP (vaginal)	72	3		
HMP (total)	172	18		
Gut	11	7	77 biopsy tissue samples taken from terminal ileum, ileocecal valve, ascending colon, transverse colon, descending colon, sigmoid colon, and rectum of 11 healthy adults and 16S rRNA‐based OTU tables were obtained from 454 pyrosequencing.	Zhang et al. (2014)
Lung	139	4	A longitudinal 16S rRNA survey of the lung microbiome collected from 139 subjects with COPD (chronic obstructive pulmonary disease) at four stages defined as stable state, exacerbation, 2 weeks post‐therapy, and 6‐week recovery.	Wang et al. (2016)
Total or range	699	3–18		

^a^ *N* = the number of individual subjects (metacommunities) sampled and DNA‐sequenced. ^a^

^b^ *S* = the number of microbiome sites (local communities) sampled from each individual subject. ^b^

### 2.2 Harris et al.'s (2017) multisite neutral model (MSN)

The UNTB conceptually distinguishes local community dynamics from metacommunity dynamics, but both are driven by similar neutral processes (Hubbell, 2001, 2006). Specifically, many local communities are connected to a single metacommunity with different immigration rates. Harris et al. (2017) developed an efficient Bayesian fitting framework by approximating the neutral models with the hierarchical Dirichlet process (HDP) (Teh et al., 2006). Harris et al.'s (2017) approximation captures the essential elements of the UNTB, that is, neutrality, finite populations, and multisite metacommunity setting. For this reason, I term the Harris et al. (2017) HDP‐neutral approximation framework as the multisite neutral (MSN) model (for a summary and complete information on the mathematical algorithms and computational procedures of the Harris et al. (2017) MSN model, please refer to their original publication; Harris et al. (2017)). With Harris et al.’s MSN model, it is possible to distinguish between the neutral local community (given a non‐neutral metacommunity) and the full UNTB (where the metacommunity also assembles neutrally). Hence, the neutrality tests are performed at both metacommunity level and local community level.

### 2.3 Tang & Zhou's (2013) niche‐neutral hybrid model (NNH)

Tang and Zhou (2013) proposed a hybrid niche‐neutral hybrid (NNH) model by revising the Volkov et al. (2007) neutral model for multiple discrete communities. Volkov et al. (2007) assumed the interspecies interactions in a steady‐state community may be ignored and all species in the community become functionally equivalent. A unique feature of Tang and Zhou (2013) niche‐neutral hybrid model is its incorporation of niche differentiations into the Volkov et al. (2007) multisite neutral model. Specifically, the per capita birth to death rates (*x*) and immigration parameter (γ) vary among species from different niches. In the case of the multisite microbiome datasets used in this study, I treat each site as a niche occupied by a local microbial community and fit the neutral model for each local community. The *p*‐value from the chi‐squared test is then utilized to determine whether or not Tang and Zhou’s (2013) hybrid model is suitable for a set of microbial communities sampled from the multiple microbiome sites of a human individual. Specifically, at the metacommunity level, if *p*‐value > .05, then the metacommunity satisfies the NNH and the metacommunity assembly is co‐driven by both niche and neutral processes, which also implies that the metacommunity itself does not satisfy the neutral theory, but within each niche, the local community is neutral. If *p*‐value < .05, the metacommunity does not satisfy the NNH, which also implies that within each niche, the local community is not neutral either, and the metacommunity assembly is solely influenced by the niche process.

It should be noted that the choice of threshold *p*‐value (=.05) in testing the goodness of fitting to the NNH model here and also in the MSN previously is somewhat arbitrary, which was based on conventions (usually the recommendations by the model inventors) in testing the neutral theory or niche‐neutral hybrid models (*e.g*., Hubbell, 2001, Harris et al., 2017, Tang & Zhou, 2013). Occasionally, a different *p*‐value (such as .01 or .001) may be chosen, and the choice may influence the test results slightly. Caution in interpreting the results should be taken accordingly because different *p*‐values may influence the number of “small probability events” of committing an error in testing the fitting of the theoretical models. In the case of this study, since we used the same threshold *p*‐values for both MSN and NNH, the potential bias or error level for small probability events for both neutral and niche effects is therefore the same or similar. Therefore, from the perspective of evaluating the relative significance between the neutral drifts and niche differentiations, the potential influence of different thresholds of *p*‐values should be minimal.

### 2.4 Logistic regression and Cox regression modeling for the relationships among neutrality, niche differentiations, and key community/species characteristics

The previously described MSN/NNH modeling approaches are based on the *theoretically* derived MSN (Harris et al., 2017) and NNH (Tang & Zhou, 2013) models, which allow for testing their goodness of fitting to the human microbiome datasets and assessing the relative importance of stochastic neutral forces (drift and dispersal) and deterministic selection forces (niche differentiations). To deal with the possibility that neither MSN nor NNH can fit the datasets satisfactorily, a contrastingly different modeling strategy from the previous sections, that is, a data‐driven approach, is adopted to evaluate the factors influencing the performance of MSN/NNH models. The potential insights from this alternative approach can help to understand how key community/species characteristics may influence the neutrality and/or niche differentiations.

The standard logistic regression (LR) (Kleinbaum & Klein, 2010) or Cox regression (Cox proportional hazard model) (Kleinbaum & Klein, 2012), if the former is not applicable, is used to conduct the above‐designed analysis. The reason why both the regression approaches (rather than regular regression approaches) were selected is because of the fact that the prediction from the LR/Cox modeling is probability, which is advantageous in dealing with the goodness‐of‐fitting status of MSN/NNH models. The community/species characteristics that are selected to build the logistic regression or Cox model were as follows: community/species dominance (Ma & Ellison 2018, 2019), community diversity measured in Hill numbers, *θ* (the fundamental biodiversity number), *M* (migration rate from the MSN model), *X* (the ratio of birth to death in the NNH model), and *Y* (migration rate from the NNH model), and the passing status of MSN/NNH at local and metacommunity levels.

## 3 RESULTS

### 3.1 MSN (multisite neutral) and NNH (niche‐neutral hybrid) modeling

The full test results for the MSN (multisite neutral) model are listed in Tables S3–S8 in Online Supplementary Information (I) (OSI‐1) with the six human microbiome datasets (*see* Table 1), one supplementary table for each of the six datasets. The selected *19* samples from bulky Tables S3–S8 are listed in Table S1 to facilitate the explanation. Similarly, only the selected results of *19* samples from Tables S9–S14 are listed in Table S2, which exhibited the full results from the fitting to the NNH model to the six datasets. To prepare Tables S1 and S2, I selected *4* batches of samples (*i.e*., four metacommunities) from each of the 6 human microbiome datasets, corresponding to 4 possible combinations of both models (*i.e*., passing MSN only, passing NNH only, passing both, or passing none). With this scheme, a maximum total of 24 (4x6) batches of samples could be selected, and it turned out that some of the combinations were missing from the results, leading to *19* samples being selected in Tables S1 and S2. The interpretations for the table columns were noted at the bottom sections of those results tables (*i.e*., Tables S1–S2, Tables S3–S14). Therefore, Tables S1 and S2 offer windows to inspect the parameters and conclusions of testing the MSN/NNH models. To inspect the complete test results of the 699 metacommunity samples, readers are asked to refer Tables S3–S14, which are supplied in Online Supplementary Information (II) file or OSI‐2.

With Harris et al.'s (2017) multisite HDP‐UNTB model (*i.e*., MSN model), two‐level tests (local community and metacommunity levels) for the neutrality were performed. For both the tests, samples were generated from ***N*** = 2,500 sets of fitted parameters, which were selected from every tenth iteration of the last 25,000 Gibbs samples (a total of 50,000 samples were simulated and the first 25,000 samples were discarded as burn‐in). ***N*** = 2,500 is chosen to compute the pseudo‐*p*‐values for conducting the neutrality test. To test the neutrality at the metacommunity level, assume ***L_M_*** is the median of the log‐likelihoods of the simulated neutral metacommunity samples, and ***N_M_*** is the number of simulated neutral metacommunity samples, having their likelihoods satisfying ***L* ≤ *L_0_***, where ***L*** is the simulated likelihood, and ***L_0_*** is the *actual log‐likelihood L_0_*, then the ***P_M_*** = ***N_M_*/*N*** is the pseudo‐*p*‐value for testing the neutrality at the metacommunity level. If ***P_M_*** > 0.05, the metacommunity satisfies the MSN model. Similarly, to test the neutrality at the local community level, a pseudo‐*p*‐value ***P_L_*** is computed. If ***P_L_*** > 0.05, the local community satisfies the neutral model (*see* Tables S3–S8 (full results) and Table S1 (selected samples for better explanation) for fitting the MSN model).

With Tang and Zhou's (2013) NNH model (*see* Tables S9–S14 for the detailed results and Table S2 for the selected samples), the chi‐squared test was used to compute the chi‐square value and the associated *p*‐value. If the *p*‐value > .05, the metacommunity is judged to satisfy the NNH model, indicating that the metacommunity is primarily differentiated into multiple neutral local communities. Furthermore, the last two table columns of the NNH results show the number and percentage of local communities (niches) that passed the local neutrality test.

**FIGURE 3:**
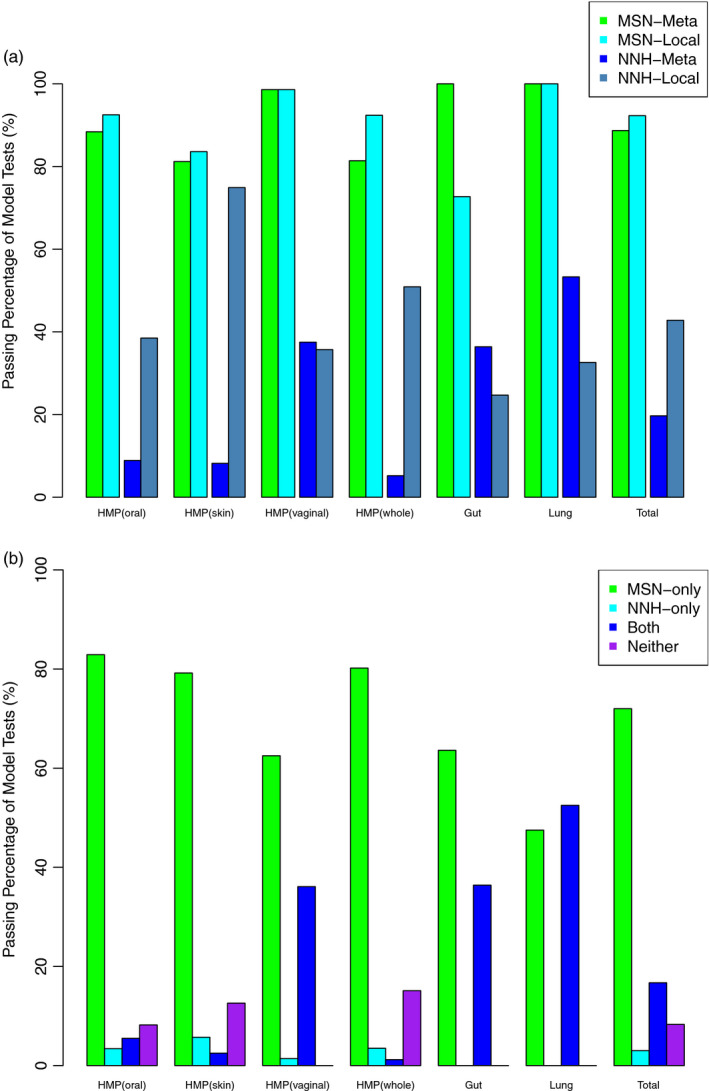
(a): Bar charts showing the passing percentages of testing the MSN or NNH for each human microbiome dataset, respectively: For each group (dataset), the passing percentages for both metacommunity and local community of each model (MSN or NNH) were exhibited (*see* Table 3A for detailed numerical information). (b) Bar charts showing the passing percentages of samples that passed MSN only, NNH only, both MSN and NNH, and none, for each human microbiome group (dataset), respectively (*see* Table 3B for detailed numerical information). Note that the gut and lung datasets were from two projects different from the other HMP datasets, and caution should be taken in interpreting their differences. In other words, the differences among the three groups of datasets might be complicated by possibly different experimental procedures such as sequencing platform

I now attempt to draw a big picture from the test results by computing the statistics of the passing rates for both MSN and NNH models. Recall that they use the exact same data formats, or the community versus metacommunity definitions. For example, with the gut dataset, 11 subjects represent 11 metacommunities, and each metacommunity contains 7 local communities (niches in the case of NNH) given that each subject was physically sampled at 7 gut sites. Table 2 (also see Figure 3a) below shows the passing rates for testing the MSN and NNH models, on the left and right sections, respectively. For each model, the passing rate at metacommunity and local community level is listed separately.

**TABLE 2:** The passing percentages from testing the MSN (multisite neutral model) and NNH (niche‐neutral hybrid model), respectively, with the six human microbiome datasets, summarized from Tables S3–S14 included in the OSI‐2 (Supplementary Information S2)^a^

Human microbiome	*N*	MSN (multisite neutral)				NNH (niche‐neutral hybrid)			
		Metacommunity		Local community		Metacommunity		Local community	
		*N* (pass)	%	*N* (pass)	%	*N*	%	Avg. *N* (Pass)	Avg. %
HMP (oral)	146	129	88.4	135	92.5	13	8.9	4	38.5
HMP (skin)	159	130	81.2	133	83.6	13	8.2	3	74.9
HMP (vaginal)	72	71	98.6	71	98.6	27	37.5	1	35.7
HMP (total)	172	140	81.4	159	92.4	9	5.2	8	50.9
Gut	11	11	100	8	72.7	4	36.4	2	24.7
Lung	139	139	100	139	100	72	53.3	1	32.6
Total	699	620	88.7	645	92.3	138	19.7	NA	42.8

^a^ *N* is the number of local communities or metacommunities that passed the MSN or NNH test; % is the percentage that passed the test. *See* Figure 3a for the graphic display of the percentage results in this table. ^a^

As expected, the passing rates for neutrality tests at local community level are higher than those at the metacommunity level. The difference is approximately 4% for the MSN and 23% for the NNH, respectively. This should be expected given that local communities are more homogenous in their habitats. In the case of MSN, it indicates that neutrality is more likely to maintain at the local community scale than at the larger metacommunity scale.

**FIGURE 2:**
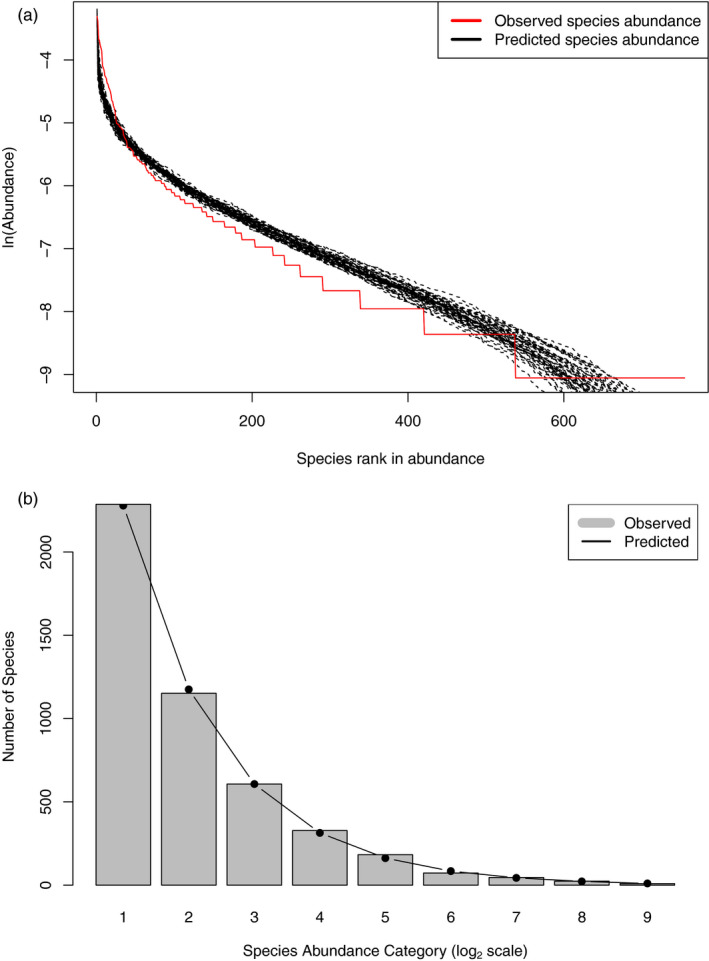
(a) An example of gut microbial metacommunity consisting of 7 local communities (*i.e*., subject #407 was sampled at 7 locations of his gut) and showing successful fitting to the MSN (multisite neutral) model. (b) An example of gut microbial metacommunity consisting of 7 local communities (Subject#404 was sampled at 7 locations of his gut) and showing successful fitting to the NNH (niche‐neutral hybrid) model (both subjects are from Zhang et al., 2014, *see* Table 1)

The results of testing the NNH model (*see* Figure 2b for an example of model fitting) reveal two points: (a) Significant proportions of local communities (42.8%) did pass the neutrality test, which is still much lower than the local neutrality percentage from testing the MSN model (92.3%). Therefore, both MSN and NNH crossly confirmed that nearly half of the *local* communities exhibit patterns of species abundance distribution indistinguishable from neutral. (b) While 42.8% local communities passed the neutrality test with the NNH model, only 19.7% passed the NNH at the metacommunity level. That is, 19.7% of metacommunities exhibited niche differentiations at the global (metacommunity) scale, while exhibiting neutral patterns simultaneously at the local community scale. In other words, these metacommunities passing the NNH model test consist of niche‐differentiated “neutral” patches—within‐patch pattern is indistinguishable from neutral.

The above point (*ii*) suggested that the relationship between local and metacommunity in the NNH setting could be more complex than expected by the model inventor (Tang & Zhou, 2013). From the perspective of NNH, given that 42.8% *local* communities passed neutrality test with the NNH, but only 19.7% of their metacommunity passed the NNH hybrid model at the *meta*community level, how should the difference of 23.1%(=42.8–19.7) between local and metacommunity levels is interpreted? In other words, are those 23.1% metacommunities neutral or non‐neutral? Here, I try to shed light on the difference from an alternative MSN perspective. The MSN modeling suggested that the neutrality passing rates were 92.3% and 88.7% at the local community level and metacommunity level, respectively. In other words, the MSN modeling shows that the neutrality at either local or metacommunity level exceeded 88.7%. Based on the neutrality estimation from the MSN modeling, I infer that the previously mentioned 23.1% gap between local and metacommunity exhibited by the NNH model is very likely neutral or more precisely indistinguishable from neutral metacommunities.

Further count the numbers (and percentages) of the samples that passed MSN only, NNH only, MSN and NNH, and neither of them, and the breakup is listed in Table 3 (also see Figure 3b). Overall, there were nearly 24 times more samples successfully fitted the MSN model exclusively than those successfully fitted the NNH model exclusively (72% vs. 3%; Table 3). There were 16.7% samples (metacommunities) that fitted to both MSN and NNH successfully. Theoretically, this percentage fitted to both MSN and NNH models successfully should be rather small or even zero, given that the assumptions of both models are different. I do not have a definite explanation other than those repeatedly debated in the literature of neutral theory testing (*e.g*., the same observed pattern may be explained by multiple mechanisms; *see* review by Rosindell et al., 2011, 2012, Tang & Zhou, 2013). This proportion is “troubling,” but fortunately relatively small. It suggests that, for about 16.7% metacommunities, my integrated MSN/NNH approach is unable to cross‐validate each other's results, which is further elaborated in Discussion section. Other than identifying this potentially “troubling” 16.7% of overlap of MSN and NNH modeling, the other categories in Table 3 and Figure 3b displayed the cross‐verifications of both the modeling approaches.

**TABLE 3:** Comparative summary of the performances of MSN and NNH models fitted to the human microbiome datasets of 699 metacommunities, summarized from Tables S3–S14 included in the OSI‐2 (Supplementary Information S2)^a^

Microbiome	Individuals	Passing MSN only		Passing NNH only		Passing both MSN and NNH		Passing None (MSN, NNH)	
		*N*	%	*N*	%	*N*	%	*N*	%
HMP (oral)	146	121	82.9	5	3.4	8	5.5	12	8.2
HMP (skin)	159	126	79.2	9	5.7	4	2.5	20	12.6
HMP (vaginal)	72	45	62.5	1	1.4	26	36.1	0	0.0
HMP (total)	172	138	80.2	6	3.5	2	1.2	26	15.1
Gut	11	7	63.6	0	0.0	4	36.4	0	0.0
Lung	139	66	47.5	0	0.0	73	52.5	0	0.0
Total	699	503	72.0	21	3.0	117	16.7	58	8.3

^a^ *N* is the number of local community or metacommunity that passed the MSN and/or NNH test; % is the percentage that passed the test. *See* Figure 3b for the graphic display of the percentage results in this table. ^a^

### 3.2 Logistic/Cox regression analysis

With the selected community/species characteristics (indexes) and performance metrics (passing status) from testing the MSN/NNH models, I use logistic regression (LR) to investigate the relative importance of key community/species‐level characteristics in affecting the performance of MSN/NNH models. The LR analysis allows for identifying significant factors (*i.e*., microbiome characteristics) that are strongly related to the balance between stochastic neutral versus deterministic selective niche forces. Note that some of the factors are the exhibitions of neutral or niche effects, rather than the causes, and hence, the term “related” is used.

I first identified the microbiome characteristics (factors) that are strongly related to the neutrality at the meta‐community level by using the LR with subset selection option (Table S15). Four significant factors, community dominance, species richness (Hill numbers at q=0), the fundamental biodiversity number (q) and the neutrality of local community were judged to be significant (p<0.0001) in influencing the niche‐neutral balance. The first two factors were positively related to the non‐neutrality at the meta‐community level. That is, high dominance or high species richness are negatively correlated with meta‐community neutrality. The concept and metric of community and species dominance were proposed by Ma & Ellison (2018, 2019), and high community dominance indicates lower community evenness (or heterogeneity). Obviously, this positive relationship between community dominance and non‐neutrality should be expected because high dominance may signal strong asymmetric interactions, i.e., a property of non‐neutrality or the opposite of neutrality. Similarly, the other two negative relationships (fundamental biodiversity number q and species dominance) displayed in Table S15 should be expected. Table S17 showed the performance of the logistic model described in Table S15 in predicting the neutrality at the meta‐community level. The overall precision of predicting the meta‐community neutrality with the four characteristics (factors) was 91.6%. Table S17 also included the prediction of the LR modeling at the local community level (see Table S16) as explained below.

For the MSN at the local community level, community dominance, species dominance, immigration rate (*M*‐value), and the fundamental biodiversity number (*θ*) were identified to be significant in influencing the neutrality at local community level (Table S16). The precision level of predicting local neutrality was 96.4%, which is higher than that at the metacommunity level (Table S17).

For the NNH model, Table S18 shows that community dominance, birth‐to‐death ratio, migration of each niche, and neutrality at local community level significantly influence the probability of passing NNH model at metacommunity level (*i.e*., the niche differentiations at the metacommunity level). Table S19 shows that the prediction precision of the LR modeling for NNH model at the local community level was 91.9%.

As to the testing of the local community neutrality with the NNH model, I adopted Cox regression model (with variable subset selection), rather than the LR, given that the response variable is not 0/1 at the local community level (LR is only applicable for 0/1 variable) (Table S20). Table S20 shows that community dominance, birth/death ratio, *M*‐value (migration rate from MSN model), species richness, Shannon index, and the success of NNH model at metacommunity level are significant factors in influencing the neutrality at the local community level with the NNH modeling. Although I successfully built the Cox regression model, given the high standard error associated with the regression coefficient of *M*, I caution that its interpretation needs further investigation, and therefore, no local community‐level prediction was made in this case.

## 4 DISCUSSION

As shown in Figure 1, the two primary objectives and a third minor one were as follows: (a) to evaluate the niche‐neutral continuum; (b) to infer the biogeographic implications of (a); and (c) to discuss the influence of major community/species characteristics on the continuum. Here, we continue to discuss the first two objectives by putting them in the context of the existing literature. In addition, we also discuss some miscellaneous but relevant topics including the microbiome habitat‐specific issues and the comparisons with what I termed "single‐site" neutral model—fitting the Hubbell (2001) neutral theory model to the species abundance distribution data obtained from a single microbiome sample, which has been used in virtually all existing studies on the microbiome.

### 4.1 The relative importance (balance) between niche and neutral forces in shaping niche‐neutral continuum

The truly multisite microbiome datasets, in which dispersal (migration) can occur in ecological times, may even be in vivo (*e.g*., from one skin site to another in distance of centimeters), and the truly multisite sampling model with Harris et al.'s (2017) HDP‐MSN allowed us for a more comprehensive and reliable testing of Hubbell's UNTB. Similarly, the datasets were also ideal for testing Tang and Zhou’s (2013) NNH model, for testing both the MSN and NNH simultaneously (using the exactly same datasets), and complementing and cross‐verifying each other's results. With 5,420 local community samples belonging to 699 metacommunities, my tests with the MSN model revealed significant effects of neutral forces in driving community assembly and in maintaining community diversity, with approximately 89% metacommunities and 92% local communities exhibiting indistinguishable patterns from neutral. The tests with NNH model with the same datasets revealed that approximately 43% of local communities satisfied the neutral model, of which approximately 20% satisfied the hybrid metacommunity model. Both tests confirmed that significant percentage (>43%) of local communities is neutral (Table 2).

A further cross‐model (MSN and NNH) comparative perspective (Table 3) indicated that at the metacommunity level, there are approximately 8% metacommunities, for which both MSN and NNH failed to fit. This simply indicates the complexity of the problem, and further analysis with more powerful and flexible models is needed. Alternative data‐driven modeling approaches with the LR and Cox model (Tables S15–S10 in the OSI‐1) may offer insights into designing more comprehensive and powerful models for interpreting the human microbiome assembly. Those data‐driven models may identify the community/species‐level characteristics that significantly influence the model performance such as MSN and NNH, which can be particularly useful when more datasets are accumulated since LR is a primitive method of powerful deep learning.

Table 2 shows that there were 16.7% samples that successfully fitted to both MSN and NNH, which is actually somewhat troubling; that is, the same observed pattern might be interpreted by two different mechanisms (neutral vs. niche differentiation). Fortunately, this number 16.7% is moderate and indicates the relative robustness of the integrated MSN/NNH modeling approach. Given that up to 88.7% metacommunities and up to 92.3% local communities passed rigorous MSN tests, obviously, neutral forces (*drift* and *dispersal*) play a significant role in driving community assembly and diversity maintenance, and shaping the biogeography of the human microbiome. Since dispersal may not be wholly neutral and may even be treated as an adaptive mechanism to niche differentiations, this study cannot assess the exact level of dispersal in shaping the community assembly. Alternative modeling approaches such as those developed by Janzen et al. (2015) can be used to distinguish multiple syndromes of dispersal.

According to the Gilbert and Levine (2017) experimental study, the importance of ecological drift in structuring diversity in fragmented ecosystems is far greater than predicted by neutral models. Svensson et al. (2018) emphasized that ecological drift can operate even if species are not completely equivalent, and consequently, species are not strictly neutral. Neutral theory of biodiversity assumes that dispersal is stochastic and equivalent among species. Lowe and McPeek (2014), and also Clark (2009) argued that the neutral dispersal assumption should be rejected on principle because dispersal can be a species‐specific process (property), and it evolves by natural selection. However, there is little doubt that dispersal can be “partially neutral” in the sense that stochastic and extrinsic forces influence dispersal in many species and systems, and dispersal affects biodiversity independent of adaptive mechanisms of coexistence. Liu and Zhou (2011), through simulation, showed that asymmetric dispersal could lead to deviations from neutrality. These existing arguments suggest that, if I use the neutrality levels revealed by MSN and NNH as educated guess of the drift and dispersal, my estimation should be on the safe side (conservative).

### 4.2 Cosmopolitanism versus endemism debate and microbial biogeography

The traditional view of Bass‐Becking (1934) doctrine—“*Everything is everywhere, but the environment selects who stays locally*”—was largely based on some general traits microbes posses including tiny individual sizes, large population sizes, fast reproduction, consequently their easy long‐distance dispersal, and low chance of local extinction (Barreto et al., 2014; van der Gast, 2015). Obviously, the interpretation of this characterization depends on the interpretations of terms such as dispersal and drifts, on which the traditional doctrine never specified preciously. In terms of traditional interpretations, where drift and dispersal were considered as totally stochastic and neutral, then biogeography of microbes specified by Bass‐Becking doctrine should be random, cosmopolitan, and determined by environment heterogeneity. Then, the doctrine is essentially a synthesis of *neutral dispersal* and *species sorting* in metacommunity theory and the later is essentially another incarnation of traditional niche theory. The cosmopolitan nature of microbial distribution should exhibit a very different biogeography from those of plants and animals. Some researchers consider that microbes do not have a biogeography given microbes are more like passive “dust” and they can disperse to wherever they can be “tolerated.” Nevertheless, recent studies presented counterevidence against the traditional view and suggest that there are microbial biogeography patterns that are not random, but the difficulty lies in assessing the factors that determine the geographic distribution, whether they are historical evolutionary events (geographic barriers) or contemporary ecological environmental factors (Barreto et al., 2014). Therefore, the issue is far from settled.

Besides the practical measurement issues related to evolutionary/ecological timescales, the interpretation of Bass‐Becking can be different, for example, if dispersal is considered as only partially neutral. This further complicates the investigation of microbial biogeography, but obviously, the complexity cannot be avoided. To tackle the complexity, van der Gast (2013, 2015) revised Baas‐Becking (1934) hypothesis as “*Some things are everywhere and some things are not. Sometimes the environment selects and sometimes it doesn't*.” This is essentially a hybrid "compromise" between cosmopolitanism (globally random dispersers) and endemism (biogeographically restricted natives or niches) schools of microbial biogeography. The results cast strong supporting evidence to van der Gast (2013, 2015) recent revision to Bass‐Becking doctrine, which I believe to represent the state‐of‐the‐art first principle in microbial biogeography. Specifically, my conclusions from the integrated tests with MSN and NNH models offer a possibility to quantify the level of “*some*” in the revised doctrine, as suggested by the passing percentages discussed previously. In other words, since van der Gast (2015) revision to Baas‐Becking doctrine is essentially a hybrid representation of dispersal and selection, the integrated test approach I demonstrated can be harnessed to determine the balance of the two forces. Specifically, if I accept the neutrality level of at least ½ from previously discussed MSN/NNH modeling, my educated guess for van der Gast (2013, 2015) “some” should be a half of time or occasions, at the minimum.

I further hypothesize that there is room for both evolutionary and ecological forces to shape the distribution of biodiversity (Hanson et al., 2012), and perhaps the real challenge is to determine at what scale the cosmopolitan or endemism (native) dominates. It is very likely that the *scale* may be a continuum given that the *evolutionary time* and *ecological time* of microbes may overlap with each other partially because microbes reproduce much faster (20 min could be enough for bacterial to complete one generation) and their evolution is on fast tracks (*e.g.,* Baym et al., 2016). In a follow‐up study, I will further explore this topic by analyzing time‐series data of microbial community dynamics.

Quantifying dispersal is not easy. As mentioned previously, dispersal is often treated as purely stochastic and extrinsically controlled, including neutral theory of biodiversity. There is a need to investigate the relative importance of neutral and adaptive forces in shaping individual dispersal propensities and distances, population‐level dispersal distributions, and resulting effects on populations and communities (such as population density or community diversity) (Lowe & McPeek, 2014). Again, Janzen et al.'s (2015) approach is worthy of trying in quantifying dispersal from a multidimensional perspective.

### 4.3 Habitat‐specific differences in the human microbiomes

Here, I briefly discuss the differences among five major microbiome habitats in their passing rates with both MSN and NNH testing, and consequently, the relative balance between stochastic neutral forces (drift and dispersal) and deterministic niche forces. Regarding the neutrality test with the MSN model, the neutrality passing rates among different microbiome habitats ranged between 81.4% and 100% at the metacommunity level, and 72.7 and 100% at the local community level. In consideration of their differences in sample sizes (*N* = 11–172; Table 2) and possible differences in metagenomic sequencing operations, I do not draw any further conclusion about the apparent differences.

Regarding testing the niche differentiations among local communities with the NNH model, the oral and skin exhibited the passing rates of NNH lower than 10% (5.2%‐8.9%), while vaginal, gut, and lung exhibited much higher passing rates of NNH (36.4%‐53.3%). The face value of this contrasting difference is that the latter three microbiome habitats should have bigger niche differentiations internally. This face value should be true, at least, for the gut, which is obviously the most highly differentiated microbiome habitat, and also host the most complex part of the human microbiome.

### 4.4 Comparisons with existing studies on the neutrality of human microbiomes

Given the huge difference between the overall passing rates of testing the MSN model at either local community (92.3%) or metacommunity (88.7%) with that (1%) reported previously by Li and Ma (2016), one would certainly wonder what was wrong? I further compared the computed migration rates for the same HMP‐whole dataset with the three approaches including MSN, NNH and previously used single‐site UNTB, and the mean migration rates for the three models are 0.069 [0.022, 0.125], 0.0005 [0, 0.006] and 0.995 [0.349, 1], respectively (the ranges are parenthesized). The previously reported migration rate by Li and Ma (2016), which used the single‐site sampling formula (Etienne, 2005, 2007, 2009; Hankin, 2007), is much larger than that from MSN or NNH models obtained in this study. This result confirmed the validity of Harris et al.'s (2017) recommendation for using their HDP (hierarchical Dirichlet process)‐based MSN model. Tang and Zhou (2013) study further verified Harris et al.'s (2017) recommendation. I therefore conclude that the neutrality of the human microbiome is rather strong given that approximately 89% metacommunities passed the MSN test, and even more (approximately 92%) local communities were neutral (Table 2). As to the comparisons with other existing studies on the human microbiome neutrality, majority of the previous studies obtained similar conclusions as Li and Ma (2016), and were discussed there. A general suggestion is that Harris et al.'s (2017) HDP‐MSN fitting framework for the UNTB should be utilized whenever multisite data are available.

## CONFLICT OF INTERESTS

The author declares no competing interests.

## Supporting information

Supplementary Material

Supplementary Material

## Data Availability

All datasets analyzed in this study are available in public domain and see Table 1 for the access information. No new datasets are generated from this study.

## References

[eva13116-bib-0001] Adams, R. I. , Miletoo, M. , Taylor, J. W. , & Bruns, T. D. (2013). Dispersal in microbes: fungi in indoor air are dominated by outdoor air and show dispersal limitation at short distances. The ISME Journal. 7, 1262–1273. 23426013 10.1038/ismej.2013.28 PMC3695294

[eva13116-bib-0002] Baas‐Becking, L. G. M. (1934). In W. P. Van Stockum , & N. V. Zoon (Eds.), Geobiologie of inleiding tot de milieukunde.

[eva13116-bib-0003] Barreto, D. P. , Conrad, R. , Klose, M. , Claus, P. , & Enrich‐Prast, A. (2014). Distance‐decay and taxa‐area relationships for bacteria, archaea and methanogenic archaea in a tropical lake sediment. PLoS One, 9(10), e110128 10.1371/journal.pone.0110128 25330320 PMC4203765

[eva13116-bib-0004] Baym, M. , Lieberman, T. D. , Kelsic, E. D. , Chait, R. , Gross, R. , Yelin, I. , & Kishony, R. (2016). Spatiotemporal microbial evolution on antibiotic landscapes. Science, 353(6304), 1147–1151. 10.1126/science.aag0822 27609891 PMC5534434

[eva13116-bib-0006] Carbonero, A. , Paniagua, J. , Torralbo, A. , Arenas‐Montes, A. , Borge, C. , & García‐Bocanegra, I. (2014). Campylobacter infection in wild artiodactyl species from southern Spain: Occurrence, risk factors and antimicrobial susceptibility. Comparative Immunology, Microbiology and Infectious Diseases, 37(2), 115–121. 10.1016/j.cimid.2014.01.001 24462184

[eva13116-bib-0007] Chen, H. , Peng, S. , Dai, L. , Zou, Q. , Yi, B. , Yang, X. , & Ma, Z. (2017). Oral microbial community assembly under the influence of periodontitis. PLoS One, 12(8), e0182259 10.1371/journal.pone.0182259 28813450 PMC5558961

[eva13116-bib-0008] Clark, J. S. (2009). Beyond neutral science. Trends in Ecology & Evolution, 24, 8–15. 19026462 10.1016/j.tree.2008.09.004

[eva13116-bib-0009] Costello, E. K. , Stagaman, K. , Dethlefsen, L. , Bohannan, B. J. M. , & Relman, D. A. (2012). The application of ecological theory toward an understanding of the human microbiome. Science, 336, 1255–1262. 22674335 10.1126/science.1224203 PMC4208626

[eva13116-bib-0010] Curtis, T. P. , & Sloan, W. T. (2004). Prokaryotic diversity and its limits: Microbial community structure in nature and implications for microbial ecology. Current Opinions in Microbiology, 7, 221–226. 10.1016/j.mib.2004.04.010 15196488

[eva13116-bib-0011] Dai, L. , Kou, H. , Xia, Y. , Wen, X. , Gao, J. , & Ma, Z. (2017). Does colorectal cancer significantly influence the assembly of gut microbial communities? PeerJ, 5(8), e3383 10.7717/peerj.3383 28674643 PMC5493029

[eva13116-bib-0012] Etienne, R. S. (2005). A new sampling formula for neutral biodiversity. Ecology Letters, 8(3), 253–260. 10.1111/j.1461-0248.2004.00717.x

[eva13116-bib-0013] Etienne, R. S. (2007). A neutral sampling formula for multiple samples and an 'exact' test of neutrality. Ecology Letters, 10(7), 608–618. 10.1111/j.1461-0248.2007.01052.x 17542939

[eva13116-bib-0014] Etienne, R. S. (2009). Maximum likelihood estimation of neutral model parameters for multiple samples with different degrees of dispersal limitation. Journal of Theoretical Biology, 257, 510–514. 10.1016/j.jtbi.2008.12.016 19168078

[eva13116-bib-0015] Fisher, C. K. , & Mehta, P. (2014). The transition between the niche and neutral regimes in ecology. Proceedings of the National Academy of Sciences of the United States of America, 111(36), 13111–13116. 25157131 10.1073/pnas.1405637111 PMC4246938

[eva13116-bib-0016] Foissner, W. (2006). (2006) Biogeography and dispersal of microorganisms: A review emphasizing protists. Acta Protozool., 45, 111–136.

[eva13116-bib-0017] Gilbert, B. , & Levine, J. M. (2017). Ecological drift and the distribution of species diversity. Proceedings of the Royal Society B, 284, 20170507 10.1098/rspb.2017.0507 28566486 PMC5454268

[eva13116-bib-0018] Gotelli, N. J. , & McGill, B. J. (2006). Null versus neutral models: What’s the difference? Ecography, 29, 793–800. 10.1111/j.2006.0906-7590.04714.x

[eva13116-bib-0019] Grossart, H. P. , Dziallas, C. , Leunert, F. , & Tang, K. W. (2010). Bacteria dispersal by hitchhiking on zooplankton. Proceedings of the National Academy of Sciences of the United States of America, 107(26), 11959–11964. 10.1073/pnas.1000668107 20547852 PMC2900670

[eva13116-bib-0020] Haegeman, B. , & Etienne, R. S. (2017). A general sampling formula for community structure data. Methods in Ecology and Evolution, 8, 1506–1519. 10.1111/2041-210X.12807

[eva13116-bib-0021] Hankin, R. K. S. (2007). Introducing UNTB, an R package for simulating ecological drift under the unified neutral theory of biodiversity. Journal of Statistical Software, 22(12):15.

[eva13116-bib-0022] Hanson, C. A. , Fuhrman, J. A. , Horner‐Devine, M. C. , & Martiny, J. B. H. (2012). Beyond biogeographic patterns: Processes shaping the microbial landscape. Nature Reviews Microbiology, 10, 497–506. 10.1038/nrmicro2795 22580365

[eva13116-bib-0023] Harris, K. , Parsons, T. L. , Ijaz, U. Z. , Lahti, L. , Holmes, I. , & Quince, C. (2017). Linking statistical and ecological theory: Hubbell's unified neutral theory of biodiversity as a hierarchical Dirichlet process. Proceedings of the IEEE, 105(3), 516–529.

[eva13116-bib-0024] He, F. L. , Zhang, D. Y. , & Lin, K. (2012). Coexistence of nearly neutral species. Journal of Plant Ecology, 5, 72–81. 10.1093/jpe/rtr040

[eva13116-bib-0025] HMP Consortium (2012). A framework for human microbiome research. Nature, 486(7402), 215. 22699610 10.1038/nature11209 PMC3377744

[eva13116-bib-0026] Hu, X. S. , He, F. L. , & Hubbell, S. P. (2006). Neutral theory in macro‐ecology and population genetics. Oikos, 113, 548–556. 10.1111/j.2006.0030-1299.14837.x

[eva13116-bib-0027] Hubbell, S. P. (2001) The unified neutral theory of biodiversity and biogeography. Princeton University Press. 10.1016/j.tree.2011.03.024 21561679

[eva13116-bib-0028] Hubbell, S. P. (2005). Neutral theory in community ecology and the hypothesis of functional equivalence. Functional Ecology, 19, 166–172. 10.1111/j.0269-8463.2005.00965.x

[eva13116-bib-0029] Hubbell, S. P. (2006). Neutral theory and the evolution of ecological equivalence. Ecology, 87, 1387–1398. 10.1890/0012-9658(2006)87[1387:NTATEO]2.0.CO;2 16869413

[eva13116-bib-0030] Janzen, T. , Haegeman, B. , & Etienne, R. S. (2015). A sampling formula for ecological communities with multiple dispersal syndromes. Journal of Theoretical Biology, 387(21), 258–261. 10.1016/j.jtbi.2015.10.001 25816742

[eva13116-bib-0031] Jeraldo, P. , Sipos, M. , Chia, N. , Brulc, J. M. , Dhillon, A. S. , Konkel, M. E. , Larson, C. L. , Nelson, K. E. , Qu, A. , Schook, L.B. , Yang, F. , White, B. A. , & Goldenfelda, N. (2012). Quantification of the relative roles of niche and neutral processes in structuring gastrointestinal microbiomes. Proceedings of the National Academy of Sciences of the United States of America, 109(25), 9692–9698. 22615407 10.1073/pnas.1206721109 PMC3382495

[eva13116-bib-0032] Kalyuzhny, M. , Seri, E. , Chocron, R. , Brulc, J. M. , Singh Dhillon, A. , Konkel, M. E. , Larson, C. L. , Nelson, K. E. , Qu, A. , Schook, L. B. , Yang, F. , White, B. A. , & Goldenfeld, N. (2014). Niche versus neutrality: A dynamical analysis. American Naturalist, 184(4), 439–446. 10.1086/677930 25226179

[eva13116-bib-0033] Kleinbaum, D. G. , & Klein, M. (2010). Logistic Regression. Springer.

[eva13116-bib-0034] Kleinbaum, D. G. , & Klein, M. (2012). Survival analysis. Springer.

[eva13116-bib-0035] Li, L. W. , & Ma, Z. S. (2016). Testing the neutral theory of biodiversity with human microbiome datasets. Scientific Reports. 6, Article No. 31448. 27527985 10.1038/srep31448 PMC4985628

[eva13116-bib-0036] Liu, J. , & Zhou, S. (2011). Asymmetry in species regional dispersal ability and the neutral theory. PLoS One, 6(8), e24128 10.1371/journal.pone.0024128 21901163 PMC3162033

[eva13116-bib-0037] Lowe, W. H. , & McPeek, M. A. (2014). Is dispersal neutral? Trends in Ecology & Evolution, 29(6), 444–450. 24962790 10.1016/j.tree.2014.05.009

[eva13116-bib-0038] Lozupone, C. , Faust, K. , Raes, J. , Faith, J. J. , Frank, D. N. , Zaneveld, J. , Gordon, J. I. , & Knight, R. (2012). Identifying genomic and metabolic features that can underlie early successional and opportunistic lifestyles of human gut symbionts. Genome Research, 22(10), 1974–1984. 10.1101/gr.138198.112 22665442 PMC3460192

[eva13116-bib-0039] Ma, Z. S. (2017) Bioinformatics: Computing and Software. Science Press. ISBN 978‐7‐03‐042639‐0.

[eva13116-bib-0040] Ma, Z.S. (2019). A new DTAR (diversity–time–area relationship) model demonstrated with the indoor microbiome. Journal of Biogeography, 46(9), 2024–2041. 10.1111/jbi.13636

[eva13116-bib-0041] Ma, Z. S. (2020). Critical network structures and medical ecology mechanisms underlying human microbiome‐associated diseases. iScience, 23(6), 101195 10.1016/j.isci.2020.101195 32559728 PMC7303986

[eva13116-bib-0042] Ma, Z. S. , & Ellison, A. M. (2018). A unified concept of dominance applicable at both community and species scale. Ecosphere, 9(11), e02477 10.1002/ecs2.2477

[eva13116-bib-0043] Ma, Z. S. , & Ellison, A. M. (2019). Dominance network analysis provides a new framework for studying the diversity‐stability relationship. Ecological Monographs, 89(2), e01358 10.1002/ecm.1358

[eva13116-bib-0044] Ma, Z. S. , Zhang, C. C. , Zhang, Q. P. , Li, J. , Li, L. , Qi, L. , Yang, X. (2016). A brief review on the ecological network analysis with applications in the emerging medical ecology. Retrieved from http://link.springer.com/protocol/10.1007/8623_2016_204

[eva13116-bib-0045] Martiny, J. B. H. , Bohannan, B. J. M. , Brown, J. H. , Colwell, R. K. , Fuhrman, J. A. , Green, J. L. , Horner‐Devine, M. C. , Kane, M. , Krumins, J. A. , Kuske, C. R. , Morin, P. J. , Naeem, S. , Ovreås, L. , Reysenbach, A.‐L. , Smith, V. H. , Staley, J. T. (2006). Microbial biogeography: Putting microorganisms on the map. Nature Reviews Microbiology, 4, 102–112. 16415926 10.1038/nrmicro1341

[eva13116-bib-0046] Matthews, T. J. , & Whittaker, R. J. (2014). Neutral theory and the species abundance distribution: Recent developments and prospects for unifying niche and neutral perspectives. Ecology & Evolution, 4(11), 2263–2277. 25360266 10.1002/ece3.1092 PMC4201439

[eva13116-bib-0047] May, F. , Giladi, I. , Ziv, Y. , & Jeltsch, F. (2011). Dispersal and diversity–unifying scale‐dependent relationships within the neutral theory. Oikos, 10.1111/j.1600-0706.2011.20078.x

[eva13116-bib-0048] McGill, B. J. (2003). A test of the unified neutral theory of biodiversity. Nature, 422, 881–885. 12692564 10.1038/nature01583

[eva13116-bib-0049] McGill, B. J. , Maurer, B. A. , & Weiser, M. D. (2006). Empirical evaluation of neutral theory. Ecology, 87, 1411–1423. 10.1890/0012-9658(2006)87[1411:EEONT]2.0.CO;2 16869415

[eva13116-bib-0050] Noble, A. E. , & Fagan, W. F. (2015). A niche remedy for the dynamical problems of neutral theory. Theoretical Ecology, 8(1), 149–161.

[eva13116-bib-0051] Ofiteru, I. D. , Lunn, M. , Curtis, T. P. , Wells, G. F. , Criddle, C. S. , Francis, C. A. , & Sloan, W. T. (2010). Combined niche and neutral effects in a microbial wastewater treatment community. Proceedings of the National Academy of Sciences of the United States of America, 107(35), 15345–15350. 20705897 10.1073/pnas.1000604107 PMC2932620

[eva13116-bib-0052] Paisse, S. , Valle, C. , Servant, F. , Courtney, M. , Burcelin, R. , Amar, J. , & Lelouvier, B. (2016). Comprehensive description of blood microbiome from healthy donors assessed by 16S targeted metagenomic sequencing. Transfusion, 56, 1138–1147. 26865079 10.1111/trf.13477

[eva13116-bib-0053] Pigolotti, S. , & Cencini, M. (2013). Species abundances and lifetimes: From neutral to niche‐stabilized communities. Journal of Theoretical Biology, 338(1772), 1–8. 23999281 10.1016/j.jtbi.2013.08.024

[eva13116-bib-0054] Rosindell, J. , Hubbell, S. P. , & Etienne, R. S. (2011). The Unified Neutral Theory of Biodiversity and Biogeography at Age Ten. Trends in Ecology & Evolution, 26, 340–348. 10.1016/j.tree.2011.03.024 21561679

[eva13116-bib-0055] Rosindell, J. , Hubbell, S. P. , He, F. , Harmon, L. J. , & Etienne, R. S. (2012). The case for ecological neutral theory. Trends in Ecology & Evolution, 27, 203–208. 10.1016/j.tree.2012.01.004 22341498

[eva13116-bib-0057] Sloan, W. , Lunn, M. , Woodcock, S. , Head, I. M. , Nee, S. , & Curtis, T. P. (2006). Quantifying the roles of immigration and chance in shaping prokaryote community structure. Environmental Microbiology, 8(4), 732–740. 16584484 10.1111/j.1462-2920.2005.00956.x

[eva13116-bib-0058] Sloan, W. T. , Woodcock, S. , Lunn, M. , Head I. M. , & Curtis T. P. (2007). Modeling taxa‐abundance distributions in microbial communities using environmental sequence data. Microbial Ecology, 53(3), 443–455. 17165121 10.1007/s00248-006-9141-x

[eva13116-bib-0059] Stokes, C. J. , & Archer, S. R. (2010). Niche differentiation and neutral theory: An integrated perspective on shrub assemblages in a parkland savanna. Ecology, 91(4), 1152–1162. 20462129 10.1890/08-1105.1

[eva13116-bib-0060] Svensson, E. I. , Gómez‐Llano, M. A. , Torres, A. R. , & Bensch, H. M. (2018). Frequency dependence and ecological drift shape coexistence of species with similar niches. The American Naturalist, 191(6), 691–703. 10.1086/697201 29750557

[eva13116-bib-0061] Tang, J. , & Zhou, S. (2013). Hybrid niche‐neutral models outperform an otherwise equivalent neutral model for fitting coral reef data. Journal of Theoretical Biology, 317(1), 212–218. 23089372 10.1016/j.jtbi.2012.10.019

[eva13116-bib-0062] Teh, Y. W. , Jordan, M. I. , Beal, M. J. , & Blei, D. M. (2006). Hierarchical Dirichlet processes. Journal of the American Statistical Association, 101(476):1566–1581.

[eva13116-bib-0063] Tilman, D. (2004). Niche tradeoffs, neutrality, and community structure: A stochastic theory of resource competition, invasion, and community assembly. Proceedings of the National Academy of Sciences of the United States of America, 101(30), 10854–10861. 15243158 10.1073/pnas.0403458101 PMC503710

[eva13116-bib-0064] Turnbaugh, P. J. , Ley, R. E. , Hamady, M. , Fraser‐Liggett, C. M. , Knight, R. , & Gordon, J. I. (2007). The human microbiome project. Nature, 449, 804–810. 10.1038/nature06244 17943116 PMC3709439

[eva13116-bib-0065] van der Gast, C. J. (2013). Microbial biogeography and what Baas Becking should have said. Microbiology Today, 40, 108–111.

[eva13116-bib-0066] van der Gast, C. J. (2015). CJ (2015) Microbial biogeography: The end of the ubiquitous dispersal hypothesis? Environmental Microbiology, 17, 3 10.1111/1462-2920.12635 25521363

[eva13116-bib-0067] Vellend, M. (2010). Conceptual synthesis in community ecology. The Quarterly Review of Biology, 85, 183–206. 20565040 10.1086/652373

[eva13116-bib-0068] Venkataraman, A. , Bassis, C. M. , Beck, J. M. , Young, V. B. , Curtis, J. L. , Huffnagle, G. B. , & Schmidt, T. M. (2015). Application of a neutral community model to assess structuring of the human lung microbiome. MBio, 6(1). 10.1128/mBio.02284-14 PMC4324308 25604788

[eva13116-bib-0070] Volkov, I. , Banavar, J. R. , Hubbell, S. P. , & Maritan, A. (2007). Patterns of relative species abundance in rainforests and coral reefs. Nature, 450, 45–49. 17972874 10.1038/nature06197

[eva13116-bib-0071] Wang, Z. , Bafadhel, M. , Haldar, K. , Spivak, A. , Mayhew, D. , Miller, B. E. , Tal‐Singer, R. , Johnston, S. L. , Ramsheh, M. Y. , Barer, M. R. , Brightling, C. E. , & Brown, J. R. (2016). Lung microbiome dynamics in chronic obstructive pulmonary disease exacerbations. European Respiratory Journal, 47(4), 1082. 10.1183/13993003.01406-2015 26917613

[eva13116-bib-0072] Wilson, E. O. (1994). The Diversity of Life. Penguin.

[eva13116-bib-0073] Woodcock, S. , Van Der Gast, C. J. , Bell, T. , Lunn, M. , Curtis, T. P. , Head, I. M. , & Sloan, W. T. (2007). Neutral assembly of bacterial communities. FEMS Microbiology Ecology, 62, 171–180. 10.1111/j.1574-6941.2007.00379.x 17937674

[eva13116-bib-0074] Zhang, Q. G. , Buckling, A. , & Godfray, H. C. J. (2010). Quantifying the relative importance of niches and neutrality for coexistence in a model microbial system. Functional Ecology, 23(6), 1139–1147.

[eva13116-bib-0075] Zhang, Z. , Geng, J. , Tang, X. , Fan, H. , Xu, J. , Wen, X. , Ma, Z. , & Shi, P. (2014). Spatial heterogeneity and co‐occurrence patterns of human mucosal‐associated intestinal microbiota. The ISME Journal, 8(4), 881 10.1038/ismej.2013.185 24132077 PMC3960530

