# Supplementary material for: Niche‐neutral theoretic approach to mechanisms underlying the biodiversity and biogeography of human microbiomes
Source: Evol Appl. 2020 Oct 13;14(2):322–34. doi: 10.1111/eva.13116 (PMC7896709; doi:10.1111/eva.13116)
Supplement: Supplementary file 1 — Supplementary Material [file EVA-14-322-s001.pdf]

Online Supplementary Information (I) (OSI-1) for: Ma ZS (2020) Niche-neutral theoretic approach to the mechanisms underlying the biodiversity and biogeography of human microbiomes. *Evolutionary Applications*.

Table of Contents

Sections I-III are included in OSI-1 (this document), and Section IV in a separate OSI-2.

Section I. List of All Supplementary Tables

Section II. **Tables S1 & S2**: the two tables illustrate the fittings of MSN/NNH models with 24 selected metacommunities. The detailed fitting results for all 5420 samples representing 699 metacommunities and 5420 local communities were displayed in **Tables S3-S14**, which were provided in a separate file (OSI-2).

This section also includes detailed interpretations of the fitting results of the 24 selected metacommunities, which were generated from 4 possible fitting outcomes (MSN only, NNH only, both MSN & NNH, and neither MSN nor NNH) of 6 datasets (4x6=24).

Section III. **Table S15-S20**, exhibiting the results of Logistic Regression and Cox Regression modeling of the relationships among neutrality, niche differentiations, and metrics of community/species characteristics.

Section IV. **Table S3-S14** (*included* in OSI-2), exhibiting the detailed fitting results of MSN/NNH models to all the datasets analyzed in this study.

Section I. List of Supplementary Tables

Selected test results for the MSN model (OSI-1, this document)

Table S1. The selected test results of fitting Harris *et al's* (2015) HDP-MSN (hierarchical Dirichlet process, multi-site neutral) model to the six human microbiome datasets (including 699 meta-communities and 5420 local communities), excerpted from Tables S3-S8 in the OSI (*the column legends and their interpretations in the neutrality tests are noted below)

Selected test results for the NNH model (OSI-1, this document)

Table S2. The selected test results of fitting Tang & Zhou's (2013) NNH (niche-neutral hybrid) model to the six human microbiome datasets (including 699 meta-communities and 5420 local communities), excerpted from Tables S9-S14 in the OSI (the column legends are noted as*)

Detailed test results for the MSN model (Displayed in OSI-2)

Table S3. Test results of fitting MSN (multi-site neutral) model to HMP-oral datasets (*Sites*≥2, *Subjects*=146)

Table S4. Test results of fitting MSN (multi-site neutral) model to HMP-skin datasets (*Sites*≥2, *Subjects*=159)

Table S5. Test results of fitting MSN (multi-site neutral) model to HMP-vaginal datasets (*Sites*≥2, Subjects=72)

Table S6. Test results of fitting MSN (multi-site neutral) model to HMP-whole datasets (*Sites*≥5, Subjects=172)

Table S7. Test results of fitting MSN (multi-site neutral) model to Gut datasets (*Sites*=7, *Subjects*=11)

Table S8. Test results of fitting MSN (multi-site neutral) model to Lung datasets (*Sites*=4, *Subjects*=139)

Detailed test results for the NNH model (Displayed in OSI-2)

Table S9. Test results of fitting NNH (multi-site neutral) model to HMP-oral datasets (*Sites*≥2, *Subjects*=146)

Table S10. Test results of fitting NNH (multi-site neutral) model to HMP-skin datasets (*Sites*≥2, *Subjects*=159)

Table S11. Test results of fitting NNH (multi-site neutral) model to HMP-vaginal datasets (*Sites*≥2, Subjects=72)

Table S12. Test results of fitting NNH (multi-site neutral) model to HMP-whole datasets (*Sites*≥5, Subjects=172)

Table S13. Test results of fitting NNH (multi-site neutral) model to Gut datasets (*Sites*=7, *Subjects*=11)

Table S14. Test results of fitting NNH (multi-site neutral) model to Lung datasets (*Sites*=4, *Subjects*=139)

The factors influencing MSN/NNH model fittings (OSI-1, this document)

Table S15. Logistic regression analysis of the major factors influencing the neutrality (judged by passing the MSN model-fitting test) of the human microbiome at meta-community level

Table S16. Logistic regression analysis of major factors influencing the neutrality (judged by passing the MSN model-fitting test) of the human microbiome at local community level

Table S17. The classification table associated with the logistic regression (LR) analysis for the MSN model at the meta-community level and local community level, respectively

Table S18. Logistic regression analysis of major factors influencing the niche-neutral hybrid (NNH) performance (success) of the human microbiome at meta-community level

Table S19. The classification table associated with the logistic regression (LR) analysis for the NNH model at local community level (*i.e*., the LR model in Table 20).

Table S20. Cox regression analysis of major factors influencing the niche-neutral hybrid (NNH) performance (success) of the human microbiome at local community level

Section II. Tables S1-S2 and their interpretations

Tables S1 & S2 (below) selected the test results for maximally 24 selected metacommunity samples, excerpted from Tables S3-S8 (for MSN modeling) and Tables S9-S14 (for NNH modeling) in the OSI-2, Although both MSN and NNH tests were performed independently, the cases in the summary tables (Tables S1 & S2) were carefully selected to be representative of various combinations of the test results, *i.e.*, passing MSN only, passing NNH only, passing both MSN and NNH and passing neither, 4 possibilities for each of the six human microbiome datasets (a total of 4x6=24 cases or meta-communities). Therefore, Tables S1 and S2 offer windows to inspect the parameters and conclusions of testing the MSN/NNH models. To inspect the complete test results of the 699 meta-community samples, readers are referred to Tables S3-S14 in the OSI-2.

MSN (multisite neutral) modeling

With Harris *et al* (2017) multisite HDP-UNTB model (*i.e.*, MSN model), two-level tests (local community and meta-community levels) for the neutrality were performed. For both the tests, samples were generated from $\boldsymbol{N}$=2500 sets of fitted parameters, which were selected from every tenth iteration of the last 25,000 Gibbs samples (a total of 50,000 samples were simulated and the first 25,000 samples were discarded as burn-in). $\boldsymbol{N}=2500$ is chosen to compute the pseudo *p*-values for conducting the neutrality test. In addition, for each observed community sample, there is the *actual log-likelihood* L_0. Two additional parameters $\boldsymbol{\theta}$ and $\boldsymbol{M}$ are particular worthy of mentioning: θ is the median of the fundamental biodiversity parameters computed from 25,000 times of simulations, and M-value is the average of the medians of the migration rates of local communities in each meta-community, also computed from 25,000 times of simulations (note M is the average of medians per local community).

To test the neutrality at the meta-community level, assume $\boldsymbol{L_M}$ is the median of the log-likelihoods of the simulated neutral meta-community samples, and $\boldsymbol{N_M}$ is the number of simulated neutral meta-community samples, having their likelihoods satisfying $\boldsymbol{L \le L_0}$ (*i.e.,* the simulated likelihood not to exceed the actual likelihood) then the $\boldsymbol{P_M = N_M / N}$ is the pseudo *p*-value for testing the neutrality at meta-community level. If $\boldsymbol{P_M}$>0.05, the meta-community satisfies the MSN model.

To test the neutrality at the local community level, assume $\boldsymbol{L_L}$ is the median of the log-likelihoods of the simulated local community samples, and $\boldsymbol{N_L}$ is the number of simulated local community samples, having their likelihoods not exceeding the L_0, then $\boldsymbol{P_L=N_L/N}$, is the pseudo *p*-value for testing the neutrality at the local community level. If $\boldsymbol{P_L}>0.05$, the local community satisfies the neutral model. For detailed computational procedures and code to implement the MSN modeling, readers are referred to the original method paper by Harris et al (2017).

The full fitting results for the MSN model with 6 datasets are included in Table S3-S8 of the OSI-2 and following Table S1 (below, for selected samples from Table S3-S8).

NNH (niche-neutral hybrid) modeling

With Tang & Zhou (2013) NNH model, we computed and tabulated the fitting results in Table S9-S14 of the OSI-2 and Table S2 (below: for selected samples from Table S9-S14). The results include: the average number of individuals per niche (local community) in each metacommunity (J), the average species numbers per niche (local community) in each metacommunity (S), the average fundamental biodiversity parameter per niche (local community) in each metacommunity (θ), the average of the migration coefficients (m), the average of the birth to death ratio (x), the average of the migration rate ($\boldsymbol{\gamma}$). The last two columns in those Tables (S9-S14, Table S2) are the number and percentage of local communities (niches) that passed the local neutrality test.

The NNH model incorporated niche differentiations into Volkov *et al*. (2007) multi-site neutral model. Specifically, the per capita birth to death rates (x) and immigration parameter (γ) vary among species from different niches. In the case of our multi-site microbiome datasets, we treat each site as a niche occupied by a local microbial community and fit the neutral model for each local community. By first computing the number of species with abundance n in each local community, and then computing total expected number of species with abundance n in the metacommunity, one can obtain the theoretically expected number of species with each abundance level ($n=1, 2,\ldots$). After obtaining the theoretically predicted and observed species abundance distributions, the Chi-squared test is performed by comparing with the corresponding observed number of species for each corresponding abundance level (n). The *p*-value of Chi-squared test is then utilized to determine whether or not Tang & Zhou's (2013) hybrid model is suitable for a set of microbial communities sampled from the multiple microbiome sites of a

human individual. Specifically, at the metacommunity level, if *p*-value>0.05, then the meta-community satisfies the NNH and the metacommunity assembly is co-driven by both niche and neutral processes, which also implies that the meta-community itself does not satisfy the neutral theory, but within each niche, the local community is neutral. If *p*-value<0.05, the metacommunity does not satisfy the NNH, which also implies that within each niche, the local community is not neutral either, and the metacommunity assembly is solely influenced by the niche process. For detailed computational procedures and code to implement the NNH modeling, readers are referred to the original method paper by Tang & Zhou (2013).

References

Harris K, Parsons TL, Ijaz UZ, *et al*. (2017). Linking Statistical and Ecological Theory: Hubbell's Unified Neutral Theory of Biodiversity as a Hierarchical Dirichlet Process. *Proceedings of the IEEE*, 105(3):516-529.

Tang J, Zhou S. (2013). Hybrid niche-neutral models outperform an otherwise equivalent neutral model for fitting coral reef data. *Journal of Theoretical Biology*, 317(1):212-218.

Volkov I, Banavar JR, Hubbell SP, Maritan A. (2007). Patterns of relative species abundance in rainforests and coral reefs. *Nature* **450**:45–49.

Table S1. The selected test results of fitting Harris *et al's* (2017) HDP-MSN (hierarchical Dirichlet process, multi-site neutral) model to the six human microbiome datasets (including 699 meta-communities and 5420 local communities), excerpted from Tables S3-S8 in the OSI-2 (*the column legends and their interpretations in the neutrality tests are noted below)

Datasets	ID	L_O	θ	*M-value*	Metacommunity				Local Community			
					L_M	$\#N_M$	N	$**P_M$	L_L	$\#N_L$	N	$**P_L$
HMP (Oral)	160866180	-39276.994	2479.616	404.759	-39658.654	416	2500	0.834	-39656.731	286	2500	0.886
	246515023	-15522.687	2123.168	688.241	-14123.284	2500	2500	0.000	-14699.120	2500	2500	0.000
	178713055	-30835.242	2294.473	478.897	-31605.086	57	2500	0.977	-31801.049	0	2500	1.000
	256789458	-14889.466	2752.214	416.655	-14070.258	2499	2500	0.000	-14623.931	2220	2500	0.112
HMP (Skin)	132902142	-3150.748	536.140	138.368	-3216.947	716	2500	0.714	-3202.208	1083	2500	0.567
	160987560	-11503.150	1707.760	1629.485	-10680.152	2500	2500	0.000	-10852.340	2500	2500	0.000
	160967330	-10304.762	1002.092	2859.186	-10212.743	1697	2500	0.321	-9851.933	2499	2500	0.000
	875002022	-2877.205	817.475	169.219	-2594.960	2496	2500	0.002	-2730.332	2348	2500	0.061
HMP (Vaginal)	246515023	-1693.488	69.721	1661.087	-1853.194	226	2500	0.910	-1733.806	473	2500	0.811
	256789458	-2049.638	1544.165	45.299	-1855.302	2383	2498	0.046	-1921.186	2171	2498	0.131
	158114885	-1340.522	42.919	306.473	-1661.194	22	2500	0.991	-1425.677	107	2500	0.957
HMP (Total)	132902142	-38065.916	3559.293	370.025	-38355.535	600	2500	0.760	-38935.805	25	2500	0.990
	161351702	-8731.369	3850.748	443.719	-7173.746	2500	2500	0.000	-7672.465	2500	2500	0.000
	765398164	-4687.293	728.674	234.497	-4682.253	1287	2500	0.485	-4769.107	639	2500	0.744
	184349034	-36519.128	7099.860	319.325	-34765.011	2500	2500	0.000	-36136.941	2090	2500	0.164
Gut	S407	-6527.371	225.626	1610.896	-6902.900	140	2500	0.944	-6500.625	1511	2500	0.396
	S400	-16579.634	793.343	471.258	-17899.558	0	2500	1.000	-16962.178	100	2500	0.960
Lung	A3	-831.167	32.854	59.980	-985.861	139	2500	0.944	-857.579	795	2500	0.682
	A1	-481.585	13.308	32.427	-633.426	139	2500	0.944	-500.514	791	2500	0.684

*N=2500 is the number of Gibb samples selected from 25000 simulated communities (*i.e.,* every tenth iteration of the last 25,000 Gibbs samples), it is chosen to compute the pseudo p-value (as explained below) for conducting the neutrality test. $\boldsymbol{L_0}$ is the *actual log-likelihood*. θ is the median of biodiversity parameters computed from 25,000 times of simulations. M-value is the average medians of the migration rates of local communities in each meta-community, also computed from 25,000 times of simulations. $\boldsymbol{L_M}$ is the median of the log-likelihoods of the simulated neutral meta-community samples; and $\boldsymbol{N_M}$ is the number of simulated neutral meta-community samples with their likelihoods not exceeding the actual likelihood satisfying $\boldsymbol{L \le L_0}$ (where $\boldsymbol{L}$ and $\boldsymbol{L_0}$ are the simulated and actual likelihood respectively), $\boldsymbol{P_M = N_M / N}$ is the pseudo p-value for testing the neutrality at meta-community level; if $\boldsymbol{P_M}>0.05$, the meta-community satisfies the MSN model. $\boldsymbol{L_L}$ is the median of the log-likelihoods of the simulated local community samples, and $\boldsymbol{N_L}$ is the number of simulated local community samples with their likelihoods not exceeding the L_0, *i.e.*, $\boldsymbol{L \le L_0}$ (where $\boldsymbol{L}$ and $\boldsymbol{L_0}$ are the simulated and actual likelihood respectively), $\boldsymbol{P_L = N_L / N}$, is the pseudo p-value for testing the neutrality at the local community level; if $\boldsymbol{P_L}>0.05$, the local community satisfies the neutral model. See Fig 2 for an example of fitting the MSN model.

**Due to the typo/error in Harris *et al.* (2017), the $\boldsymbol{P_M}$-values exhibited here are adjusted as ($\boldsymbol{P_M}=1-\boldsymbol{P_{MS}}$), where $\boldsymbol{P_{MS}}$ is output from their computational program. Similarly, the $\boldsymbol{P_L}$-values are adjusted as ($\boldsymbol{P_L}=1-\boldsymbol{P_{LS}}$), where $\boldsymbol{P_{LS}}$ is output from their computational program.

#The $\boldsymbol{N_M}$ and $\boldsymbol{N_L}$ reported here are from the output of Harris et al. (2017) software. When computing the P-value, their "complements" (2500−N_M) or (2500−N_L) should be used to circumvent their error.

Table S2. The selected test results of fitting Tang & Zhou's (2013) NNH (niche-neutral hybrid) model to the six human microbiome datasets (including 699 meta-communities and 5420 local communities), excerpted from Tables S9-S14 in the OSI-2 (the column legends are noted as*)

	ID	*J*	*S*	*θ*	*m*	*x*	γ	R^2	χ^2	*p*-value	N^{pass}	$\%^{(pass)}$
HMP (Oral)	160866180	1652.063	522.188	907.849	0.001	0.372	1.008	1.000	53.076	0.000	6	37.5
	246515023	700.222	402.667	1061.209	0.001	0.293	0.994	1.000	0.676	0.984	1	11.1
	178713055	6283.571	949.143	981.375	0.000	0.491	1.003	1.000	7.964	0.632	1	14.3
	256789458	3455.750	782.000	1053.043	0.000	0.418	1.019	0.999	57.079	0.000	3	75.0
HMP (Skin)	132902142	1694.000	246.333	430.888	0.002	0.317	1.082	0.997	341.272	0.000	3	100.0
	160987560	1794.500	804.500	1591.265	0.001	0.326	0.996	1.000	2.577	0.765	0	0.0
	160967330	2096.750	834.250	1313.915	0.001	0.392	0.993	1.000	4.466	0.614	0	0.0
	875002022	1474.000	317.500	492.451	0.001	0.378	1.058	0.987	64.998	0.000	2	100.0
HMP (Vaginal)	246515023	2108.667	210.333	108.122	0.000	0.684	0.954	0.953	41.415	0.000	3	100.0
	256789458	5387.000	350.000	247.265	0.000	0.593	0.986	0.990	6.426	0.600	0	0.0
	158114885	3985.500	127.000	54.974	0.000	0.724	0.954	0.970	6.947	0.730	0	0.0
HMP (Total)	132902142	4662.500	749.600	841.491	0.001	0.434	1.027	0.999	87.094	0.000	7	70.0
	161351702	1445.000	587.667	939.129	0.001	0.399	0.988	1.000	7.266	0.297	0	0.0
	765398164	5593.500	526.000	456.615	0.000	0.539	1.008	0.999	9.215	0.418	0	0.0
	184349034	4416.250	793.625	1019.770	0.000	0.434	1.020	0.999	118.340	0.000	5	62.5
Gut	S407	1223.000	332.286	299.728	0.001	0.543	0.967	0.993	38.469	0.000	3	42.9
	S400	3630.571	583.143	557.865	0.000	0.513	0.998	1.000	6.383	0.701	0	0.0
Lung	A3	1666	51.000	31.833	0.001	0.653	0.966	0.989	22.149	0.014	1	25.0
	A1	1666	27.000	9.935	0.000	0.856	0.738	0.959	5.077	0.828	0	0.0

J*: the average number of individuals per niche (local community) in each metacommunity, *S*: the average species numbers per niche (local community) in each metacommunity, *θ*: the average fundamental biodiversity parameter per niche (local community) in each metacommunity, *m*: the average of the migration coefficients, *x*: the average of the birth to death ratio, **γ: the average of the migration rate, R^2: the goodness-of-fitting index, χ^2-value: the χ^2-value of chi-squared test for observed value against predicted value, *p*-value for the χ^2-test; when *p*-value > 0.05, the metacommunity satisfies the NNH model. The last two columns are the number and percentage of local communities (niches) that passed the local neutrality test. Note that $R^2=1$ resulted from approximation with four effective digits only (*e.g.*, 0.99995, exact 1 is nearly impossible to achieve). See Fig 3 for an example of fitting the NNH model.

Section III. Supplementary Tables S15-S20

Table S15. Logistic regression (LR) analysis of the major factors influencing the neutrality (judged by passing the MSN model-fitting test) of the human microbiome at meta-community level#

Variable (Factor)	LR Coefficient (B or Beta)	Standard Error	*p*-value of Wald Test	Odds Ratio: Exp (B)
**Community Dominance**	0.02892	0.00389	*<0.0001	1.02935
**Hill numbers (*q*=0): Species Richness**	0.00380	0.00051	*<0.0001	1.00381
**θ (Fundamental Biodiversity Number)**	-0.00131	0.00017	*<0.0001	0.99869
**Species Dominance**	-0.00717	0.00139	*<0.0001	0.99285

#Reference Group *PM*=0, *i.e.*, not passing neutrality test at meta-community level. R^2=0.33; *p-value=0.001

Table S16. Logistic regression (LR) analysis of major factors influencing the neutrality (judged by passing the MSN model-fitting test) of the human microbiome at local community level#

Variable (Factor)	LR Coefficient (B or Beta)	Standard Error	*p*-value of Wald Test	Odds Ratio: Exp (B)
**Community Dominance**	0.08010	0.00955	*<0.0001	1.08340
**Species Dominance**	-0.02221	0.00572	*<0.0001	0.97804
**Immigration Ratio (M-Value)**	-0.00097	0.00022	*<0.0001	0.99903
**θ (Fundamental Biodiversity Number)**	-0.00085	0.00017	*<0.0001	0.99915

#Reference Group *PL*=0, *i.e.*, not passing neutrality test at local community level. R^2=0.53; *p-value=0.001

Table S17. The classification table associated with the logistic regression (LR) analysis for the MSN model at the meta-community level and local community level, respectively

	**Estimated (for Meta-community)**		
**Actual**	0 (non-neutral)	1 (neutral)	Total
0 (non-neutral)	33	46	79
1 (neutral)	5	615	620
Total	38	661	699
**Percentage Correctly classified = 91.6%; Area Under ROC Curve=0.89**			
	**Estimated (for Local-community)**		
**Actual**	0 (non-neutral)	1 (neutral)	Total
0 (non-neutral)	34	20	54
1 (neutral)	6	639	645
Total	40	659	699
**Percentage Correctly classified = 96.4%; Area Under ROC Curve=0.93**			

Table S18. Logistic regression analysis of major factors influencing the niche-neutral hybrid (NNH) performance (success) of the human microbiome at meta-community level#

Variable (Factor)	LR Coefficient (B or Beta)	Standard Error	*p*-value of Wald Test	Odds Ratio: Exp (B)
**Community Dominance**	-0.01614	0.00367	***=0.0001	0.98399
X*=Ratio of Birth to Death *(b/d)	5.70576	1.10415	***<0.0001	300.594
***Y*= Migration of Each Niche**	-1.07973	0.63226	*p*=0.08769	0.33969
**Neutrality of Local Community (PL=1)**	4.22352	0.35051	***<0.0001	0.93220

#The model for *PM*=0, not passing NNH at the meta-community level. R^2=0.57; ****p*-value=0.001

Table S19. The classification table associated with the logistic regression (LR) analysis for the NNH model at local community level (*i.e.*, the LR model in Table 20).

	**Estimated**		
**Actual**	0 (non-neutral)	1 (neutral)	Total
0 (non-neutral)	535	23	558
1 (neutral)	33	104	137
Total	568	127	695
**Percentage Correctly classified = 91.9%**			
**Area Under ROC Curve=0.937**			

Table S20. Cox regression analysis of major factors influencing the niche-neutral hybrid (NNH) performance (success) of the human microbiome at local community level

Variable (Factor)	LR Coefficient (B or Beta)	Standard Error	*p*-value of Wald Test	Odds Ratio: Exp (B)
**Community Dominance**	0.001210	0.000248	***<0.0001	1.0012
X*=Ratio of Birth to Death *(b/d)	4.847869	0.619410	***<0.0001	127.4685
***Y*= Migration of Each Niche**	-1.864535	1.204772	*p*=0.1217	0.1550
**M=Migration rate Between Niches**	179.376135	66.538569	***0.0070	10000+
**Hill Numbers at q=0 (Species Richness)**	-0.001993	0.000223	***<0.0001	0.9980
**Hill Numbers at q=1 (Shannon Index)**	0.012003	0.000855	***<0.0001	1.0121
**Passing NNH at Metacommunity (PM=1)**	0.854414	0.163204	***<0.0001	2.3500

#The model predicts the probability for local communities to pass the local neutrality test.
R^2=0.43; **p*-value=0.001
