# Supplementary material for: Niche‐neutral theoretic approach to mechanisms underlying the biodiversity and biogeography of human microbiomes
Source: Evol Appl. 2020 Oct 13;14(2):322–34. doi: 10.1111/eva.13116 (PMC7896709; doi:10.1111/eva.13116)
Supplement: Supplementary file 2 — Supplementary Material [file EVA-14-322-s002.pdf]

Online Supplementary Information (II) (OSI-2) for: Ma ZS (2020)
Niche-neutral theoretic approach to the mechanisms underlying the biodiversity and biogeography of human microbiomes. *Evolutionary Applications*.

Section IV (**Table S3-S14**), exhibiting the detailed fitting results of MSN/NNH models to all the datasets analyzed in this study (*see* Table 1 for a brief introduction on the datasets). The fitting results of 24 samples selected from Tables S3-S14 were illustrated in Tables S1-S2 in the separate OSI-1.

Detailed test results for the MSN model

Table S3. Test results of fitting MSN (multi-site neutral) model to HMP-oral datasets (*Sites*≥2, *Subjects*=146)

Table S4. Test results of fitting MSN (multi-site neutral) model to HMP-skin datasets (*Sites*≥2, *Subjects*=159)

Table S5. Test results of fitting MSN (multi-site neutral) model to HMP-vaginal datasets (*Sites*≥2, Subjects=72)

Table S6. Test results of fitting MSN (multi-site neutral) model to HMP-whole datasets (*Sites*≥5, Subjects=172)

Table S7. Test results of fitting MSN (multi-site neutral) model to Gut datasets (*Sites*=7, *Subjects*=11)

Table S8. Test results of fitting MSN (multi-site neutral) model to Lung datasets (*Sites*=4, *Subjects*=139)

Detailed test results for the NNS model

Table S9. Test results of fitting NNH (multi-site neutral) model to HMP-oral datasets (*Sites*≥2, *Subjects*=146)

Table S10. Test results of fitting NNH (multi-site neutral) model to HMP-skin datasets (*Sites*≥2, *Subjects*=159)

Table S11. Test results of fitting NNH (multi-site neutral) model to HMP-vaginal datasets (*Sites*≥2, Subjects=72)

Table S12. Test results of fitting NNH (multi-site neutral) model to HMP-whole datasets (*Sites*≥5, Subjects=172)

Table S13. Test results of fitting NNH (multi-site neutral) model to Gut datasets (*Sites*=7, *Subjects*=11)

Table S14. Test results of fitting NNH (multi-site neutral) model to Lung datasets (*Sites*=4, *Subjects*=139)

Section IV. Supplementary Tables S3-S14

Table S3. Test results of fitting MSN (multi-site neutral) model to HMP-oral datasets (*Sites*≥2, *Subjects*=146)*

ID	L_O	θ	*M-value*	Metacommunity				Local community			
				L_M	$\#N_M$	N	$**P_M$	L_L	N_L	N	$**P_L$
132902142	-28149.896	2154.925	714.867	-29045.219	16	2500	0.994	-28573.583	195	2500	0.922
147406386	-39629.653	2660.637	408.021	-40532.103	64	2500	0.974	-41007.345	0	2500	1.000
158013734	-29836.678	1924.672	733.253	-30971.008	2	2500	0.999	-30065.367	490	2500	0.804
158114885	-34831.156	1654.219	1282.435	-37488.502	0	2500	1.000	-34572.595	2005	2500	0.198
158155345	-30249.865	1612.426	661.127	-32466.466	0	2500	1.000	-30676.119	201	2500	0.920
158216035	-40599.565	2180.221	632.669	-42970.687	0	2500	1.000	-41892.541	0	2500	1.000
158236265	-33222.074	1940.434	742.224	-35987.968	0	2500	1.000	-34113.351	5	2500	0.998
158276726	-36118.692	2518.069	396.629	-38031.991	0	2500	1.000	-37524.500	0	2500	1.000
158398106	-64369.255	1881.980	468.023	-72032.256	0	2500	1.000	-67226.641	0	2500	1.000
158418336	-76708.868	2678.051	689.142	-85287.837	0	2500	1.000	-79641.220	0	2500	1.000
158438567	-96967.545	3138.273	501.436	-103507.964	0	2500	1.000	-102087.984	0	2500	1.000
158458797	-86946.748	3201.282	481.204	-92073.605	0	2500	1.000	-90770.075	0	2500	1.000
158479027	-40598.315	2402.008	784.956	-42041.313	0	2500	1.000	-41267.532	57	2500	0.977
158499257	-37599.734	2899.482	473.448	-38495.503	39	2500	0.984	-38654.841	2	2500	0.999
158721788	-37344.407	2255.420	541.525	-40336.889	0	2500	1.000	-38911.847	0	2500	1.000
158742018	-83937.462	2812.451	592.332	-88521.512	0	2500	1.000	-86874.338	0	2500	1.000
158802708	-44972.417	2495.478	697.732	-48880.781	0	2500	1.000	-47044.796	0	2500	1.000
158822939	-68961.361	2961.117	1055.100	-74845.022	0	2500	1.000	-71917.934	0	2500	1.000
158883629	-92474.954	2815.965	560.528	-99957.468	0	2500	1.000	-97617.769	0	2500	1.000
158924089	-78510.607	3724.421	1115.656	-84619.143	0	2500	1.000	-81938.407	0	2500	1.000
158944319	-44189.819	2478.016	538.149	-48370.147	0	2500	1.000	-46713.827	0	2500	1.000
158964549	-101198.287	2943.816	619.967	-110925.949	0	2500	1.000	-106722.521	0	2500	1.000
158984779	-23664.717	1740.129	296.026	-24338.317	75	2500	0.970	-24723.659	0	2500	1.000
159005010	-63044.191	2896.621	1002.206	-67414.535	0	2500	1.000	-65596.404	0	2500	1.000
159025240	-32714.209	1598.890	770.591	-38791.981	0	2500	1.000	-34053.086	0	2500	1.000
159085930	-58861.096	3267.709	721.753	-61403.318	0	2500	1.000	-61357.170	0	2500	1.000
159146620	-114292.375	2857.826	828.231	-129677.924	0	2500	1.000	-120234.155	0	2500	1.000
159166850	-122189.810	2951.197	769.017	-135960.535	0	2455	1.000	-129700.872	0	2455	1.000
159227541	-92281.907	2695.204	779.381	-100452.452	0	2500	1.000	-95917.367	0	2500	1.000
159247771	-46964.694	2446.586	838.603	-50637.709	0	2500	1.000	-48610.887	0	2500	1.000
159389382	-85209.559	2921.579	584.460	-91672.529	0	2500	1.000	-88566.797	0	2500	1.000
159470302	-77301.160	2799.847	1383.139	-87476.135	0	2500	1.000	-80566.496	0	2500	1.000
159713063	-68990.500	3114.676	782.284	-73723.063	0	2500	1.000	-72785.354	0	2500	1.000
159733294	-68510.161	3058.656	1009.953	-75249.352	0	2500	1.000	-71802.233	0	2500	1.000
160744799	-19692.656	1516.039	511.685	-20846.834	0	2500	1.000	-19947.773	362	2500	0.855
160825720	-44761.302	2415.848	450.315	-45713.034	28	2500	0.989	-45111.250	368	2500	0.853
160866180	-39276.994	2479.616	404.759	-39658.654	416	2500	0.834	-39656.731	286	2500	0.886
160886410	-20864.909	1803.579	536.542	-20889.961	1135	2500	0.546	-20789.910	1571	2500	0.372
160906640	-25613.242	2185.247	505.423	-26050.681	204	2500	0.918	-25828.132	512	2500	0.795
160947100	-52522.659	2350.377	646.753	-54341.709	0	2500	1.000	-53094.423	140	2500	0.944
160967330	-22858.824	2013.313	418.061	-22677.590	1824	2500	0.270	-22856.261	1262	2500	0.495
160987560	-23512.755	3175.765	379.950	-22531.724	2500	2500	0.000	-23205.460	2223	2500	0.111
161007791	-16763.748	1126.384	869.248	-17638.938	4	2500	0.998	-16541.701	2174	2500	0.130
161270782	-18744.255	2401.984	461.605	-18036.196	2493	2496	0.001	-18405.553	2370	2496	0.050

161311242	-14873.264	2099.743	430.476	-14213.362	2497	2500	0.001	-14578.354	2366	2500	0.054
161412393	-7176.719	1089.440	942.832	-6747.128	2499	2500	0.000	-6861.289	2492	2500	0.003
161473083	-27744.041	2905.184	349.483	-27026.288	2462	2500	0.015	-27795.194	1077	2500	0.569
161554003	-23593.300	1599.322	361.356	-24834.344	1	2500	1.000	-24209.574	28	2500	0.989
178713055	-30835.242	2294.473	478.897	-31605.086	57	2500	0.977	-31801.049	0	2500	1.000
184349034	-15949.216	4043.209	482.534	-14645.173	2500	2500	0.000	-15395.236	2479	2500	0.008
208027353	-39377.266	2404.514	643.093	-40755.050	2	2500	0.999	-40129.976	17	2500	0.993
246515023	-15522.687	2123.168	688.241	-14123.284	2500	2500	0.000	-14699.120	2500	2500	0.000
256789458	-14889.466	2752.214	416.655	-14070.258	2499	2500	0.000	-14623.931	2220	2500	0.112
275382046	-11957.964	2615.712	396.331	-11015.010	2500	2500	0.000	-11548.545	2446	2500	0.022
289996019	-81508.490	2846.668	379.084	-86396.617	0	2500	1.000	-86287.587	0	2500	1.000
295137534	-15636.249	1989.932	351.264	-14885.947	2498	2500	0.001	-15284.063	2418	2500	0.033
336497421	-23506.365	1919.272	384.847	-23915.894	214	2500	0.914	-23840.044	207	2500	0.917
368533040	-23399.397	2298.677	507.658	-22897.248	2428	2500	0.029	-23018.656	2394	2500	0.042
370027359	-27738.038	2052.838	361.698	-28313.765	122	2500	0.951	-28480.254	14	2500	0.994
370425937	-33077.081	1763.010	339.271	-35017.946	0	2500	1.000	-34301.331	0	2500	1.000
375450439	-72386.760	2614.357	660.009	-78613.350	0	2500	1.000	-74498.927	0	2500	1.000
404239096	-33676.191	2615.427	455.666	-33725.077	1113	2500	0.555	-34201.803	110	2500	0.956
414519462	-20981.311	1464.561	339.871	-22223.898	0	2500	1.000	-21637.821	11	2500	0.996
451588811	-18510.871	2276.320	372.591	-17971.987	2455	2500	0.018	-18379.525	1822	2500	0.271
465578759	-36466.713	2516.657	350.526	-38548.123	0	2500	1.000	-38649.629	0	2500	1.000
492786515	-23169.138	1497.255	370.579	-25027.007	0	2500	1.000	-24007.852	5	2500	0.998
508703490	-21506.127	1356.832	358.107	-22327.580	18	2500	0.993	-21941.927	97	2500	0.961
514014184	-5676.375	4655.542	214.537	-4734.582	2500	2500	0.000	-4942.818	2500	2500	0.000
516889361	-10291.446	6250.090	320.356	-9050.365	2500	2500	0.000	-9447.098	2500	2500	0.000
517810313	-32261.467	1652.011	473.197	-34592.240	0	2500	1.000	-33365.091	0	2500	1.000
553359145	-33397.426	2337.000	423.335	-34559.913	8	2500	0.997	-34687.147	2	2500	0.999
604812005	-16517.933	1954.149	524.509	-15784.677	2500	2500	0.000	-16054.942	2492	2500	0.003
612472597	-42207.078	2309.823	774.721	-44560.852	0	2500	1.000	-43177.345	6	2500	0.998
638754422	-25845.164	3345.741	409.663	-24668.795	2500	2500	0.000	-25506.466	2229	2500	0.108
643185023	-21157.198	2191.206	306.581	-21357.484	620	2498	0.752	-21754.785	31	2498	0.988
650853796	-22183.678	2386.368	545.351	-21875.833	2168	2500	0.133	-22146.954	1410	2500	0.436
658594300	-15197.119	2350.326	295.523	-14880.926	2249	2500	0.100	-15294.511	816	2500	0.674
663835652	-33143.491	2203.340	618.494	-35002.997	0	2500	1.000	-34073.345	2	2500	0.999
668248235	-38465.998	2351.006	399.602	-39977.172	1	2500	1.000	-40079.145	0	2500	1.000
682449369	-41650.435	2331.818	654.239	-42633.089	40	2500	0.984	-42521.993	11	2500	0.996
686765762	-42738.456	2105.034	404.896	-45130.563	0	2500	1.000	-43800.724	3	2500	0.999
737052003	-43866.765	2417.501	493.087	-46898.392	0	2500	1.000	-45844.116	0	2500	1.000
763395383	-39399.449	2191.054	643.652	-42358.184	0	2500	1.000	-40755.702	0	2500	1.000
763435843	-45121.530	2463.324	542.708	-48526.126	0	2500	1.000	-47489.305	0	2500	1.000
763456073	-35740.481	1619.820	643.238	-38815.270	0	2500	1.000	-36768.058	3	2500	0.999
763476303	-25254.611	2047.887	329.300	-25611.389	373	2500	0.851	-25885.694	45	2500	0.982
763516763	-56836.625	2395.623	315.747	-60062.303	0	2500	1.000	-59594.117	0	2500	1.000
763536994	-111957.794	2611.874	553.405	-122696.937	0	2500	1.000	-117505.832	0	2500	1.000
763557224	-23336.404	1681.502	290.813	-24062.709	48	2500	0.981	-24199.997	3	2500	0.999
763597684	-107114.466	2901.238	701.464	-117945.598	0	2500	1.000	-113476.724	0	2500	1.000
763638144	-66723.402	3130.507	500.377	-69516.446	0	2500	1.000	-68934.604	0	2500	1.000
763698834	-94807.647	2768.476	605.918	-103747.730	0	2500	1.000	-100830.196	0	2500	1.000
763719065	-56297.654	2216.897	429.894	-58898.635	0	2500	1.000	-57949.010	0	2500	1.000
763759525	-67098.219	3205.975	423.210	-70044.641	0	2500	1.000	-69389.027	0	2500	1.000
763820215	-57724.372	2502.718	327.113	-62374.237	0	2500	1.000	-60903.429	0	2500	1.000

763840445	-85633.980	2825.394	601.899	-91836.218	0	2500	1.000	-88813.960	0	2500	1.000
763860675	-76053.098	2738.367	471.634	-81575.815	0	2500	1.000	-79227.834	0	2500	1.000
763880905	-39181.102	2820.142	411.100	-40243.541	22	2500	0.991	-40560.141	0	2500	1.000
763901136	-62616.968	3356.069	587.197	-63710.788	49	2500	0.980	-63986.336	2	2500	0.999
763921366	-39959.683	2305.100	637.697	-41898.458	0	2500	1.000	-41150.244	0	2500	1.000
763982056	-75951.555	2381.576	483.364	-85733.975	0	2500	1.000	-80003.276	0	2500	1.000
764002286	-78499.644	2668.262	479.383	-83809.790	0	2500	1.000	-82972.875	0	2500	1.000
764042746	-55962.415	2906.437	422.878	-56836.251	136	2500	0.946	-57392.047	1	2500	1.000
764062976	-84185.210	3667.544	410.376	-87844.572	0	2500	1.000	-87743.638	0	2500	1.000
764083206	-97772.143	2590.519	685.989	-109339.413	0	2500	1.000	-102938.481	0	2500	1.000
764143897	-88929.363	2418.709	600.234	-96945.678	0	2500	1.000	-93240.958	0	2500	1.000
764184357	-71984.655	2548.266	561.822	-76443.011	0	2500	1.000	-74544.380	0	2500	1.000
764224817	-82802.486	2845.497	467.739	-88816.632	0	2500	1.000	-86772.631	0	2500	1.000
764245047	-30115.037	2133.894	321.696	-31523.028	1	2500	1.000	-31449.471	0	2500	1.000
764285508	-35556.089	1698.588	508.160	-37238.590	1	2500	1.000	-36834.436	0	2500	1.000
764305738	-94940.470	2738.573	602.927	-103685.790	0	2500	1.000	-99979.524	0	2500	1.000
764325968	-77851.926	3160.779	432.542	-81883.279	0	2500	1.000	-81281.997	0	2500	1.000
764346198	-55600.663	2728.536	382.635	-56779.817	36	2500	0.986	-57430.119	0	2500	1.000
764366428	-27566.433	2104.950	297.157	-28333.443	53	2500	0.979	-28666.743	1	2500	1.000
764447348	-51989.701	2175.039	296.418	-56819.344	0	2500	1.000	-54669.831	0	2500	1.000
764467579	-64230.025	3154.598	509.305	-65655.920	17	2500	0.993	-65999.103	1	2500	1.000
764487809	-70510.202	2665.743	404.451	-77359.632	0	2500	1.000	-74486.975	0	2500	1.000
764508039	-30634.818	2470.292	387.691	-31149.195	196	2500	0.922	-31489.167	8	2500	0.997
764588959	-33181.657	2051.867	391.032	-34350.954	5	2500	0.998	-34326.925	0	2500	1.000
764649650	-34469.111	2288.574	439.889	-35707.138	2	2500	0.999	-35493.976	4	2500	0.998
764669880	-78283.052	2793.590	696.304	-84501.472	0	2500	1.000	-81117.992	0	2500	1.000
764750800	-41566.386	2665.514	607.135	-42801.617	7	2500	0.997	-42591.998	1	2500	1.000
764811490	-45373.316	3059.276	456.365	-47242.350	0	2500	1.000	-47212.627	0	2500	1.000
764831721	-39625.249	2624.072	620.903	-41507.551	0	2500	1.000	-40733.464	1	2500	1.000
764872181	-32753.577	1615.143	566.134	-34674.172	0	2500	1.000	-33509.678	12	2500	0.995
764892411	-35055.514	2016.306	613.286	-37119.933	0	2500	1.000	-35790.707	20	2500	0.992
764953101	-39030.591	2709.597	537.037	-40357.081	3	2500	0.999	-40203.891	0	2500	1.000
765013792	-34740.131	2137.018	621.118	-36324.694	0	2500	1.000	-35587.884	10	2500	0.996
765034022	-35875.530	2232.081	592.538	-37124.424	2	2500	0.999	-36494.583	59	2500	0.976
765074482	-54405.493	2840.135	465.019	-55819.018	9	2500	0.996	-55776.585	3	2500	0.999
765094712	-52130.136	2018.932	514.281	-57934.142	0	2500	1.000	-54456.593	0	2500	1.000
765155402	-30256.250	1976.290	364.171	-30674.810	375	2500	0.850	-31047.354	21	2500	0.992
765195863	-29701.096	2202.275	430.005	-30283.849	118	2500	0.953	-30236.127	91	2500	0.964
765216093	-34114.185	2083.181	507.170	-35265.073	6	2500	0.998	-34748.246	42	2500	0.983
765256553	-35472.354	2195.862	705.776	-37153.494	0	2500	1.000	-36274.640	9	2500	0.996
765276783	-30945.212	1905.586	408.842	-32154.311	2	2500	0.999	-31976.359	1	2500	1.000
765317243	-24229.438	1615.710	459.991	-26143.108	0	2500	1.000	-24942.885	12	2500	0.995
765337473	-31999.184	2196.359	476.943	-33129.830	4	2500	0.998	-32736.934	27	2500	0.989
765377934	-20735.739	1764.779	493.479	-21403.695	22	2500	0.991	-21234.367	50	2500	0.980
765560005	-43318.411	2679.127	630.995	-45408.868	0	2500	1.000	-44631.456	0	2500	1.000
809635352	-22121.096	1837.866	385.579	-22452.483	319	2500	0.872	-22388.431	375	2500	0.850
863126187	-43724.829	2968.384	483.304	-45178.643	1	2500	1.000	-45218.738	0	2500	1.000
892969023	-17342.550	1637.791	691.468	-16846.391	2470	2500	0.012	-16890.183	2480	2500	0.008
937495960	-22416.226	1971.588	382.472	-22502.748	955	2500	0.618	-22607.623	536	2500	0.786
953045535	-36031.562	3294.860	366.865	-35839.563	1697	2500	0.321	-36581.243	157	2500	0.937
970836795	-27717.931	2278.030	471.689	-28291.527	106	2500	0.958	-28107.292	191	2500	0.924

Passing Rate	129 (88.4%)	135 (92.5%)

*N=2500 is the number of Gibb samples selected from 25000 simulated communities (*i.e.,* every tenth iteration of the last 25,000 Gibbs samples), it is chosen to compute the pseudo p-value (as explained below) for conducting the neutrality test. $\boldsymbol{L_0}$ is the *actual log-likelihood*. θ is the median of biodiversity parameters computed from 25,000 times of simulations. M-value is the average medians of the migration rates of local communities in each meta-community, also computed from 25,000 times of simulations. $\boldsymbol{L_M}$ is the median of the log-likelihoods of the simulated neutral meta-community samples; and $\boldsymbol{N_M}$ is the number of simulated neutral meta-community samples with their likelihoods not exceeding the actual likelihood satisfying $\boldsymbol{L \leq L_0}$ (where $\boldsymbol{L}$ and $\boldsymbol{L_0}$ are the simulated and actual likelihood respectively), $\boldsymbol{P_M=N_M/N}$ is the pseudo p-value for testing the neutrality at meta-community level; if $\boldsymbol{P_M}>0.05$, the meta-community satisfies the MSN model. $\boldsymbol{L_L}$ is the median of the log-likelihoods of the simulated local community samples, and $\boldsymbol{N_L}$ is the number of simulated local community samples with their likelihoods not exceeding the L_0, *i.e*., $\boldsymbol{L \leq L_0}$ (where $\boldsymbol{L}$ and $\boldsymbol{L_0}$ are the simulated and actual likelihood respectively), $\boldsymbol{P_L=N_L/N}$, is the pseudo p-value for testing the neutrality at the local community level; if $\boldsymbol{P_L}>0.05$, the local community satisfies the neutral model. See Fig 2 for an example of fitting the MSN model.

**Due to the typo/error in Harris *et al*. (2017), the $\boldsymbol{P_M}$-values exhibited here are adjusted as ($\boldsymbol{P_M}=1-\boldsymbol{P_{MS}}$), where $\boldsymbol{P_{MS}}$ is output from their computational program. Similarly, the $\boldsymbol{P_L}$-values are adjusted as ($\boldsymbol{P_L}=1-\boldsymbol{P_{LS}}$), where $\boldsymbol{P_{LS}}$ is output from their computational program.

#The $\boldsymbol{N_M}$ and $\boldsymbol{N_L}$ reported here are from the output of Harris et al. (2017) software. When computing the P-value, their "complements" ($2500-N_M$) or ($2500-N_L$) should be used to circumvent their error.

Table S4. Test results of fitting MSN (multi-site neutral) model to HMP-skin datasets (*Sites*≥2, *Subjects*=159)*

ID	L_O	θ	*M-value*	*Metacommunity*				*Local community*			
				L_M	$\#N_M$	*N*	$**P_M$	L_L	$\#N_L$	*N*	$**P_L$
132902142	-3150.748	536.140	138.368	-3216.947	716	2500	0.714	-3202.208	1083	2500	0.567
147406386	-7817.265	609.296	414.002	-9609.946	0	2500	1.000	-8361.098	0	2500	1.000
158013734	-8579.881	918.487	322.799	-9322.092	0	2500	1.000	-8937.994	58	2500	0.977
158114885	-15328.765	2172.028	444.152	-15445.276	805	2500	0.678	-15653.011	184	2500	0.926
158155345	-14765.667	2229.517	271.131	-14830.981	998	2500	0.601	-15315.301	23	2500	0.991
158216035	-13878.884	2515.791	417.510	-13609.503	2138	2500	0.145	-14075.009	456	2500	0.818
158236265	-9340.662	1997.491	270.135	-8926.513	2435	2500	0.026	-9321.278	1340	2500	0.464
158276726	-7677.723	985.176	208.387	-8004.865	102	2500	0.959	-8056.610	33	2500	0.987
158398106	-12787.830	901.454	2291.023	-14995.177	0	2500	1.000	-12673.323	1911	2500	0.236
158418336	-20390.865	1421.262	1999.725	-23165.676	0	2500	1.000	-20208.147	1981	2500	0.208
158438567	-8545.663	1618.594	331.304	-8102.873	2491	2500	0.004	-8320.037	2338	2500	0.065
158458797	-12088.773	2185.194	250.685	-12015.665	1563	2500	0.375	-12315.639	368	2500	0.853
158479027	-4415.166	580.143	1326.766	-4428.803	1131	2500	0.548	-4095.360	2498	2500	0.001
158721788	-5831.145	853.538	3042.935	-5565.128	2454	2500	0.018	-5420.018	2500	2500	0.000
158742018	-18754.937	1869.372	542.474	-20476.187	0	2500	1.000	-19393.327	7	2500	0.997
158802708	-24159.491	4652.512	511.050	-23166.498	2493	2500	0.003	-24061.343	1576	2500	0.370
158822939	-23661.584	2269.718	2885.786	-25436.723	0	2500	1.000	-23439.901	2000	2500	0.200
158883629	-16670.391	1485.507	711.618	-18770.834	0	2500	1.000	-17227.546	11	2500	0.996
158924089	-18421.644	1434.284	2065.599	-20877.968	0	2500	1.000	-18527.705	764	2500	0.694
158944319	-5311.167	697.169	5214.276	-5244.092	1751	2500	0.300	-4972.622	2500	2500	0.000
158964549	-21347.919	2653.796	260.264	-21300.591	1400	2500	0.440	-21714.054	236	2500	0.906
159005010	-6046.649	3617.823	89.004	-5374.200	2500	2500	0.000	-5595.182	2489	2500	0.004
159025240	-6306.527	905.256	1993.273	-6057.888	2408	2497	0.036	-5856.924	2497	2497	0.000
159085930	-12707.305	2732.980	229.411	-12144.826	2471	2500	0.012	-12661.420	1446	2500	0.422
159146620	-21796.169	2188.486	642.178	-24030.104	0	2500	1.000	-22554.899	5	2500	0.998
159166850	-53549.350	2044.105	3069.945	-66717.565	0	2500	1.000	-54569.666	4	2500	0.998
159227541	-18282.420	1948.198	588.045	-19018.914	11	2500	0.996	-18785.868	26	2500	0.990
159247771	-8365.786	1410.548	194.588	-8228.113	1928	2500	0.229	-8520.295	486	2500	0.806
159389382	-13891.490	1093.902	987.027	-16054.213	0	2500	1.000	-14251.106	88	2500	0.965
159470302	-24882.886	4255.843	503.894	-25434.090	144	2500	0.942	-26049.085	1	2500	1.000
159713063	-24472.321	1863.612	1337.203	-27738.517	0	2500	1.000	-25495.884	0	2500	1.000
159733294	-17115.617	2099.169	469.534	-18376.600	0	2500	1.000	-18069.379	0	2500	1.000
160744799	-2633.176	2473.084	76.182	-2349.760	2469	2498	0.012	-2442.813	2364	2498	0.054
160825720	-18982.093	2251.520	879.480	-18459.554	2471	2498	0.011	-18651.800	2400	2498	0.039
160866180	-11184.014	2282.764	310.724	-10454.138	2500	2500	0.000	-10921.722	2285	2500	0.086
160886410	-7213.756	1077.513	915.365	-7021.499	2236	2500	0.106	-6903.096	2477	2500	0.009
160906640	-6394.957	1024.170	3176.388	-6141.311	2425	2500	0.030	-5961.675	2500	2500	0.000
160947100	-14146.419	2607.071	598.390	-13020.005	2499	2500	0.000	-13556.393	2499	2500	0.000
160967330	-10304.762	1002.092	2859.186	-10212.743	1697	2500	0.321	-9851.933	2499	2500	0.000
160987560	-11503.150	1707.760	1629.485	-10680.152	2500	2500	0.000	-10852.340	2500	2500	0.000
161007791	-10516.723	1459.141	934.300	-9801.460	2500	2500	0.000	-10008.088	2500	2500	0.000
161270782	-10525.211	1380.380	1399.554	-10086.222	2486	2500	0.006	-10037.588	2500	2500	0.000
161311242	-9010.558	904.009	1499.163	-9024.971	1151	2500	0.540	-8673.652	2479	2500	0.008
161331472	-3542.948	645.032	1730.440	-3182.381	2500	2500	0.000	-3281.389	2500	2500	0.000
161412393	-12216.948	947.932	2133.271	-14684.709	0	2500	1.000	-12416.025	292	2500	0.883
161473083	-4079.363	751.623	167.613	-4012.533	1770	2500	0.292	-4081.449	1228	2500	0.509

161554003	-8986.362	2119.088	316.794	-8446.903	2492	2500	0.003	-8811.066	2163	2500	0.135
178713055	-3303.050	1101.148	129.142	-3060.405	2462	2500	0.015	-3214.551	1972	2500	0.211
184349034	-10867.765	1135.774	462.728	-11721.899	0	2500	1.000	-11227.166	67	2500	0.973
208027353	-8486.561	973.561	278.383	-9137.688	1	2500	1.000	-8808.143	64	2500	0.974
246515023	-13061.891	1326.694	1473.339	-14250.315	0	2500	1.000	-12936.757	1902	2500	0.239
256789458	-8379.605	792.748	657.397	-9675.870	0	2500	1.000	-8581.707	262	2500	0.895
275382046	-5672.255	1108.766	150.122	-5756.999	762	2500	0.695	-5966.841	67	2500	0.973
295137534	-6596.496	1501.996	240.886	-6280.939	2426	2500	0.030	-6517.855	1751	2500	0.300
336497421	-10755.248	1628.923	1991.210	-10279.149	2484	2500	0.006	-10291.267	2499	2500	0.000
338793263	-6836.932	725.555	813.375	-7682.389	0	2500	1.000	-6941.470	535	2500	0.786
355657046	-8614.769	665.277	1519.789	-9658.084	0	2500	1.000	-8457.538	2193	2500	0.123
368533040	-11977.845	1045.352	1325.337	-13468.230	0	2500	1.000	-11946.276	1426	2500	0.430
370027359	-12904.096	678.241	2355.370	-15322.959	0	2500	1.000	-12836.049	1613	2500	0.355
370425937	-12412.673	1064.496	1896.095	-14348.036	0	2500	1.000	-12439.706	1103	2500	0.559
375450439	-7495.728	955.545	378.642	-7945.748	21	2500	0.992	-7551.431	910	2500	0.636
404239096	-4844.262	485.908	3440.926	-5249.337	8	2500	0.997	-4647.237	2458	2500	0.017
414519462	-25841.966	2981.572	791.267	-26708.365	11	2500	0.996	-26395.386	57	2500	0.977
451588811	-11006.941	1066.448	3900.744	-11793.663	0	2500	1.000	-10679.396	2462	2500	0.015
465578759	-8165.256	817.446	627.527	-9179.088	0	2500	1.000	-8214.501	958	2500	0.617
492786515	-8218.672	794.047	760.816	-9127.002	0	2500	1.000	-8216.460	1263	2500	0.495
508703490	-4854.902	543.421	1101.785	-5325.347	4	2500	0.998	-4786.819	1853	2500	0.259
516889361	-10308.518	1884.622	493.032	-9920.277	2456	2500	0.018	-10158.071	2063	2500	0.175
517810313	-12988.735	986.151	961.207	-15799.483	0	2500	1.000	-13621.700	0	2500	1.000
550534656	-12399.021	1400.120	478.725	-13476.524	0	2500	1.000	-12949.968	7	2500	0.997
553359145	-9354.714	850.295	624.016	-10561.150	0	2500	1.000	-9493.522	501	2500	0.800
604812005	-6443.223	819.636	2833.046	-6191.813	2420	2500	0.032	-6081.463	2499	2500	0.000
612472597	-7517.612	816.459	836.247	-7717.288	333	2500	0.867	-7343.668	2209	2500	0.116
638754422	-10009.954	912.817	1132.502	-11480.960	0	2500	1.000	-10029.135	1116	2500	0.554
643185023	-1250.623	290.414	25.053	-1216.688	1661	2500	0.336	-1269.238	1013	2500	0.595
650853796	-8790.728	1792.361	434.648	-8457.114	2420	2500	0.032	-8734.898	1574	2500	0.370
658594300	-2164.170	1319.044	61.859	-1907.982	2486	2500	0.006	-2003.911	2365	2500	0.054
668248235	-15736.627	1586.611	771.247	-17482.504	0	2500	1.000	-16136.448	87	2500	0.965
675950834	-6123.076	757.745	1535.467	-6058.968	1668	2500	0.333	-5810.717	2491	2500	0.004
682102541	-6684.985	768.708	330.965	-7756.768	0	2500	1.000	-7177.681	1	2500	1.000
682449369	-5417.223	923.288	166.887	-5405.194	1330	2500	0.468	-5478.521	815	2500	0.674
686765762	-29772.170	2696.289	589.318	-30089.293	459	2500	0.816	-30330.676	65	2500	0.974
706846339	-5849.917	766.121	2521.035	-5790.623	1685	2500	0.326	-5536.917	2491	2500	0.004
737052003	-5410.714	641.375	1049.867	-5640.904	150	2500	0.940	-5366.758	1672	2500	0.331
739574095	-3015.022	394.654	389.209	-3392.594	0	2500	1.000	-3098.111	450	2500	0.820
763395383	-9832.191	901.044	1453.609	-10425.454	5	2500	0.998	-9661.554	2166	2500	0.134
763435843	-6473.137	694.300	927.452	-6734.881	166	2498	0.934	-6377.088	2040	2498	0.183
763456073	-3552.537	504.164	1159.975	-3682.488	280	2500	0.888	-3469.632	2086	2500	0.166
763476303	-10340.747	1058.620	236.438	-11635.540	0	2500	1.000	-11330.404	0	2500	1.000
763516763	-16432.356	1329.472	688.485	-18241.436	0	2500	1.000	-16897.474	94	2500	0.962
763536994	-32642.285	1829.577	492.773	-40547.463	0	2500	1.000	-35682.094	0	2500	1.000
763597684	-40734.753	2317.569	1311.300	-49524.527	0	2500	1.000	-43658.460	0	2500	1.000
763638144	-2194.458	484.504	58.947	-2142.694	1735	2500	0.306	-2223.510	976	2500	0.610
763678604	-23417.894	1219.138	449.452	-29274.734	0	2500	1.000	-25493.672	0	2500	1.000
763698834	-35909.549	1646.739	784.180	-45506.112	0	2500	1.000	-38947.882	0	2500	1.000
763719065	-6401.659	934.070	69.299	-6828.120	38	2500	0.985	-6867.425	6	2500	0.998
763759525	-22013.092	2048.790	396.532	-24234.178	0	2500	1.000	-23396.862	0	2500	1.000

763820215	-6620.059	1787.667	107.248	-6382.886	2228	2500	0.109	-6664.319	1009	2500	0.596
763840445	-13185.098	1086.339	292.178	-14831.976	0	2500	1.000	-13810.306	11	2500	0.996
763860675	-15366.241	1999.912	255.787	-16222.171	3	2500	0.999	-16252.346	1	2500	1.000
763880905	-24840.396	1161.863	421.318	-32032.440	0	2500	1.000	-27369.612	0	2500	1.000
763901136	-11964.264	1344.512	92.247	-12835.092	2	2500	0.999	-12852.246	1	2500	1.000
763921366	-9861.581	1128.150	234.522	-10664.034	0	2500	1.000	-10455.168	3	2500	0.999
763982056	-21752.554	1468.495	607.084	-24726.348	0	2500	1.000	-23123.569	0	2500	1.000
764002286	-23387.433	1375.302	301.783	-28904.238	0	2500	1.000	-25877.890	0	2500	1.000
764042746	-5292.934	1797.560	96.365	-5106.600	2105	2500	0.158	-5313.328	1123	2500	0.551
764062976	-5773.869	1949.659	102.653	-5212.071	2499	2500	0.000	-5463.011	2435	2500	0.026
764083206	-18183.951	899.703	386.698	-23237.035	0	2500	1.000	-20004.967	0	2500	1.000
764143897	-9603.356	1278.429	163.781	-9623.284	1144	2500	0.542	-9831.080	233	2500	0.907
764184357	-9648.926	553.922	1391.301	-11598.240	0	2500	1.000	-9343.912	2455	2500	0.018
764224817	-14837.047	1063.624	975.328	-16274.702	0	2500	1.000	-14699.238	1848	2500	0.261
764245047	-4120.941	914.663	114.067	-3811.876	2485	2500	0.006	-4004.923	2069	2500	0.172
764305738	-53097.476	2764.209	1229.943	-67478.421	0	2500	1.000	-57555.148	0	2500	1.000
764325968	-16395.980	1165.907	905.927	-17913.672	0	2500	1.000	-16581.717	454	2500	0.818
764346198	-6174.475	442.055	1877.977	-7376.752	0	2500	1.000	-6151.399	1482	2500	0.407
764366428	-2455.265	304.184	491.351	-2487.381	913	2500	0.635	-2344.317	2330	2500	0.068
764447348	-9316.758	840.441	835.722	-10211.149	0	2500	1.000	-9264.813	1551	2500	0.380
764467579	-2444.361	598.831	51.832	-2423.358	1412	2500	0.435	-2508.666	690	2500	0.724
764487809	-12076.617	1054.751	463.704	-12692.550	5	2500	0.998	-12232.400	537	2500	0.785
764508039	-6957.775	608.878	1035.413	-7623.823	0	2500	1.000	-6649.959	2487	2500	0.005
764588959	-3002.299	435.855	209.051	-2951.663	1746	2500	0.302	-2916.469	2018	2500	0.193
764649650	-7589.192	794.948	562.067	-8075.340	6	2500	0.998	-7503.377	1749	2500	0.300
764669880	-13999.861	879.911	365.234	-15609.571	0	2500	1.000	-14429.404	39	2500	0.984
764710340	-4465.737	1010.283	91.803	-4187.036	2446	2500	0.022	-4376.137	1889	2500	0.244
764750800	-11410.183	1060.685	973.104	-12943.700	0	2500	1.000	-11863.081	12	2500	0.995
764811490	-7616.314	851.603	1002.424	-7913.462	130	2500	0.948	-7453.060	2206	2500	0.118
764831721	-5642.585	1485.345	224.914	-5219.937	2491	2500	0.004	-5470.351	2197	2500	0.121
764872181	-7263.425	705.504	470.157	-8936.193	0	2500	1.000	-7859.659	0	2500	1.000
764892411	-6080.588	490.028	1743.785	-6866.449	0	2500	1.000	-5917.760	2325	2500	0.070
765013792	-4251.613	680.545	210.502	-4166.790	1887	2500	0.245	-4268.606	1093	2500	0.563
765034022	-4469.483	666.214	167.042	-4554.957	585	2500	0.766	-4547.140	607	2500	0.757
765074482	-6866.157	485.955	2156.081	-8185.652	0	2500	1.000	-6793.454	1851	2500	0.260
765094712	-25051.296	1291.470	1713.916	-29144.634	0	2500	1.000	-24981.586	1530	2500	0.388
765135172	-4729.347	462.125	1240.223	-5079.471	20	2500	0.992	-4463.160	2482	2500	0.007
765155402	-16303.817	2227.539	342.495	-16321.552	1174	2500	0.530	-16555.642	320	2500	0.872
765195863	-10787.951	773.403	1296.697	-12133.092	0	2500	1.000	-10529.902	2335	2500	0.066
765216093	-11587.053	869.361	820.824	-13437.642	0	2500	1.000	-11639.510	987	2500	0.605
765256553	-2908.821	458.029	111.421	-3049.651	363	2500	0.855	-3101.123	105	2500	0.958
765276783	-11804.388	834.080	922.207	-14141.122	0	2500	1.000	-12089.725	138	2500	0.945
765317243	-4307.671	562.752	407.317	-4829.402	1	2500	1.000	-4498.107	167	2500	0.933
765337473	-11831.195	734.551	2244.245	-13937.611	0	2500	1.000	-11693.856	2015	2500	0.194
765377934	-15432.448	1053.525	1657.041	-17624.471	0	2500	1.000	-15223.985	2109	2500	0.156
765398164	-4687.293	728.674	234.497	-4682.253	1287	2500	0.485	-4769.107	639	2500	0.744
765519544	-12036.450	667.678	2499.293	-14380.031	0	2500	1.000	-11996.478	1513	2500	0.395
765539774	-7273.177	1218.710	237.910	-7154.431	1904	2500	0.238	-7385.910	588	2500	0.765
765560005	-21025.501	1399.441	702.412	-24335.773	0	2500	1.000	-21629.443	33	2500	0.987
765620695	-3935.003	1736.961	97.399	-3731.699	2257	2500	0.097	-3893.153	1528	2500	0.389
765640925	-6139.936	564.094	3294.446	-6908.787	0	2500	1.000	-6040.786	2041	2500	0.184

765661155	-4943.845	598.035	553.157	-5650.425	0	2500	1.000	-5128.814	159	2500	0.936
765701615	-14337.057	831.027	1850.574	-17306.163	0	2500	1.000	-14375.918	1047	2500	0.581
809635352	-25201.870	2489.273	1106.951	-26285.310	1	2500	1.000	-25656.035	110	2500	0.956
823052294	-7274.195	827.923	1416.260	-7472.839	258	2500	0.897	-6941.690	2489	2500	0.004
857980665	-8857.268	911.650	767.090	-9640.554	0	2500	1.000	-8930.071	826	2500	0.670
861967750	-11682.510	1487.437	375.029	-12029.517	132	2500	0.947	-11929.118	255	2500	0.898
863126187	-10636.054	1237.628	717.713	-11504.051	0	2500	1.000	-10878.327	227	2500	0.909
875002022	-2877.205	817.475	169.219	-2594.960	2496	2500	0.002	-2730.332	2348	2500	0.061
892969023	-11519.247	1316.999	537.369	-12510.517	0	2500	1.000	-11957.097	15	2500	0.994
953045535	-6465.513	589.910	514.806	-7383.993	0	2500	1.000	-6544.704	688	2500	0.725
970836795	-9271.413	1424.870	634.967	-9183.675	1682	2500	0.327	-9201.245	1677	2500	0.329
Passing Rate							130 (81.2%)				133 (83.6%)

*, ** See the bottom notes of Table S3 for the interpretations of the table columns here.

Table S5. Test results of fitting MSN (multi-site neutral) model to HMP-vaginal datasets (*Sites*≥2, Subjects=72)

ID	L_O	θ	*M-value*	Metacommunity				Local community			
				L_M	$\#N_M$	N	$**P_M$	L_L	$\#N_L$	N	$**P_L$
158013734	-1302.297	42.908	683.228	-1483.849	171	2500	0.932	-1295.027	1406	2500	0.438
158114885	-1340.522	42.919	306.473	-1661.194	22	2500	0.991	-1425.677	107	2500	0.957
158236265	-3236.625	176.229	279.927	-4162.524	0	2500	1.000	-3524.520	3	2500	0.999
158276726	-2317.813	123.150	654.893	-2796.953	0	2500	1.000	-2333.502	1018	2500	0.593
158458797	-6698.868	198.524	251.763	-7987.049	0	2500	1.000	-6957.434	91	2500	0.964
158742018	-7768.221	194.791	133.060	-8505.358	35	2500	0.986	-8229.358	3	2500	0.999
158883629	-6265.344	155.898	144.879	-8678.790	0	2500	1.000	-7008.514	0	2500	1.000
158944319	-3232.284	164.511	180.333	-4256.236	0	2500	1.000	-3643.200	0	2500	1.000
158984779	-3498.532	160.680	341.072	-4502.028	0	2500	1.000	-3803.717	3	2500	0.999
159005010	-7913.095	584.602	387.301	-9169.816	0	2500	1.000	-8540.991	0	2500	1.000
159025240	-3182.880	131.524	291.829	-3978.037	0	2500	1.000	-3449.992	7	2500	0.997
159085930	-6022.655	250.914	300.975	-7290.551	0	2500	1.000	-6669.464	0	2500	1.000
159227541	-5265.125	233.875	54.966	-6010.197	1	2500	1.000	-5855.320	1	2500	1.000
159247771	-2165.938	83.046	360.597	-2943.680	0	2500	1.000	-2332.232	40	2500	0.984
159470302	-5532.497	242.339	624.644	-7272.814	0	2500	1.000	-5979.443	0	2500	1.000
159713063	-3320.534	138.636	223.783	-4328.382	0	2500	1.000	-3680.435	1	2500	1.000
159733294	-4442.737	201.243	450.498	-5721.871	0	2500	1.000	-4744.457	15	2500	0.994
160744799	-2009.528	84.337	697.272	-2256.725	90	2500	0.964	-1888.881	2428	2500	0.029
160866180	-6563.704	183.157	865.200	-7485.075	3	2500	0.999	-6646.638	614	2500	0.754
161007791	-3339.215	236.041	651.998	-3579.030	111	2500	0.956	-3303.688	1662	2500	0.335
161473083	-2279.900	133.872	239.288	-2673.097	3	2500	0.999	-2321.985	770	2500	0.692
178713055	-1403.374	1010.353	28.472	-1275.504	2251	2500	0.100	-1324.747	1989	2500	0.204
208027353	-1428.269	71.699	731.253	-1746.632	6	2500	0.998	-1449.851	837	2500	0.665
246515023	-1693.488	69.721	1661.087	-1853.194	226	2500	0.910	-1733.806	473	2500	0.811
256789458	-2049.638	1544.165	45.299	-1855.302	2383	2498	0.046	-1921.186	2171	2498	0.131
370425937	-2117.736	95.130	261.821	-2488.695	6	2500	0.998	-2127.888	1100	2500	0.560
375450439	-8966.493	422.390	156.636	-10061.476	0	2500	1.000	-9675.056	1	2500	1.000
414519462	-1407.421	52.269	536.644	-1939.566	0	2500	1.000	-1477.990	246	2500	0.902
451588811	-5052.341	547.531	194.843	-5972.189	0	2500	1.000	-5547.559	1	2500	1.000
465578759	-2396.537	105.898	319.291	-3304.635	0	2500	1.000	-2559.802	58	2500	0.977
508703490	-1500.691	81.078	376.521	-1874.978	0	2499	1.000	-1529.438	742	2499	0.703
553359145	-1648.083	57.113	289.205	-2184.000	2	2500	0.999	-1777.219	50	2500	0.980
561079058	-1665.620	135.349	153.860	-2142.376	0	2500	1.000	-1840.335	21	2500	0.992
612472597	-3460.046	179.012	294.283	-4517.102	0	2500	1.000	-3788.362	1	2500	1.000
638754422	-3682.903	196.571	1362.837	-4462.811	0	2500	1.000	-3685.726	1216	2500	0.514
650853796	-1488.709	112.032	132.943	-1742.941	18	2500	0.993	-1538.533	633	2500	0.747
663835652	-3804.059	192.146	377.227	-4522.954	0	2500	1.000	-4068.753	6	2500	0.998
682449369	-2399.612	75.573	1635.972	-2731.022	54	2500	0.978	-2410.184	1084	2500	0.566
737052003	-3685.846	157.891	359.562	-4640.780	0	2500	1.000	-4003.749	4	2500	0.998
739574095	-3467.686	292.117	271.901	-4368.244	0	2500	1.000	-3822.146	2	2500	0.999
763456073	-2592.063	177.130	122.770	-3177.659	0	2500	1.000	-2799.940	68	2500	0.973
763557224	-2665.182	144.948	174.905	-3597.990	0	2500	1.000	-3011.367	0	2500	1.000
763638144	-4060.931	147.263	113.055	-4488.276	45	2500	0.982	-4330.448	20	2500	0.992
763698834	-10101.448	317.995	238.786	-14323.194	0	2500	1.000	-11819.109	0	2500	1.000
763719065	-6105.430	214.676	983.737	-6877.639	3	2500	0.999	-6230.008	403	2500	0.839
763759525	-9137.422	287.241	302.413	-11895.915	0	2500	1.000	-10028.610	0	2500	1.000
763820215	-4443.036	102.140	247.015	-6416.459	0	2500	1.000	-4801.372	3	2500	0.999

763840445	-5200.155	119.159	171.844	-6730.041	0	2500	1.000	-5570.708	11	2500	0.996
763901136	-7491.033	234.625	621.668	-8432.862	2	2500	0.999	-7662.805	296	2500	0.882
763921366	-2649.873	137.523	162.161	-3556.149	0	2500	1.000	-2986.336	0	2500	1.000
764042746	-5766.102	142.380	772.132	-6421.693	21	2500	0.992	-5749.925	1381	2500	0.448
764062976	-4090.741	103.929	195.858	-5693.570	0	2500	1.000	-4427.871	2	2500	0.999
764143897	-7447.356	147.415	763.064	-9952.291	0	2500	1.000	-7723.818	58	2500	0.977
764184357	-7255.100	168.594	488.279	-9593.773	0	2500	1.000	-7520.565	99	2500	0.960
764245047	-2322.955	110.590	176.716	-3208.985	0	2500	1.000	-2626.291	2	2500	0.999
764346198	-7466.850	272.612	459.222	-8987.287	0	2500	1.000	-7830.180	25	2500	0.990
764366428	-2221.938	88.888	354.721	-2954.930	0	2500	1.000	-2273.535	655	2500	0.738
764467579	-6733.235	363.113	249.255	-8407.611	0	2500	1.000	-7432.303	0	2500	1.000
764588959	-1152.735	49.222	643.255	-1364.673	57	2500	0.977	-1180.918	675	2500	0.730
764649650	-2932.899	174.598	271.755	-3770.417	0	2500	1.000	-3190.579	4	2500	0.998
764710340	-1445.355	47.990	540.710	-1932.207	0	2500	1.000	-1529.987	125	2500	0.950
764811490	-4535.511	198.551	775.217	-5757.163	0	2500	1.000	-4796.947	11	2500	0.996
765013792	-2670.199	151.778	229.888	-3495.489	0	2500	1.000	-2883.128	29	2500	0.988
765034022	-1259.659	68.790	452.352	-1530.977	10	2500	0.996	-1272.523	995	2500	0.602
765195863	-3018.943	154.341	281.737	-3862.284	0	2500	1.000	-3259.119	19	2500	0.992
765256553	-3288.519	161.084	527.652	-4224.186	0	2500	1.000	-3484.173	50	2500	0.980
765317243	-2697.870	129.495	248.665	-3525.338	0	2500	1.000	-2956.778	5	2500	0.998
765337473	-934.443	40.394	120.691	-1353.763	0	2500	1.000	-1030.000	84	2500	0.966
809635352	-3108.150	133.680	211.488	-3750.174	0	2500	1.000	-3247.170	212	2500	0.915
892969023	-3675.875	267.018	1344.946	-3913.415	113	2500	0.955	-3596.724	2110	2500	0.156
953045535	-2148.504	142.710	304.242	-2608.821	1	2500	1.000	-2267.962	157	2500	0.937
970836795	-2040.229	127.583	177.706	-2366.985	10	2496	0.996	-2155.684	151	2496	0.940
Passing Rate							71(98.6%)				71(98.6%)

*, ** See the bottom notes of Table S3 for the interpretations of the table columns here.

Table S6. Test results of fitting MSN (multi-site neutral) model to HMP-whole datasets (*Sites*≥5, Subjects=172)

ID	L_O	θ	*M-value*	*Metacommunity*				*Local community*			
				L_M	$\#N_M$	N	$**P_M$	L_L	$\#N_L$	N	$**P_L$
103092734	-2802.896	1147.772	122.128	-2507.942	2492	2500	0.003	-2649.043	2315	2500	0.074
132902142	-38065.916	3559.293	370.025	-38355.535	600	2500	0.760	-38935.805	25	2500	0.990
147406386	-64235.930	4906.266	292.447	-65074.044	188	2500	0.925	-66558.734	0	2500	1.000
158013734	-56921.895	3961.098	315.871	-57466.786	412	2500	0.835	-58225.131	9	2500	0.996
158114885	-69376.790	3861.186	433.830	-71913.604	0	2500	1.000	-71454.240	0	2500	1.000
158155345	-61013.399	3866.351	339.302	-62350.821	22	2500	0.991	-63220.119	0	2500	1.000
158216035	-69227.089	4256.415	427.091	-72466.739	0	2500	1.000	-72169.707	0	2500	1.000
158236265	-62957.327	4431.729	328.197	-64841.970	4	2500	0.998	-65476.007	0	2500	1.000
158276726	-63975.442	4400.848	235.320	-65622.738	5	2500	0.998	-66699.871	0	2500	1.000
158398106	-111968.178	4588.817	335.041	-119327.494	0	2500	1.000	-117581.943	0	2500	1.000
158418336	-137549.941	5865.038	475.245	-144342.927	0	2500	1.000	-143084.504	0	2500	1.000
158438567	-129074.135	4943.400	390.403	-137760.314	0	2500	1.000	-135798.765	0	2500	1.000
158458797	-133304.453	5548.476	286.065	-138962.394	0	2500	1.000	-139324.180	0	2500	1.000
158479027	-64569.173	5110.481	483.657	-65178.763	322	2500	0.871	-65718.172	18	2500	0.993
158499257	-49143.880	4640.143	354.491	-49670.305	355	2500	0.858	-50314.259	3	2500	0.999
158721788	-60573.677	5151.838	391.469	-61780.709	33	2500	0.987	-62466.191	1	2500	1.000
158742018	-148356.744	5791.396	365.149	-153821.922	0	2500	1.000	-154182.025	0	2500	1.000
158802708	-92934.873	7038.117	454.133	-94650.771	23	2500	0.991	-96133.397	0	2500	1.000
158822939	-133898.400	7288.545	737.928	-138203.162	0	2500	1.000	-140386.496	0	2500	1.000
158883629	-150999.547	4977.649	339.400	-159912.773	0	2500	1.000	-160187.658	0	2500	1.000
158924089	-132288.899	8078.679	655.127	-136208.777	0	2500	1.000	-138482.311	0	2500	1.000
158944319	-79047.792	6220.612	352.028	-81243.747	2	2500	1.000	-82538.374	0	2500	1.000
158964549	-166885.452	5803.914	410.292	-178243.500	0	2500	1.000	-175949.183	0	2500	1.000
158984779	-51908.200	5571.336	234.776	-51493.223	1925	2500	0.230	-53031.061	49	2500	0.980
159005010	-104996.955	6135.541	468.697	-109520.515	0	2500	1.000	-109922.267	0	2500	1.000
159025240	-63806.525	3829.799	346.890	-68285.291	0	2500	1.000	-66845.899	0	2500	1.000
159085930	-110693.361	7641.466	404.650	-112989.188	8	2500	0.997	-115193.247	0	2500	1.000
159146620	-181361.662	5867.754	519.312	-198873.162	0	2500	1.000	-192821.268	0	2500	1.000
159166850	-246683.950	6791.554	583.939	-260383.071	0	2500	1.000	-263137.709	0	2500	1.000
159227541	-150966.757	5099.439	436.421	-160946.613	0	2500	1.000	-158434.007	0	2500	1.000
159247771	-77638.165	4924.496	374.144	-80758.990	0	2500	1.000	-80777.636	0	2500	1.000
159389382	-128859.539	5258.571	411.029	-135380.878	0	2500	1.000	-134643.025	0	2500	1.000
159470302	-140635.251	7108.914	548.218	-152318.311	0	2500	1.000	-150717.397	0	2500	1.000
159713063	-135683.219	7227.879	476.745	-141125.445	0	2500	1.000	-143691.688	0	2500	1.000
159733294	-127916.425	6918.788	498.363	-135237.482	0	2500	1.000	-135533.777	0	2500	1.000
160744799	-30475.562	2502.538	230.362	-31270.134	53	2499	0.979	-31280.402	21	2499	0.992
160825720	-85634.992	5260.301	401.332	-84518.152	2427	2496	0.028	-85990.193	602	2496	0.759
160866180	-78466.641	5015.270	257.286	-77975.399	2028	2500	0.189	-79423.282	112	2500	0.955
160886410	-40206.154	3974.115	345.299	-39276.226	2479	2500	0.008	-40272.892	1077	2500	0.569
160906640	-46135.594	4557.317	419.690	-45839.934	1881	2500	0.248	-46448.847	494	2500	0.802
160947100	-90727.056	5196.394	488.723	-91321.874	413	2498	0.835	-91452.365	173	2498	0.931
160967330	-45658.062	4349.304	412.424	-43845.880	2500	2500	0.000	-45165.488	2291	2500	0.084
160987560	-44353.674	4704.254	507.614	-42275.115	2500	2500	0.000	-43653.542	2462	2500	0.015
161007791	-42908.976	3769.858	333.667	-41821.949	2486	2500	0.006	-42887.227	1307	2500	0.477
161270782	-44965.779	5788.948	370.538	-42302.170	2496	2497	0.000	-43884.916	2492	2497	0.002
161311242	-37053.833	4287.248	409.961	-35060.833	2500	2500	0.000	-36366.133	2469	2500	0.012
161331472	-11287.555	4571.672	328.843	-9588.195	2500	2500	0.000	-10135.824	2500	2500	0.000

161351702	-8731.369	3850.748	443.719	-7173.746	2500	2500	0.000	-7672.465	2500	2500	0.000
161412393	-29465.842	3118.651	478.994	-29842.246	362	2500	0.855	-29997.803	94	2500	0.962
161473083	-48853.132	5561.309	217.569	-47517.933	2492	2500	0.003	-48959.607	1024	2500	0.590
161554003	-40723.769	3469.835	273.241	-40916.725	801	2500	0.680	-41694.892	10	2500	0.996
178713055	-43727.432	4204.176	249.628	-44110.360	548	2499	0.781	-44925.711	4	2499	0.998
184349034	-36519.128	7099.860	319.325	-34765.011	2500	2500	0.000	-36136.941	2090	2500	0.164
208027353	-67635.551	4650.735	351.503	-68798.123	60	2500	0.976	-69574.574	0	2500	1.000
246515023	-43927.337	4084.862	393.402	-42592.755	2499	2500	0.000	-43755.629	1679	2500	0.328
256789458	-32556.642	5685.051	232.413	-31373.192	2494	2498	0.002	-32577.512	1193	2498	0.522
275382046	-22630.954	4934.000	211.065	-21470.087	2497	2500	0.001	-22382.471	1924	2500	0.230
289996019	-101213.721	4043.641	306.756	-107313.527	0	2500	1.000	-107232.553	0	2500	1.000
295137534	-32094.664	4327.608	245.921	-30529.873	2500	2500	0.000	-31625.609	2279	2500	0.088
336497421	-49526.861	4803.265	380.720	-48600.642	2456	2500	0.018	-49838.522	537	2500	0.785
338793263	-10942.637	1824.286	260.056	-11059.199	772	2500	0.691	-11230.772	273	2500	0.891
355657046	-16230.004	2605.727	391.248	-15764.754	2408	2500	0.037	-16291.568	967	2500	0.613
368533040	-46785.829	3857.425	378.521	-46135.226	2323	2500	0.071	-47104.479	515	2500	0.794
370027359	-62466.274	4952.765	303.374	-62615.940	1000	2500	0.600	-64270.076	1	2500	1.000
370425937	-65649.558	3981.281	258.597	-67296.029	2	2500	1.000	-67931.269	0	2500	1.000
375450439	-118249.041	4911.966	359.786	-124432.032	0	2500	1.000	-123236.357	0	2500	1.000
404239096	-56209.528	5219.827	370.455	-55598.779	2233	2500	0.107	-56903.128	161	2500	0.936
414519462	-66769.089	5066.222	295.080	-67856.164	85	2500	0.966	-68999.387	0	2500	1.000
451588811	-53061.005	5768.368	314.410	-52236.677	2377	2500	0.049	-53955.294	58	2500	0.977
465578759	-63774.256	5323.203	222.482	-65610.899	3	2500	0.999	-66860.787	0	2500	1.000
492786515	-46918.000	3973.512	246.555	-47535.462	248	2500	0.901	-48389.616	1	2500	1.000
508703490	-40148.466	3592.284	195.346	-40218.394	1081	2500	0.568	-41221.437	13	2500	0.995
514014184	-5676.375	4655.542	214.537	-4734.582	2500	2500	0.000	-4942.818	2500	2500	0.000
516889361	-31593.672	9500.009	311.070	-29180.016	2500	2500	0.000	-30379.113	2499	2500	0.000
517810313	-64156.773	4080.032	326.507	-66196.860	0	2500	1.000	-66875.023	0	2500	1.000
550534656	-14418.532	1900.497	338.457	-15054.421	19	2500	0.992	-14967.010	16	2500	0.994
553359145	-63025.846	4841.546	252.634	-63652.900	347	2500	0.861	-65205.799	0	2500	1.000
561079058	-7484.100	2952.811	123.537	-7105.924	2389	2500	0.044	-7398.863	1623	2500	0.351
604812005	-33950.019	4435.734	411.985	-31799.551	2500	2500	0.000	-32918.625	2499	2500	0.000
612472597	-86269.739	6613.476	362.351	-87190.839	264	2500	0.894	-88352.322	3	2500	0.999
638754422	-54888.972	5913.333	292.298	-53385.460	2496	2500	0.002	-55179.071	675	2500	0.730
643185023	-34626.267	4963.173	188.741	-34008.228	2307	2496	0.076	-35073.982	345	2496	0.862
650853796	-48233.805	5689.093	322.374	-47296.678	2438	2500	0.025	-48550.530	572	2500	0.771
658594300	-18762.503	3269.391	191.659	-18144.054	2452	2500	0.019	-18795.546	1124	2500	0.550
663835652	-55789.060	4708.026	329.731	-56935.634	32	2500	0.987	-57568.156	0	2500	1.000
668248235	-73125.878	5332.918	344.053	-74205.504	99	2500	0.960	-75695.150	0	2500	1.000
675950834	-16654.664	4942.781	331.591	-15018.435	2500	2500	0.000	-15791.131	2499	2500	0.000
682102541	-19329.785	4253.551	207.005	-19010.878	2074	2500	0.170	-19721.189	259	2500	0.896
682449369	-64313.159	3774.025	354.786	-65104.396	203	2500	0.919	-66001.731	0	2500	1.000
686765762	-98543.786	5223.220	343.220	-99686.453	120	2500	0.952	-100891.723	0	2500	1.000
706846339	-25284.463	7913.746	309.791	-23263.015	2500	2500	0.000	-24277.876	2495	2500	0.002
737052003	-74465.495	5238.773	285.792	-76876.727	0	2500	1.000	-77921.892	0	2500	1.000
739574095	-15954.499	2827.819	127.295	-16173.207	631	2500	0.748	-16759.271	8	2500	0.997
763395383	-69124.253	4835.659	424.348	-70720.956	9	2500	0.996	-71346.094	0	2500	1.000
763435843	-73166.731	5276.904	376.370	-75282.583	0	2498	1.000	-76257.706	0	2498	1.000
763456073	-59812.211	3548.038	318.717	-62420.161	0	2500	1.000	-62170.529	0	2500	1.000
763476303	-49008.171	4614.024	224.827	-49629.428	298	2500	0.881	-50868.207	0	2500	1.000
763516763	-96859.532	5143.585	257.024	-100635.541	0	2500	1.000	-101327.566	0	2500	1.000

763536994	-195412.579	5964.464	362.468	-208852.061	0	2500	1.000	-207068.489	0	2500	1.000
763557224	-36154.040	3248.713	176.365	-36813.719	198	2500	0.921	-37587.942	0	2500	1.000
763597684	-201279.291	6863.464	535.301	-215779.390	0	2500	1.000	-214280.842	0	2500	1.000
763638144	-92656.142	4522.619	306.620	-95845.469	0	2500	1.000	-95934.316	0	2500	1.000
763678604	-23417.894	1219.138	449.452	-29274.734	0	2500	1.000	-25493.672	0	2500	1.000
763698834	-198271.787	6547.461	341.459	-209683.826	0	2500	1.000	-213045.539	0	2500	1.000
763719065	-86098.522	3644.971	231.988	-88953.756	0	2500	1.000	-89275.721	0	2500	1.000
763759525	-129191.795	6150.275	267.835	-133891.259	0	2500	1.000	-135484.524	0	2500	1.000
763820215	-81574.881	3704.069	200.298	-87229.701	0	2500	1.000	-86715.139	0	2500	1.000
763840445	-142906.564	5403.531	323.128	-149749.361	0	2500	1.000	-149425.566	0	2500	1.000
763860675	-121373.894	5243.776	349.145	-128409.731	0	2500	1.000	-126976.155	0	2500	1.000
763880905	-75134.674	4367.088	283.445	-78571.615	0	2500	1.000	-79817.887	0	2500	1.000
763901136	-110998.993	5850.153	297.546	-112856.259	19	2500	0.992	-114540.189	0	2500	1.000
763921366	-73581.503	5005.174	314.424	-75259.977	14	2500	0.994	-76530.583	0	2500	1.000
763982056	-131017.817	5302.332	330.953	-139584.668	0	2500	1.000	-138582.600	0	2500	1.000
764002286	-138649.116	5421.903	328.263	-146495.002	0	2500	1.000	-147744.711	0	2500	1.000
764042746	-86625.726	4649.861	259.511	-87747.859	141	2500	0.944	-89185.640	1	2500	1.000
764062976	-108244.747	4781.596	258.174	-112264.475	0	2500	1.000	-112874.012	0	2500	1.000
764083206	-162921.341	5176.445	422.615	-175710.872	0	2500	1.000	-173020.272	0	2500	1.000
764143897	-145980.002	4718.025	327.599	-155523.831	0	2500	1.000	-153877.898	0	2500	1.000
764184357	-123118.900	4867.863	308.907	-127828.636	0	2500	1.000	-128141.545	0	2500	1.000
764224817	-127111.172	5575.574	351.718	-133167.877	0	2500	1.000	-132796.098	0	2500	1.000
764245047	-44499.839	3459.971	181.600	-45961.504	11	2500	0.996	-46639.969	0	2500	1.000
764285508	-42328.881	2431.382	436.309	-44368.260	0	2500	1.000	-43917.520	0	2500	1.000
764305738	-188881.032	6802.044	454.587	-201542.409	0	2500	1.000	-201022.558	0	2500	1.000
764325968	-133161.919	6573.226	341.988	-136292.821	0	2500	1.000	-138074.817	0	2500	1.000
764346198	-99372.608	5493.579	255.072	-100402.913	215	2500	0.914	-102378.479	0	2500	1.000
764366428	-40219.653	3188.345	181.735	-40997.768	141	2500	0.944	-41895.434	1	2500	1.000
764447348	-88520.364	5049.612	238.362	-92094.458	0	2500	1.000	-92361.623	0	2500	1.000
764467579	-87400.557	4536.065	334.681	-89703.086	0	2500	1.000	-90427.220	0	2500	1.000
764487809	-116279.529	5652.887	295.371	-122449.014	0	2500	1.000	-122157.448	0	2500	1.000
764508039	-57353.763	5302.357	284.110	-58009.374	300	2500	0.880	-59100.013	0	2500	1.000
764588959	-46949.519	3393.813	229.023	-47804.697	125	2500	0.950	-48454.772	0	2500	1.000
764649650	-66985.592	5507.387	265.167	-67555.812	425	2500	0.830	-68946.035	1	2500	1.000
764669880	-117257.588	4707.120	417.667	-123558.474	0	2500	1.000	-121982.216	0	2500	1.000
764710340	-11016.876	2167.290	71.451	-10591.345	2362	2500	0.055	-10967.846	1444	2500	0.422
764750800	-70837.426	5117.257	455.311	-72902.661	0	2500	1.000	-73260.962	0	2500	1.000
764811490	-78875.772	6017.034	309.347	-80005.251	125	2500	0.950	-81743.024	0	2500	1.000
764831721	-60449.510	5113.365	417.770	-61677.788	27	2500	0.989	-62121.341	0	2500	1.000
764872181	-55622.311	3664.676	341.609	-57720.075	0	2500	1.000	-57914.282	0	2500	1.000
764892411	-59415.354	4361.149	367.091	-60279.184	143	2500	0.943	-60785.985	1	2500	1.000
764953101	-56181.522	4989.529	413.988	-57194.504	59	2500	0.976	-57729.393	0	2500	1.000
765013792	-55636.487	3849.749	317.947	-57254.325	3	2500	0.999	-57528.785	0	2500	1.000
765034022	-54871.813	3794.996	297.712	-56151.373	16	2500	0.994	-56545.665	0	2500	1.000
765074482	-89036.892	5650.346	333.606	-89894.988	250	2500	0.900	-91193.818	0	2500	1.000
765094712	-103141.480	4725.479	371.284	-107684.581	0	2500	1.000	-107783.179	0	2500	1.000
765135172	-4729.347	462.125	1240.223	-5079.471	20	2500	0.992	-4463.160	2482	2500	0.007
765155402	-63387.349	5002.686	294.641	-63426.140	1188	2500	0.525	-64739.860	11	2500	0.996
765195863	-66737.889	5431.455	278.063	-66771.605	1194	2500	0.522	-68202.435	11	2500	0.996
765216093	-64468.688	4904.974	324.773	-64956.513	494	2500	0.802	-66274.322	0	2500	1.000
765256553	-55396.922	4108.259	359.594	-56775.312	13	2500	0.995	-57260.235	0	2500	1.000

765276783	-60086.505	4650.883	285.444	-60884.732	171	2500	0.932	-62255.246	0	2500	1.000
765317243	-42952.911	3462.816	242.556	-44533.631	2	2500	1.000	-44825.660	0	2500	1.000
765337473	-66258.526	5014.517	293.241	-66538.026	757	2500	0.697	-68035.844	0	2500	1.000
765377934	-45350.915	3784.215	394.378	-45713.364	570	2500	0.772	-46744.238	0	2500	1.000
765398164	-4687.293	728.674	234.497	-4682.253	1287	2500	0.485	-4769.107	639	2500	0.744
765519544	-12036.450	667.678	2499.293	-14380.031	0	2500	1.000	-11996.478	1513	2500	0.395
765539774	-7273.177	1218.710	237.910	-7154.431	1904	2500	0.238	-7385.910	588	2500	0.765
765560005	-79462.204	4794.541	432.980	-81594.764	1	2500	1.000	-82473.509	0	2500	1.000
765620695	-3935.003	1736.961	97.399	-3731.699	2257	2500	0.097	-3893.153	1528	2500	0.389
765640925	-6139.936	564.094	3294.446	-6908.787	0	2500	1.000	-6040.786	2041	2500	0.184
765661155	-4943.845	598.035	553.157	-5650.425	0	2500	1.000	-5128.814	159	2500	0.936
765701615	-14337.057	831.027	1850.574	-17306.163	0	2500	1.000	-14375.918	1047	2500	0.581
809635352	-71314.294	5439.099	344.016	-71206.699	1432	2500	0.427	-72839.505	6	2500	0.998
823052294	-7274.195	827.923	1416.260	-7472.839	258	2500	0.897	-6941.690	2489	2500	0.004
857980665	-14682.835	3181.971	282.503	-14031.830	2481	2500	0.008	-14646.178	1408	2500	0.437
861967750	-11682.510	1487.437	375.029	-12029.517	132	2500	0.947	-11929.118	255	2500	0.898
863126187	-65277.004	5056.248	441.164	-66358.091	82	2500	0.967	-67402.072	0	2500	1.000
875002022	-7625.551	1791.123	132.851	-7354.292	2271	2500	0.092	-7648.549	1110	2500	0.556
892969023	-45311.523	4231.301	333.840	-44001.021	2499	2500	0.000	-45310.503	1252	2500	0.499
901775393	-7203.285	5404.161	176.168	-6267.593	2500	2500	0.000	-6483.295	2499	2500	0.000
937495960	-40082.257	5397.751	295.511	-38954.943	2489	2500	0.004	-40001.830	1430	2500	0.428
953045535	-57380.172	5630.590	248.794	-56817.572	2119	2500	0.152	-58368.016	68	2500	0.973
970836795	-56476.715	4756.746	312.412	-56196.471	1759	2500	0.296	-57472.673	38	2500	0.985
Passing Rate						140 (81.4%)				159 (92.4%)	

*, ** See the bottom notes of Table S3 for the interpretations of the table columns here.

Table S7. Test results of fitting MSN (multi-site neutral) model to Gut datasets (*Sites*=7, *Subjects*=11)

ID	L_O	θ	*M-value*	*Metacommunity*				*Local community*			
				L_M	N_M	#N	$**P_M$	L_L	$\#N_L$	N	$**P_L$
S400	-16579.634	793.343	471.258	-17899.558	0	2500	1.000	-16962.178	100	2500	0.960
S401	-19651.717	1043.292	655.925	-21249.517	0	2500	1.000	-19792.435	669	2500	0.732
S402	-18346.953	721.944	1283.276	-21074.910	0	2500	1.000	-17978.520	2385	2500	0.046
S403	-22074.336	1046.688	881.515	-24182.092	0	2500	1.000	-22327.449	309	2500	0.876
S404	-18179.815	726.583	970.568	-20893.803	0	2500	1.000	-17866.340	2254	2500	0.098
S405	-19460.183	733.333	1287.729	-22214.823	0	2500	1.000	-19480.938	1157	2500	0.537
S406	-17741.984	679.434	731.527	-21924.779	0	2500	1.000	-18145.858	80	2500	0.968
S407	-6527.371	225.626	1610.896	-6902.900	140	2500	0.944	-6500.625	1511	2500	0.396
S408	-16091.542	592.197	3864.940	-18472.707	0	2500	1.000	-15528.575	2498	2500	0.001
S409	-11784.623	583.757	1965.148	-12257.688	86	2500	0.966	-11490.875	2439	2500	0.024
S410	-12919.475	590.553	2455.416	-13190.723	422	2500	0.831	-12815.262	1926	2500	0.230
Passing rate							11 (100%)				8 (72.7%)

*, ** See the bottom notes of Table S3 for the interpretations of the table columns here.

Table S8. Test results of fitting MSN (multi-site neutral) model to Lung datasets (*Sites*=4, *Subjects*=139)

ID	L_O	θ	*M-value*	Metacommunity				Local community			
				L_M	$\#N_M$	N	$**P_M$	L_L	$\#N_L$	N	$**P_L$
A1	-481.585	13.308	32.427	-633.426	139	2500	0.944	-500.514	791	2500	0.684
A2	-635.672	39.788	10.439	-739.906	223	2500	0.911	-697.429	371	2500	0.852
A3	-831.167	32.854	59.980	-985.861	139	2500	0.944	-857.579	795	2500	0.682
A4	-633.801	38.131	16.412	-782.176	68	2500	0.973	-703.590	279	2500	0.888
A5	-775.792	26.854	61.989	-1076.234	14	2500	0.994	-815.169	567	2500	0.773
A6	-632.081	44.385	23.340	-721.606	252	2500	0.899	-677.711	490	2500	0.804
A7	-981.717	54.383	26.073	-1124.542	134	2500	0.946	-1070.326	195	2500	0.922
A8	-1202.864	37.713	116.219	-1398.509	129	2500	0.948	-1228.659	797	2500	0.681
A9	-806.786	24.873	140.023	-921.278	309	2500	0.876	-817.724	1021	2500	0.592
A10	-1152.946	53.405	58.510	-1416.979	21	2500	0.992	-1217.641	404	2500	0.838
A11	-480.336	17.786	22.133	-666.582	33	2500	0.987	-527.427	421	2500	0.832
A12	-881.569	31.078	38.842	-1008.703	280	2500	0.888	-923.646	536	2500	0.786
A13	-782.355	37.205	44.645	-1092.645	1	2500	1.000	-847.280	347	2500	0.861
A14	-944.301	26.664	149.449	-1113.594	171	2500	0.932	-973.140	672	2500	0.731
A15	-717.709	29.519	80.491	-919.796	53	2500	0.979	-742.275	767	2500	0.693
A16	-762.857	33.461	27.957	-967.129	41	2500	0.984	-818.959	392	2500	0.843
A17	-1005.353	44.832	69.270	-1348.469	3	2500	0.999	-1041.834	708	2500	0.717
A18	-918.551	26.494	196.899	-1165.704	52	2500	0.979	-917.082	1277	2500	0.489
A19	-1003.853	39.133	72.820	-1171.175	138	2500	0.945	-1028.952	827	2500	0.669
A20	-1301.418	41.270	133.094	-1521.702	120	2500	0.952	-1296.284	1346	2500	0.462
A21	-500.021	22.420	14.574	-631.438	122	2500	0.951	-564.609	232	2500	0.907
A22	-747.543	26.786	49.008	-956.691	43	2500	0.983	-790.220	487	2500	0.805
A23	-1112.297	65.279	25.877	-1328.097	36	2500	0.986	-1203.742	269	2500	0.892
A24	-646.261	28.879	26.323	-861.529	23	2500	0.991	-703.559	413	2500	0.835
A25	-433.653	54.235	4.458	-472.734	623	2500	0.751	-477.749	547	2500	0.781
A26	-881.594	26.775	113.427	-1144.461	39	2500	0.984	-904.920	751	2500	0.700
A27	-879.377	33.017	81.602	-1096.477	44	2500	0.982	-864.733	1519	2500	0.392
A28	-1392.224	49.617	184.111	-1560.640	176	2500	0.930	-1404.236	1043	2500	0.583
A29	-601.319	22.443	130.658	-773.921	72	2500	0.971	-609.563	1028	2500	0.589
A30	-1102.499	45.232	86.805	-1335.388	36	2500	0.986	-1130.304	798	2500	0.681
A31	-466.648	12.703	31.878	-604.759	144	2500	0.942	-491.886	650	2500	0.740
A32	-1493.402	136.344	28.161	-1563.690	608	2500	0.757	-1548.852	610	2500	0.756
A33	-850.605	30.923	36.715	-1014.384	138	2500	0.945	-893.000	539	2500	0.784
A34	-340.220	9.239	85.438	-462.065	120	2500	0.952	-347.918	993	2500	0.603
A35	-933.312	45.328	26.791	-1113.902	71	2500	0.972	-1008.715	274	2500	0.890
A36	-365.316	8.821	13.723	-399.360	831	2500	0.668	-374.947	941	2500	0.624
A37	-327.078	11.743	8.040	-397.446	388	2500	0.845	-357.555	542	2500	0.783
A38	-696.465	38.329	15.625	-860.874	60	2500	0.976	-785.245	144	2500	0.942
A39	-377.544	12.715	52.068	-490.203	164	2500	0.934	-393.927	762	2500	0.695
A40	-593.877	24.858	35.520	-679.450	313	2500	0.875	-626.410	555	2500	0.778
A41	-700.266	85.026	10.705	-693.222	1347	2500	0.461	-716.221	987	2500	0.605
A42	-523.382	27.063	18.291	-602.651	329	2500	0.868	-565.827	434	2500	0.826
A43	-422.401	34.950	10.098	-460.018	652	2500	0.739	-460.077	471	2500	0.812
A44	-1164.002	78.734	48.656	-1248.952	442	2500	0.823	-1210.345	544	2500	0.782
A45	-801.696	40.567	71.067	-910.122	248	2500	0.901	-807.856	1130	2500	0.548
A46	-402.368	19.322	31.726	-572.693	26	2500	0.990	-432.726	621	2500	0.752
A47	-239.720	39.831	2.319	-257.986	851	2500	0.660	-265.789	668	2500	0.733
A48	-375.561	49.332	5.090	-432.249	422	2500	0.831	-439.235	251	2500	0.900

A49	-769.923	59.168	28.064	-875.205	199	2500	0.920	-828.269	416	2500	0.834
A50	-637.291	37.821	174.090	-716.987	332	2500	0.867	-633.384	1351	2500	0.460
A51	-592.193	39.520	15.818	-683.722	229	2500	0.908	-651.593	270	2500	0.892
A52	-692.850	30.303	58.519	-816.472	179	2500	0.928	-722.260	644	2500	0.742
A53	-524.058	46.398	10.832	-596.332	345	2500	0.862	-591.201	246	2500	0.902
A54	-718.359	30.362	56.328	-843.279	186	2500	0.926	-741.836	784	2500	0.686
A55	-556.148	42.928	12.851	-626.349	359	2500	0.856	-619.660	257	2500	0.897
A56	-1168.686	52.412	117.425	-1278.120	362	2500	0.855	-1177.683	1096	2500	0.562
A57	-708.815	85.690	14.672	-761.435	553	2500	0.779	-766.497	452	2500	0.819
A58	-475.024	18.111	79.112	-638.999	59	2500	0.976	-489.821	882	2500	0.647
A59	-581.983	143.288	17.633	-567.668	1460	2500	0.416	-591.224	1068	2500	0.573
A60	-684.417	37.724	41.429	-863.959	37	2500	0.985	-723.475	557	2500	0.777
A61	-356.233	49.132	7.863	-399.628	483	2500	0.807	-389.665	657	2500	0.737
A62	-279.578	10.139	53.048	-403.566	86	2500	0.966	-292.145	889	2500	0.644
A63	-564.309	34.975	24.473	-694.842	78	2500	0.969	-611.486	434	2500	0.826
A64	-631.630	24.088	110.597	-799.006	66	2500	0.974	-640.413	1041	2500	0.584
A65	-832.641	28.564	445.516	-953.871	236	2500	0.906	-850.814	787	2500	0.685
A66	-745.308	31.213	68.785	-852.050	287	2500	0.885	-771.489	721	2500	0.712
A67	-1117.974	49.408	263.954	-1292.837	92	2500	0.963	-1127.834	1080	2500	0.568
A68	-414.720	16.437	22.675	-486.876	419	2500	0.832	-441.165	597	2500	0.761
A69	-236.445	8.034	31.924	-336.590	152	2500	0.939	-248.950	838	2500	0.665
A70	-779.776	44.096	39.536	-928.667	83	2500	0.967	-840.604	318	2500	0.873
A71	-781.595	36.752	135.561	-957.130	56	2500	0.978	-796.926	955	2500	0.618
A72	-190.788	14.231	3.277	-236.116	398	2500	0.841	-228.131	350	2500	0.860
A73	-359.425	19.878	81.326	-396.710	635	2500	0.746	-352.362	1441	2500	0.424
A74	-134.719	6.161	27.229	-190.665	222	2500	0.911	-138.465	1092	2500	0.563
A75	-488.689	34.104	12.370	-566.448	295	2500	0.882	-535.961	416	2500	0.834
A76	-513.202	23.556	96.329	-736.375	3	2500	0.999	-537.589	698	2500	0.721
A77	-658.369	28.604	100.971	-856.826	35	2500	0.986	-682.462	723	2500	0.711
A78	-491.325	22.261	26.939	-578.895	274	2500	0.890	-512.091	841	2500	0.664
A79	-683.622	59.319	18.110	-772.618	239	2500	0.904	-741.843	364	2500	0.854
A80	-487.943	17.901	84.320	-566.840	355	2500	0.858	-472.119	1625	2500	0.350
A81	-575.185	35.644	14.130	-631.418	546	2500	0.782	-633.004	303	2500	0.879
A82	-501.780	27.089	91.015	-648.984	36	2500	0.986	-535.411	495	2500	0.802
A83	-676.014	27.378	119.435	-872.139	36	2500	0.986	-683.056	1096	2500	0.562
A84	-845.225	37.586	91.317	-1024.070	75	2500	0.970	-869.465	807	2500	0.677
A85	-546.472	40.104	14.002	-608.324	442	2500	0.823	-591.110	509	2500	0.796
A86	-257.900	12.178	36.144	-370.616	67	2500	0.973	-271.466	858	2500	0.657
A87	-646.742	21.854	120.606	-736.411	380	2500	0.848	-650.484	1143	2500	0.543
A88	-632.478	65.742	11.103	-683.905	526	2500	0.790	-699.938	315	2500	0.874
A89	-582.524	35.163	19.891	-677.434	217	2500	0.913	-625.529	526	2500	0.790
A90	-828.200	32.852	164.205	-940.965	265	2500	0.894	-805.981	1712	2500	0.315
A91	-588.770	79.764	13.318	-613.797	844	2500	0.662	-618.909	771	2500	0.692
A92	-668.989	23.558	113.201	-785.512	226	2500	0.910	-678.852	1007	2500	0.597
A93	-561.680	20.748	62.370	-679.304	179	2500	0.928	-580.378	793	2500	0.683
A94	-570.575	34.380	15.664	-641.455	397	2500	0.841	-614.161	482	2500	0.807
A95	-300.648	28.962	5.288	-364.052	288	2500	0.885	-356.426	270	2500	0.892
A96	-669.229	36.580	50.111	-760.998	284	2500	0.886	-681.169	1024	2500	0.590
A97	-535.374	32.079	74.734	-588.065	519	2500	0.792	-539.700	1138	2500	0.545
A98	-235.909	7.642	64.477	-329.876	170	2500	0.932	-244.575	877	2500	0.649
A99	-341.040	15.080	22.197	-459.011	116	2500	0.954	-372.943	549	2500	0.780

A100	-530.120	58.145	8.798	-591.725	394	2500	0.842	-586.470	353	2500	0.859
A101	-328.432	20.812	31.086	-395.005	287	2500	0.885	-352.157	638	2500	0.745
A102	-544.809	32.459	106.771	-639.924	202	2500	0.919	-524.912	1666	2500	0.334
A103	-711.908	60.612	13.583	-755.577	664	2500	0.734	-786.569	213	2500	0.915
A104	-372.289	49.530	5.210	-380.714	1102	2500	0.559	-388.904	926	2500	0.630
A105	-979.389	54.562	58.532	-1116.589	147	2500	0.941	-1019.704	564	2500	0.774
A106	-245.519	14.699	19.486	-281.792	550	2500	0.780	-257.963	844	2500	0.662
A107	-976.774	47.044	80.998	-1021.825	740	2500	0.704	-985.838	1080	2500	0.568
A108	-462.724	16.520	50.376	-545.481	331	2500	0.868	-472.348	988	2500	0.605
A109	-446.153	16.614	70.941	-578.916	100	2500	0.960	-452.445	1069	2500	0.572
A110	-68.691	2.107	23.383	-118.290	333	2500	0.867	-70.553	1148	2500	0.541
A111	-377.544	17.657	21.558	-506.777	81	2500	0.968	-414.802	492	2500	0.803
R01	-665.016	30.522	15.905	-797.250	170	2500	0.932	-726.584	314	2500	0.874
R02	-926.502	48.011	34.118	-1097.671	69	2500	0.972	-978.269	527	2500	0.789
R03	-630.347	37.146	13.062	-794.112	67	2500	0.973	-721.818	145	2500	0.942
R04	-824.575	23.776	103.929	-1059.826	45	2500	0.982	-837.462	977	2500	0.609
R05	-320.844	7.614	23.280	-408.870	316	2500	0.874	-331.238	946	2500	0.622
R06	-264.228	11.428	24.178	-498.473	1	2500	1.000	-298.934	512	2500	0.795
R07	-521.993	18.887	52.933	-803.576	3	2500	0.999	-541.411	840	2500	0.664
R08	-595.801	34.836	10.318	-681.581	309	2500	0.876	-679.910	125	2500	0.950
R09	-1009.911	36.269	47.874	-1182.466	130	2500	0.948	-1050.154	595	2500	0.762
R10	-838.034	36.327	28.819	-1070.206	23	2500	0.991	-930.290	129	2500	0.948
R11	-1461.111	53.252	171.399	-1765.924	26	2500	0.990	-1485.674	857	2500	0.657
R12	-968.690	59.388	20.164	-1074.740	295	2500	0.882	-1040.145	353	2500	0.859
R13	-347.702	27.445	4.441	-376.901	743	2500	0.703	-388.750	465	2500	0.814
R14	-792.902	50.885	37.197	-929.453	105	2500	0.958	-816.782	916	2500	0.634
R15	-1068.437	33.697	109.968	-1323.935	36	2500	0.986	-1093.837	805	2500	0.678
R16	-632.163	22.894	48.285	-884.079	26	2500	0.990	-656.418	748	2500	0.701
R17	-872.927	28.039	64.287	-1087.557	62	2500	0.975	-914.444	521	2500	0.792
R18	-998.375	31.082	139.459	-1219.017	68	2500	0.973	-1035.500	590	2500	0.764
R19	-1026.748	40.335	79.857	-1325.459	11	2500	0.996	-1076.786	518	2500	0.793
R20	-489.987	34.580	9.058	-593.647	147	2500	0.941	-559.801	202	2500	0.919
R22	-871.992	33.939	32.549	-1032.431	142	2500	0.943	-935.371	330	2500	0.868
R24	-909.782	32.311	62.581	-1186.178	17	2500	0.993	-953.761	526	2500	0.790
R25	-1497.358	55.279	198.425	-1863.323	9	2500	0.996	-1536.909	655	2500	0.738
R26	-643.863	29.236	57.518	-1032.539	0	2500	1.000	-704.807	327	2500	0.869
R27	-981.255	49.745	80.729	-1230.919	17	2500	0.993	-1016.385	721	2500	0.712
R28	-723.283	33.166	19.654	-895.698	81	2500	0.968	-784.316	311	2500	0.876
R29	-1036.742	35.963	94.123	-1320.895	22	2500	0.991	-1076.479	629	2500	0.748
R30	-944.673	63.054	21.946	-1113.432	72	2500	0.971	-1028.655	249	2500	0.900
Passing rate							139 (100%)				139 (100%)

*, ** See the bottom notes of Table S3 for the interpretations of the table columns here.

Table S9. Test results of fitting NNH (multi-site neutral) model to HMP-oral datasets (*Sites*≥2, *Subjects*=146)

Group ID	J	S	θ	m	x	γ	R^2	χ^2	p-value	N_{pass}	$\%_{(pass)}$
132902142	6648.500	1061.167	1132.737	0.000	0.472	1.010	0.999	28.682	0.001	4	66.7
147406386	5582.889	906.889	971.944	0.000	0.481	1.009	0.999	39.912	0.000	3	33.3
158013734	2945.889	783.444	1080.420	0.000	0.419	1.013	0.999	37.848	0.000	4	44.4
158114885	3804.778	988.444	1201.062	0.000	0.450	1.005	0.999	35.491	0.000	2	22.2
158155345	3687.778	785.556	894.895	0.000	0.466	1.009	0.999	49.619	0.000	2	22.2
158216035	5623.556	1013.889	1032.861	0.000	0.494	1.005	0.999	29.593	0.001	1	11.1
158236265	4897.500	979.000	1222.320	0.000	0.434	1.020	0.999	116.264	0.000	4	50.0
158276726	6286.444	819.444	819.624	0.000	0.490	1.012	0.999	36.296	0.000	4	44.4
158398106	5072.167	780.944	786.056	0.000	0.495	1.006	0.999	63.180	0.000	7	38.9
158418336	5351.588	1009.000	1180.896	0.000	0.440	1.015	0.998	127.468	0.000	12	70.6
158438567	8502.056	1108.722	1055.604	0.000	0.501	1.007	0.999	88.397	0.000	11	61.1
158458797	7066.944	987.167	994.280	0.000	0.484	1.006	0.999	72.837	0.000	4	22.2
158479027	5238.556	1026.778	1217.791	0.000	0.448	1.015	0.998	67.947	0.000	4	44.4
158499257	4589.556	879.333	1138.474	0.000	0.425	1.022	0.999	95.084	0.000	5	55.6
158721788	5271.889	918.111	1182.532	0.000	0.420	1.030	0.998	174.147	0.000	6	66.7
158742018	5597.278	1009.611	1094.233	0.000	0.466	1.009	0.999	90.358	0.000	13	72.2
158802708	7309.667	1109.556	1277.741	0.000	0.468	1.005	1.000	29.009	0.001	3	33.3
158822939	13154.222	1760.889	1709.656	0.000	0.507	1.000	1.000	40.559	0.000	4	44.4
158883629	7604.722	1097.000	1197.944	0.000	0.476	1.009	0.999	407.160	0.000	8	44.4
158924089	16954.444	1923.889	1834.747	0.000	0.500	1.003	1.000	16.368	0.090	4	44.4
158944319	7920.000	1052.556	1118.341	0.000	0.475	1.017	0.999	105.979	0.000	6	66.7
158964549	8978.778	1207.778	1188.980	0.000	0.485	1.010	0.999	74.751	0.000	8	44.4
158984779	5502.000	700.571	703.005	0.000	0.484	1.028	0.997	93.340	0.000	4	57.1
159005010	10032.667	1615.778	1662.267	0.000	0.488	1.007	1.000	29.654	0.001	4	44.4
159025240	5305.222	862.333	1018.033	0.000	0.451	1.013	0.999	70.482	0.000	5	55.6
159085930	9390.889	1416.111	1401.282	0.000	0.491	1.009	1.000	46.413	0.000	4	44.4
159146620	11161.556	1404.333	1406.052	0.000	0.490	1.013	0.999	113.157	0.000	9	50.0
159166850	12632.667	1486.111	1430.451	0.000	0.494	1.013	0.999	146.382	0.000	12	66.7
159227541	6197.278	1163.833	1319.220	0.000	0.466	1.010	0.999	74.897	0.000	6	33.3
159247771	6674.889	1208.556	1420.739	0.000	0.454	1.010	1.000	63.191	0.000	5	55.6
159389382	6196.944	1016.944	1045.877	0.000	0.477	1.005	1.000	38.642	0.000	8	44.4
159470302	16095.889	2002.222	1830.911	0.000	0.519	1.011	0.999	104.081	0.000	7	77.8
159713063	13551.444	1663.111	1511.823	0.000	0.520	1.007	0.999	53.440	0.000	4	44.4
159733294	12529.222	1731.111	1754.594	0.000	0.498	1.008	0.999	58.365	0.000	5	55.6
160744799	1895.000	507.889	878.187	0.001	0.365	1.014	1.000	317.708	0.000	5	55.6
160825720	1629.500	535.444	844.746	0.001	0.401	1.001	1.000	18.569	0.017	2	11.1
160866180	1652.063	522.188	907.849	0.001	0.372	1.008	1.000	53.076	0.000	6	37.5
160886410	1671.889	530.667	857.071	0.001	0.380	1.009	1.000	120.061	0.000	3	33.3
160906640	2121.111	637.000	1070.756	0.000	0.373	1.011	1.000	102.767	0.000	5	55.6
160947100	1937.056	673.944	1169.945	0.001	0.375	1.005	1.000	19.179	0.014	5	27.8
160967330	1926.778	557.778	862.549	0.001	0.403	1.008	0.999	18.270	0.011	2	22.2
160987560	1667.667	544.000	984.890	0.002	0.413	0.975	1.000	38.798	0.000	3	33.3
161007791	1714.250	538.375	859.668	0.001	0.387	1.005	1.000	52.695	0.000	2	25.0
161270782	1701.143	592.571	1037.819	0.001	0.370	1.005	1.000	44.611	0.000	2	28.6
161311242	1662.167	548.333	1011.752	0.001	0.353	1.018	0.998	80.675	0.000	3	50.0
161412393	1762.333	665.667	1168.713	0.001	0.361	1.012	0.998	14.640	0.023	1	33.3

161473083	2533.889	620.333	864.645	0.000	0.424	1.009	0.999	246.060	0.000	3	33.3
161554003	2830.778	572.111	688.811	0.000	0.454	1.007	0.999	44.120	0.000	3	33.3
178713055	6283.571	949.143	981.375	0.000	0.491	1.003	1.000	7.964	0.632	1	14.3
184349034	5673.000	1072.667	1263.413	0.000	0.456	1.009	0.999	19.131	0.024	2	66.7
208027353	4418.333	984.000	1123.994	0.000	0.472	1.001	1.000	29.221	0.001	2	22.2
246515023	700.222	402.667	1061.209	0.001	0.293	0.994	1.000	0.676	0.984	1	11.1
256789458	3455.750	782.000	1053.043	0.000	0.418	1.019	0.999	57.079	0.000	3	75.0
275382046	4123.000	824.000	898.306	0.000	0.479	1.008	0.998	13.888	0.085	1	33.3
289996019	8677.111	900.222	875.446	0.000	0.496	1.010	0.999	29034.593	0.000	6	33.3
295137534	1276.750	417.000	708.151	0.001	0.374	1.007	0.999	19.035	0.015	2	25.0
336497421	2149.333	574.000	862.561	0.000	0.403	1.011	0.999	135.396	0.000	5	55.6
368533040	1558.300	526.700	840.336	0.001	0.380	0.991	1.000	7.265	0.508	1	10.0
370027359	3413.111	654.333	764.599	0.000	0.455	1.011	0.999	57.941	0.000	4	44.4
370425937	2811.250	605.750	784.289	0.000	0.434	1.008	0.999	61.809	0.000	4	33.3
375450439	4165.941	944.706	1138.892	0.000	0.447	1.011	0.999	87.530	0.000	4	23.5
404239096	3334.000	801.000	985.977	0.000	0.448	1.004	1.000	17.032	0.048	3	33.3
414519462	2322.444	516.000	713.235	0.000	0.416	1.015	0.999	67.651	0.000	4	44.4
451588811	1955.429	559.143	838.012	0.001	0.398	1.016	0.999	52.567	0.000	4	57.1
465578759	7541.556	810.000	821.763	0.000	0.487	1.007	0.999	36.515	0.000	2	22.2
492786515	2983.111	566.667	695.474	0.000	0.448	1.007	1.000	45.177	0.000	2	22.2
508703490	3261.500	599.875	668.728	0.000	0.465	1.018	0.999	41.410	0.000	3	37.5
514014184	2001.000	486.000	649.836	0.001	0.425	1.020	0.999	9.763	0.202	1	50.0
516889361	7393.500	908.000	901.214	0.001	0.459	1.006	1.000	3.037	0.981	1	50.0
517810313	5252.333	786.444	738.736	0.000	0.529	0.999	1.000	20.380	0.016	2	22.2
553359145	5394.875	887.375	975.085	0.000	0.472	1.008	0.999	38.175	0.000	2	25.0
604812005	869.889	430.778	1046.581	0.001	0.294	1.005	1.000	28.435	0.000	4	44.4
612472597	4961.111	1081.667	1240.088	0.000	0.462	1.006	0.999	84.734	0.000	4	44.4
638754422	2470.500	654.500	1025.181	0.000	0.393	1.014	0.999	100.960	0.000	3	37.5
643185023	4219.500	746.500	983.962	0.000	0.416	1.029	0.998	110.416	0.000	5	83.3
650853796	2358.714	710.000	1154.538	0.000	0.386	1.005	1.000	27.383	0.001	2	28.6
658594300	4569.750	789.000	896.364	0.000	0.468	1.007	1.000	17.316	0.044	1	25.0
663835652	4831.125	931.875	1130.549	0.000	0.452	1.012	0.999	56.195	0.000	5	62.5
668248235	5892.222	891.000	874.474	0.000	0.503	1.001	1.000	30.366	0.001	1	11.1
682449369	4781.222	1045.889	1091.601	0.000	0.490	0.999	1.000	19.215	0.014	3	33.3
686765762	1876.333	511.222	808.928	0.001	0.386	1.009	1.000	58.830	0.000	7	38.9
737052003	7506.444	1024.889	948.336	0.000	0.520	1.006	0.999	28.188	0.001	2	22.2
763395383	5344.778	993.444	1134.419	0.000	0.467	1.003	1.000	19.638	0.020	4	44.4
763435843	8764.778	1086.111	1109.788	0.000	0.476	1.022	0.999	3269.063	0.000	3	33.3
763456073	5227.444	916.556	853.269	0.000	0.520	0.999	0.999	38.846	0.000	1	11.1
763476303	3753.375	648.125	682.338	0.000	0.487	1.008	0.999	24.611	0.006	3	37.5
763516763	4537.625	721.875	772.820	0.000	0.470	1.015	0.998	85.025	0.000	8	50.0
763536994	7038.760	955.760	969.690	0.000	0.483	1.011	0.999	102.981	0.000	14	56.0
763557224	4493.250	597.375	566.161	0.000	0.497	1.006	0.999	91.343	0.000	3	37.5
763597684	11727.688	1430.188	1282.945	0.000	0.524	1.009	0.998	139.888	0.000	6	37.5
763638144	4263.188	879.000	1078.183	0.000	0.434	1.010	1.000	29.412	0.001	6	37.5
763698834	11723.667	1325.600	1149.329	0.000	0.531	1.007	0.999	63.571	0.000	7	46.7
763719065	3512.176	707.412	784.369	0.000	0.470	1.004	1.000	21.865	0.009	3	17.6
763759525	4963.563	845.813	890.689	0.000	0.476	1.006	0.999	32.768	0.000	5	31.3
763820215	4436.706	690.059	796.394	0.000	0.451	1.016	0.999	224.582	0.000	10	58.8
763840445	5462.444	1020.222	1075.188	0.000	0.488	1.002	0.999	53.378	0.000	7	38.9
763860675	5048.667	882.111	894.629	0.000	0.494	1.002	0.999	39.010	0.000	8	44.4

763880905	5964.111	885.333	934.012	0.000	0.495	1.012	0.998	45.115	0.000	3	33.3
763901136	3756.133	885.533	1180.456	0.000	0.430	1.005	1.000	38.725	0.000	6	40.0
763921366	5017.111	1003.000	1106.457	0.000	0.472	1.011	0.999	39.406	0.000	3	33.3
763982056	6177.167	885.833	882.407	0.000	0.497	1.011	0.999	75.119	0.000	6	33.3
764002286	7165.813	1025.125	1021.006	0.000	0.493	1.012	0.999	64.687	0.000	5	31.3
764042746	3440.188	715.063	906.748	0.000	0.441	1.008	0.999	115.152	0.000	5	31.3
764062976	6430.294	965.412	949.583	0.000	0.499	1.011	0.998	115.514	0.000	9	52.9
764083206	7766.278	1200.111	1258.211	0.000	0.485	1.012	0.999	121.072	0.000	11	61.1
764143897	6710.444	1080.667	1060.366	0.000	0.500	1.004	0.999	49.210	0.000	5	27.8
764184357	4291.588	926.824	1082.941	0.000	0.463	1.006	0.999	60.470	0.000	4	23.5
764224817	6296.889	947.333	911.596	0.000	0.516	1.003	0.998	62.927	0.000	5	27.8
764245047	7102.625	753.000	639.499	0.000	0.532	1.008	0.995	69.281	0.000	4	50.0
764285508	5714.444	887.556	856.215	0.000	0.508	1.013	0.998	71.548	0.000	5	55.6
764305738	9509.389	1133.000	1132.335	0.000	0.488	1.005	0.999	68.525	0.000	5	27.8
764325968	7101.267	1052.133	983.104	0.000	0.515	1.006	0.999	41.503	0.000	5	33.3
764346198	3788.125	704.063	833.891	0.000	0.458	1.007	1.000	81.286	0.000	5	31.3
764366428	5016.125	693.000	678.610	0.000	0.492	1.022	0.998	48.530	0.000	4	50.0
764447348	4046.833	586.167	592.432	0.001	0.484	1.011	0.998	77.722	0.000	7	38.9
764467579	3893.063	832.125	1042.278	0.000	0.453	1.003	1.000	10.929	0.363	5	31.3
764487809	6073.471	847.000	863.752	0.000	0.491	1.008	0.999	49.448	0.000	7	41.2
764508039	4602.500	794.250	844.009	0.000	0.475	1.014	0.999	33.890	0.000	2	25.0
764588959	4764.444	775.556	762.151	0.000	0.502	1.005	0.999	21.731	0.010	2	22.2
764649650	4475.556	812.222	876.082	0.000	0.481	1.005	1.000	16.962	0.049	4	44.4
764669880	4973.941	1023.529	1244.771	0.000	0.452	1.006	1.000	118.447	0.000	6	35.3
764750800	4764.333	1023.222	1250.317	0.000	0.451	1.007	0.999	32.289	0.000	3	33.3
764811490	7730.556	1023.444	984.893	0.000	0.511	1.009	0.999	29.540	0.002	2	22.2
764831721	4387.444	982.667	1270.348	0.000	0.432	1.006	1.000	21.847	0.016	2	22.2
764872181	4315.333	836.667	854.258	0.000	0.496	1.001	1.000	11.973	0.152	0	0.0
764892411	4008.333	879.667	999.047	0.000	0.467	1.006	0.998	34.038	0.000	2	22.2
764953101	4620.556	940.889	1163.971	0.000	0.443	1.012	1.000	63.436	0.000	4	44.4
765013792	3762.889	886.444	1066.650	0.000	0.456	1.002	1.000	13.856	0.128	1	11.1
765034022	4026.667	889.333	1008.218	0.000	0.466	1.004	0.999	24.521	0.002	2	22.2
765074482	3725.929	807.357	926.540	0.000	0.465	1.003	0.999	36.450	0.000	4	28.6
765094712	4709.143	816.786	915.605	0.000	0.465	1.010	0.999	55.685	0.000	5	35.7
765155402	4269.333	700.444	700.873	0.000	0.503	1.006	0.999	16.693	0.081	3	33.3
765195863	3266.000	711.778	830.537	0.000	0.458	1.009	0.998	35.896	0.000	2	22.2
765216093	4371.778	825.222	844.647	0.000	0.499	1.002	0.999	22.019	0.005	2	22.2
765256553	4984.500	1016.625	1123.483	0.000	0.469	1.009	0.999	35.998	0.000	3	37.5
765276783	4127.556	738.222	836.062	0.000	0.463	1.015	0.999	44.473	0.000	2	22.2
765317243	3326.875	683.500	807.666	0.000	0.453	1.007	0.999	11.566	0.172	2	25.0
765337473	3573.444	770.889	916.053	0.000	0.462	1.007	0.999	22.011	0.005	4	44.4
765377934	3901.000	785.833	1007.969	0.000	0.428	1.024	0.995	98.911	0.000	4	66.7
765560005	5103.444	1067.000	1228.285	0.000	0.462	1.003	1.000	7.575	0.577	3	33.3
809635352	2087.889	537.000	793.549	0.001	0.413	1.013	0.999	60.583	0.000	4	44.4
863126187	8190.667	986.556	908.475	0.000	0.498	1.002	0.999	33.606	0.000	5	55.6
892969023	1055.333	468.778	893.022	0.001	0.362	0.992	1.000	19.002	0.008	0	0.0
937495960	1887.667	543.222	817.130	0.001	0.400	1.010	1.000	153.203	0.000	2	22.2
953045535	5073.000	771.889	796.302	0.000	0.487	1.015	0.998	59.208	0.000	6	66.7
970836795	2564.444	680.667	1051.845	0.000	0.389	1.018	0.999	76.988	0.000	5	55.6
Passing Rate									13(8.9%)	4	38.5

**J*: the average number of individuals per niche (local community) in each metacommunity, *S*: the average species

numbers per niche (local community) in each metacommunity, θ: the average fundamental biodiversity parameter per niche (local community) in each metacommunity, m: the average of the migration coefficients, x: the average of the birth to death ratio, $\boldsymbol{\gamma}$: the average of the migration rate, R^2: the goodness-of-fitting index, χ^2-value: the χ^2-value of chi-squared test for observed value against predicted value, p-value for the χ^2-test; when p-value > 0.05, the metacommunity satisfies the NNH model. The last two columns are the number and percentage of local communities (niches) that passed the local neutrality test. Note that $R^2=1$ resulted from approximation with four effective digits only (*e.g*., 0.99995, exact 1 is nearly impossible to achieve).

Table S10. Test results of fitting NNH (multi-site neutral) model to HMP-skin datasets (*Sites*≥2, *Subjects*=159)

Group ID	J	S	θ	m	x	γ	R^2	χ^2	p-value	N_{pass}	$\%_{(pass)}$
132902142	1694.000	246.333	430.888	0.002	0.317	1.082	0.997	341.272	0.000	3	100.0
147406386	4417.500	491.250	642.793	0.000	0.417	1.035	0.996	177.070	0.000	4	100.0
158013734	3494.500	501.750	784.223	0.000	0.387	1.035	0.999	299.040	0.000	4	100.0
158114885	4684.750	849.000	1177.270	0.000	0.407	1.023	0.998	121.161	0.000	3	75.0
158155345	6280.750	749.000	862.711	0.000	0.461	1.013	0.998	49.826	0.000	2	50.0
158216035	6848.667	992.000	1258.157	0.000	0.423	1.023	0.998	97.433	0.000	2	66.7
158236265	5747.000	630.333	710.472	0.000	0.455	1.049	0.994	184.691	0.000	3	100.0
158276726	3408.000	423.500	598.595	0.000	0.393	1.033	0.998	1270.949	0.000	3	75.0
158398106	4842.250	964.250	1484.624	0.000	0.383	1.028	0.998	197.197	0.000	4	100.0
158418336	4720.600	1131.200	1757.249	0.000	0.386	1.015	0.999	118.688	0.000	4	80.0
158438567	2031.500	489.500	842.411	0.004	0.310	1.012	0.999	28.676	0.000	2	50.0
158458797	4050.000	624.750	899.663	0.000	0.407	1.027	0.997	191.257	0.000	3	75.0
158479027	2583.000	666.500	1251.629	0.000	0.336	1.027	0.999	141.693	0.000	2	100.0
158721788	2966.500	956.000	1525.392	0.000	0.385	1.006	1.000	10.368	0.169	0	0.0
158742018	3703.667	725.333	1177.148	0.001	0.368	1.027	0.998	356.807	0.000	5	83.3
158802708	9045.000	1220.500	1227.087	0.000	0.499	1.018	1.000	17.928	0.056	2	50.0
158822939	12788.333	2122.333	2663.000	0.000	0.429	1.025	0.999	95.592	0.000	2	66.7
158883629	4401.800	816.000	1294.137	0.000	0.376	1.023	0.998	165.122	0.000	4	80.0
158924089	13159.500	1330.250	1417.203	0.002	0.393	1.025	0.999	87.511	0.000	3	75.0
158944319	3164.500	968.000	1541.486	0.000	0.385	1.006	1.000	19.504	0.007	1	50.0
158964549	3413.429	613.714	816.470	0.000	0.425	1.022	0.999	419.677	0.000	5	71.4
159005010	3256.000	330.667	303.379	0.002	0.429	1.054	0.993	28.281	0.002	2	66.7
159025240	3284.500	1065.000	1806.072	0.000	0.372	1.007	1.000	17.051	0.017	1	50.0
159085930	6284.500	623.750	725.910	0.002	0.392	1.026	0.995	38.938	0.000	3	75.0
159146620	6123.000	1031.000	1357.457	0.000	0.414	1.022	0.999	79.973	0.000	5	100.0
159166850	12718.250	1861.000	2370.860	0.000	0.417	1.028	0.999	389.036	0.000	8	100.0
159227541	6781.000	1024.500	1115.056	0.000	0.459	1.024	0.992	145.754	0.000	4	100.0
159247771	5526.333	601.667	657.387	0.001	0.452	0.998	0.998	41.678	0.000	2	66.7
159389382	5935.750	894.500	1205.691	0.000	0.411	1.029	0.998	240.729	0.000	3	75.0
159470302	19284.000	1307.250	1362.367	0.001	0.443	1.006	1.000	36.315	0.001	1	25.0
159713063	11679.500	1553.750	2127.160	0.000	0.403	1.039	0.996	488.140	0.000	4	100.0
159733294	8860.250	953.250	1261.705	0.000	0.415	1.039	0.995	329.218	0.000	4	100.0
160744799	4018.000	485.000	791.461	0.000	0.362	1.044	0.995	158.984	0.000	1	100.0
160825720	2834.000	949.600	1596.851	0.000	0.369	1.009	0.999	17.979	0.021	1	20.0
160866180	1973.600	469.400	865.892	0.001	0.353	1.032	0.999	495.717	0.000	2	40.0
160886410	2596.000	675.333	987.977	0.006	0.411	0.984	0.999	18.060	0.021	0	0.0
160906640	3113.000	1026.500	1841.709	0.000	0.355	1.009	0.999	21.131	0.007	1	50.0
160947100	2487.750	832.250	1481.656	0.001	0.354	1.009	1.000	14.966	0.060	0	0.0
160967330	2096.750	834.250	1313.915	0.001	0.392	0.993	1.000	4.466	0.614	0	0.0
160987560	1794.500	804.500	1591.265	0.001	0.326	0.996	1.000	2.577	0.765	0	0.0
161007791	1870.250	679.250	1164.615	0.001	0.417	0.995	1.000	4.731	0.579	0	0.0
161270782	1734.500	740.500	1308.020	0.001	0.363	0.998	1.000	2.165	0.904	0	0.0
161311242	1795.250	678.250	1231.361	0.001	0.353	1.009	1.000	21.277	0.003	0	0.0
161331472	1413.500	578.500	993.982	0.001	0.373	1.001	0.999	6.784	0.341	0	0.0
161412393	4307.750	929.000	1542.509	0.000	0.363	1.034	0.998	226.299	0.000	4	100.0
161473083	1526.667	316.667	730.036	0.001	0.272	1.067	0.998	886.804	0.000	3	100.0
161554003	2914.000	636.667	1020.027	0.000	0.375	1.028	0.998	116.992	0.000	3	100.0
178713055	1684.000	236.000	550.925	0.007	0.232	1.036	0.998	67.671	0.000	3	100.0
184349034	4079.750	658.500	984.624	0.000	0.389	1.041	0.996	238.523	0.000	3	75.0
208027353	3532.000	484.250	722.056	0.000	0.381	1.063	0.995	315.566	0.000	4	100.0
246515023	2634.800	720.400	1224.241	0.001	0.359	1.022	1.000	43.891	0.000	3	60.0
256789458	3728.000	565.250	900.062	0.001	0.350	1.046	0.998	224.358	0.000	4	100.0
275382046	4106.333	405.333	714.506	0.001	0.346	1.039	0.998	784.115	0.000	3	100.0
295137534	4752.500	725.500	818.144	0.000	0.467	1.014	0.996	17.939	0.036	1	50.0
336497421	5926.500	1532.000	1951.757	0.000	0.439	1.001	1.000	7.556	0.478	0	0.0
338793263	4087.333	648.000	1053.193	0.001	0.361	1.033	0.997	251.070	0.000	3	100.0
355657046	5015.333	836.333	1066.293	0.000	0.432	1.016	0.999	50.638	0.000	2	66.7
368533040	3519.250	825.250	1338.742	0.000	0.373	1.024	0.998	156.225	0.000	4	100.0
370027359	6722.000	983.750	1184.610	0.000	0.451	1.012	1.000	34.122	0.000	3	75.0
370425937	3806.250	903.500	1545.620	0.000	0.364	1.021	0.999	119.085	0.000	4	100.0
375450439	2878.250	461.000	714.604	0.002	0.338	1.042	0.996	171.993	0.000	3	75.0

404239096	4544.000	837.000	1183.468	0.000	0.401	1.031	0.997	96.852	0.000	2	100.0
414519462	6015.600	1213.200	1733.955	0.000	0.419	1.051	0.998	162.674	0.000	5	100.0
451588811	10542.500	1842.500	2238.226	0.000	0.440	1.024	0.998	91.835	0.000	2	100.0
465578759	3380.000	542.750	932.635	0.001	0.301	1.092	0.997	275.233	0.000	4	100.0
492786515	4178.333	747.667	1040.916	0.000	0.402	1.027	0.999	70.622	0.000	2	66.7
508703490	4562.500	707.500	1107.349	0.000	0.374	1.038	0.997	157.689	0.000	2	100.0
516889361	2607.750	604.500	1087.123	0.001	0.332	1.024	0.998	138.202	0.000	3	75.0
517810313	6019.500	868.250	1346.945	0.000	0.380	1.037	0.998	416.861	0.000	4	100.0
550534656	4862.000	727.250	1123.757	0.000	0.373	1.040	0.997	341.944	0.000	3	75.0
553359145	3862.250	615.500	1025.941	0.001	0.357	1.041	0.997	295.379	0.000	4	100.0
604812005	1719.667	721.667	1281.451	0.001	0.359	1.000	1.000	5.281	0.508	0	0.0
612472597	2097.500	514.000	834.438	0.001	0.355	1.038	0.998	98.929	0.000	3	75.0
638754422	2929.500	692.500	1274.995	0.000	0.343	1.026	0.999	239.482	0.000	4	100.0
643185023	1063.333	78.333	137.258	0.002	0.308	1.165	0.967	489.596	0.000	3	100.0
650853796	3330.000	682.333	1131.286	0.000	0.356	1.024	0.999	326.345	0.000	2	66.7
658594300	968.000	136.667	253.969	0.008	0.440	0.967	0.997	101.863	0.000	1	33.3
668248235	5458.250	953.000	1399.231	0.000	0.400	1.024	0.999	192.263	0.000	4	100.0
675950834	2489.333	634.667	899.561	0.002	0.374	1.061	0.999	21.394	0.006	2	66.7
682102541	5614.000	537.667	829.774	0.000	0.381	1.042	0.998	808.144	0.000	3	100.0
682449369	1877.750	307.500	504.174	0.001	0.323	1.065	0.997	163.095	0.000	4	100.0
686765762	3659.625	832.250	1343.497	0.000	0.372	1.015	0.999	112.085	0.000	4	50.0
706846339	3546.500	939.500	1369.266	0.000	0.403	1.008	1.000	13.088	0.159	0	0.0
737052003	4480.000	797.500	974.587	0.000	0.453	1.002	0.999	14.726	0.099	0	0.0
739574095	3072.000	403.500	776.640	0.000	0.317	1.061	0.995	754.960	0.000	2	100.0
763395383	4395.667	942.333	1271.693	0.000	0.418	1.019	0.998	51.138	0.000	1	33.3
763435843	5728.000	954.000	1044.006	0.000	0.475	1.006	0.997	19.169	0.024	0	0.0
763456073	2289.500	571.000	1045.765	0.000	0.351	1.004	1.000	60.425	0.000	2	100.0
763476303	7152.750	578.000	856.607	0.000	0.380	1.049	0.996	770.502	0.000	4	100.0
763516763	5107.000	650.333	910.826	0.000	0.431	1.034	0.995	658.746	0.000	5	83.3
763536994	8757.778	803.444	974.071	0.000	0.417	1.048	0.996	462.769	0.000	9	100.0
763597684	14811.167	1660.500	1843.332	0.000	0.458	1.025	0.998	188.433	0.000	5	83.3
763638144	1545.000	149.667	201.545	0.001	0.428	1.066	0.984	145.799	0.000	3	100.0
763678604	8816.714	751.286	856.269	0.000	0.447	1.039	0.994	400.068	0.000	7	100.0
763698834	13525.857	1230.571	1340.170	0.000	0.461	1.029	0.998	221.657	0.000	7	100.0
763719065	2409.000	232.200	358.117	0.001	0.363	1.073	0.994	557.307	0.000	5	100.0
763759525	7119.000	769.333	969.352	0.000	0.436	1.029	0.998	301.431	0.000	5	83.3
763820215	2868.750	307.250	413.829	0.000	0.474	1.013	0.998	64.552	0.000	2	50.0
763840445	5201.500	472.667	522.293	0.000	0.471	1.038	0.998	122.556	0.000	4	66.7
763860675	6561.200	625.600	796.115	0.000	0.429	1.026	0.998	206.126	0.000	5	100.0
763880905	8822.250	703.625	862.982	0.000	0.426	1.057	0.995	701.819	0.000	8	100.0
763901136	5127.857	316.286	336.853	0.000	0.442	1.066	0.990	286.172	0.000	7	100.0
763921366	6307.750	539.500	571.562	0.000	0.481	1.020	0.999	44.694	0.000	2	50.0
763982056	8054.500	836.000	1034.788	0.000	0.435	1.030	0.998	255.575	0.000	4	66.7
764002286	8341.625	625.875	901.764	0.000	0.386	1.059	0.994	1170.799	0.000	8	100.0
764042746	4901.333	310.333	385.333	0.001	0.416	1.041	0.997	35.306	0.000	2	66.7
764062976	1548.250	259.250	299.514	0.001	0.488	1.025	0.998	31.858	0.000	1	25.0
764083206	8171.143	605.571	724.332	0.000	0.428	1.076	0.995	644.721	0.000	7	100.0
764143897	1725.500	336.000	559.512	0.001	0.354	1.040	0.998	194.564	0.000	5	83.3
764184357	1753.286	403.143	701.678	0.001	0.346	1.039	0.997	178.921	0.000	6	85.7
764224817	2766.667	638.333	948.922	0.000	0.383	1.024	0.998	92.399	0.000	6	100.0
764245047	1622.000	275.333	322.680	0.001	0.446	1.036	0.993	51.350	0.000	2	66.7
764305738	25683.875	1577.875	1816.033	0.000	0.408	1.026	0.999	48.570	0.000	7	87.5
764325968	2964.500	719.500	1061.145	0.000	0.394	1.013	1.000	47.241	0.000	2	33.3
764346198	2346.250	511.500	810.058	0.001	0.375	1.021	0.999	71.613	0.000	3	75.0
764366428	810.667	242.333	555.821	0.002	0.285	1.038	0.998	57.270	0.000	3	100.0
764447348	2604.500	627.000	953.892	0.000	0.388	1.018	0.999	54.066	0.000	2	50.0
764467579	1682.333	157.333	204.989	0.001	0.418	1.055	0.993	158.970	0.000	3	100.0
764487809	2074.667	496.833	817.516	0.001	0.359	1.030	0.999	103.640	0.000	4	66.7
764508039	2025.750	487.250	813.714	0.001	0.372	1.021	0.998	94.682	0.000	2	50.0
764588959	606.250	189.250	445.715	0.002	0.301	1.050	0.994	96.146	0.000	4	100.0
764649650	2006.500	485.250	796.704	0.001	0.366	1.024	0.998	70.223	0.000	3	75.0
764669880	2048.250	429.000	688.272	0.001	0.361	1.032	0.998	241.330	0.000	8	100.0
764710340	1315.250	220.000	291.598	0.001	0.420	1.025	0.996	54.915	0.000	3	75.0
764750800	6788.667	1028.000	1462.871	0.000	0.401	1.030	0.998	192.429	0.000	3	100.0
764811490	3524.667	707.000	1258.666	0.000	0.339	1.039	0.998	1062.358	0.000	3	100.0

764831721	4301.500	573.500	662.398	0.000	0.428	1.084	0.972	288.967	0.000	2	100.0
764872181	6134.667	605.000	1098.268	0.000	0.334	1.059	0.997	1417.129	0.000	3	100.0
764892411	1788.500	483.500	820.260	0.001	0.363	1.017	0.999	65.855	0.000	3	75.0
765013792	1674.667	336.333	615.460	0.001	0.334	1.053	0.995	237.602	0.000	3	100.0
765034022	1164.500	257.750	502.335	0.001	0.321	1.051	0.996	303.482	0.000	4	100.0
765074482	2509.000	560.250	899.946	0.000	0.374	1.019	0.999	90.730	0.000	4	100.0
765094712	3432.500	849.625	1362.686	0.000	0.369	1.031	0.996	305.975	0.000	8	100.0
765135172	2171.333	470.667	749.722	0.001	0.366	1.032	0.997	57.260	0.000	3	100.0
765155402	3542.200	687.400	957.399	0.000	0.408	1.027	0.998	122.311	0.000	4	80.0
765195863	4156.750	736.250	956.994	0.000	0.416	1.039	0.998	114.492	0.000	3	75.0
765216093	5212.250	732.500	920.648	0.000	0.434	1.021	0.999	98.294	0.000	4	100.0
765256553	5242.000	313.000	361.144	0.000	0.429	1.089	0.984	303.829	0.000	2	100.0
765276783	5405.000	773.750	1085.394	0.000	0.403	1.032	0.998	174.925	0.000	4	100.0
765317243	6064.500	535.000	640.994	0.000	0.456	1.026	0.997	60.191	0.000	2	100.0
765337473	4984.000	898.250	1153.631	0.000	0.431	1.014	1.000	39.517	0.000	2	50.0
765377934	6565.750	1061.000	1348.195	0.000	0.424	1.028	0.998	107.727	0.000	3	75.0
765398164	5593.500	526.000	456.615	0.000	0.539	1.008	0.999	9.215	0.418	0	0.0
765519544	5965.750	933.500	1164.142	0.000	0.433	1.022	0.998	86.139	0.000	4	100.0
765539774	3651.667	526.667	688.829	0.000	0.421	1.040	0.993	130.102	0.000	3	100.0
765560005	5433.833	826.500	1088.018	0.000	0.412	1.034	0.998	154.197	0.000	6	100.0
765620695	4423.500	347.500	445.176	0.000	0.408	1.070	0.989	243.669	0.000	2	100.0
765640925	7139.000	1043.500	1327.182	0.000	0.434	1.012	1.000	49.039	0.000	2	100.0
765661155	6054.000	641.500	897.076	0.000	0.404	1.029	0.999	145.822	0.000	2	100.0
765701615	8096.250	1018.250	1194.786	0.000	0.447	1.016	1.000	51.824	0.000	3	75.0
809635352	7933.250	1528.750	1855.781	0.000	0.446	1.013	0.999	42.290	0.000	2	50.0
823052294	2376.750	554.750	912.080	0.004	0.305	1.017	0.998	24.820	0.003	2	50.0
857980665	6727.333	773.000	1077.377	0.002	0.363	1.047	0.992	650.895	0.000	2	66.7
861967750	3600.000	654.500	862.361	0.000	0.419	1.029	0.998	103.588	0.000	2	50.0
863126187	5491.667	894.000	1411.091	0.000	0.374	1.035	0.997	285.500	0.000	3	100.0
875002022	1474.000	317.500	492.451	0.001	0.378	1.058	0.987	64.998	0.000	2	100.0
892969023	3888.500	685.250	1066.076	0.000	0.388	1.017	1.000	143.682	0.000	3	75.0
953045535	2720.500	430.750	640.394	0.001	0.367	1.052	0.996	182.713	0.000	4	100.0
970836795	3041.333	781.667	1331.485	0.000	0.363	1.017	0.999	109.900	0.000	3	100.0
Passing Rate									13(8.2%)	3	74.9

*J: the average number of individuals per niche (local community) in each metacommunity, S: the average species numbers per niche (local community) in each metacommunity, θ: the average fundamental biodiversity parameter per niche (local community) in each metacommunity, m: the average of the migration coefficients, x: the average of the birth to death ratio, $\boldsymbol{\gamma}$: the average of the migration rate, R^2: the goodness-of-fitting index, χ^2-value: the χ^2-value of chi-squared test for observed value against predicted value, p-value for the χ^2-test; when p-value > 0.05, the metacommunity satisfies the NNH model. The last two columns are the number and percentage of local communities (niches) that passed the local neutrality test. Note that $R^2=1$ resulted from approximation with four effective digits only (*e.g.*, 0.99995, exact 1 is nearly impossible to achieve).

Table S11. Test results of fitting NNH (multi-site neutral) model to HMP-vaginal datasets (*Sites*≥2, Subjects=72)

Group ID	J	S	θ	m	x	γ	R^2	χ^2	p-value	N_{pass}	$\%_{(pass)}$
158013734	2830.333	125.333	48.946	0.000	0.748	0.943	0.928	13.375	0.100	0	0.0
158114885	3985.500	127.000	54.974	0.000	0.724	0.954	0.970	6.947	0.730	0	0.0
158236265	5756.000	280.667	231.480	0.000	0.541	1.021	0.995	28.701	0.001	1	33.3
158276726	2886.333	222.667	193.060	0.000	0.520	1.045	0.994	31.709	0.000	2	66.7
158458797	4796.833	262.167	154.951	0.000	0.640	0.986	0.997	12.928	0.228	0	0.0
158742018	10800.000	269.167	108.909	0.000	0.730	0.967	0.995	23.521	0.024	2	33.3
158883629	15138.167	219.000	102.159	0.000	0.684	0.994	0.966	39.264	0.000	2	33.3
158944319	9759.333	263.667	213.702	0.000	0.546	1.015	0.996	41.893	0.000	1	33.3
158984779	8897.667	293.000	152.242	0.000	0.689	0.962	0.989	15.189	0.125	0	0.0
159005010	11763.333	639.000	525.658	0.000	0.546	0.992	0.998	14.876	0.188	0	0.0
159025240	8325.333	263.333	127.599	0.000	0.689	0.971	0.979	29.656	0.002	0	0.0
159085930	21798.333	472.000	240.895	0.000	0.672	0.980	0.994	24.070	0.012	0	0.0
159227541	5984.333	175.333	115.803	0.000	0.611	1.003	0.985	48.598	0.000	3	50.0
159247771	7696.000	181.333	103.299	0.000	0.640	1.016	0.961	34.597	0.000	2	66.7
159470302	13848.667	479.000	343.022	0.000	0.574	1.018	0.996	31.635	0.002	0	0.0
159713063	13230.333	254.333	133.196	0.000	0.662	1.002	0.965	40.029	0.000	1	33.3
159733294	10860.667	377.000	260.803	0.000	0.583	1.009	0.997	12.450	0.331	1	33.3
160744799	2692.333	196.333	131.356	0.000	0.612	0.986	0.996	8.868	0.354	0	0.0
160866180	2938.500	323.333	229.373	0.000	0.590	0.985	0.995	33.543	0.000	1	16.7
161007791	5663.500	455.000	263.234	0.000	0.654	0.961	0.988	34.514	0.000	1	50.0
161473083	3394.333	184.333	121.527	0.000	0.631	0.999	0.995	10.128	0.340	1	33.3
178713055	4504.000	234.000	154.007	0.000	0.607	0.993	0.983	9.565	0.297	0	0.0
208027353	5623.000	210.500	132.119	0.000	0.610	1.009	0.996	17.171	0.071	0	0.0
246515023	2108.667	210.333	108.122	0.000	0.684	0.954	0.953	41.415	0.000	3	100.0
256789458	5387.000	350.000	247.265	0.000	0.593	0.986	0.990	6.426	0.600	0	0.0
370425937	4181.333	172.667	84.002	0.000	0.686	0.975	0.976	12.019	0.212	0	0.0
375450439	5386.500	329.500	255.026	0.000	0.582	0.999	1.000	9.855	0.543	0	0.0
414519462	4403.333	139.000	109.061	0.000	0.537	1.050	0.991	109.117	0.000	3	100.0
451588811	6279.000	386.000	431.825	0.000	0.473	1.012	0.999	57.331	0.000	1	33.3
465578759	6748.667	200.667	124.889	0.000	0.638	0.994	0.992	22.329	0.022	2	66.7
508703490	5769.500	205.000	113.060	0.000	0.654	0.985	0.980	10.708	0.381	0	0.0
553359145	5950.000	144.000	71.254	0.000	0.688	0.974	0.995	6.680	0.755	0	0.0
561079058	6000.000	209.500	244.490	0.000	0.443	1.051	0.992	302.078	0.000	2	100.0
612472597	7223.000	301.333	260.251	0.000	0.528	1.019	0.998	37.887	0.000	1	33.3
638754422	3877.333	386.667	345.300	0.000	0.522	1.012	0.998	18.222	0.033	1	33.3
650853796	5931.000	169.000	100.154	0.000	0.668	0.979	0.992	7.220	0.705	0	0.0
663835652	5548.000	353.667	277.146	0.000	0.561	1.000	0.997	11.674	0.232	0	0.0
682449369	5360.333	250.667	110.013	0.000	0.713	0.964	0.993	7.232	0.613	0	0.0
737052003	9024.000	327.333	234.069	0.000	0.578	1.012	0.994	29.693	0.003	1	33.3
739574095	15251.000	415.000	358.128	0.000	0.527	1.023	0.997	83.696	0.000	2	100.0
763456073	5515.667	188.333	105.099	0.000	0.663	0.978	0.981	13.394	0.268	0	0.0
763557224	6775.000	225.667	226.563	0.000	0.480	1.042	0.993	180.404	0.000	3	100.0
763638144	4035.600	187.200	108.542	0.000	0.639	0.987	0.998	24.607	0.006	1	20.0
763698834	15299.667	401.167	325.023	0.000	0.551	1.016	0.996	104.770	0.000	4	66.7
763719065	2953.833	288.667	219.246	0.000	0.562	0.991	0.995	126.809	0.000	5	83.3
763759525	7822.333	373.500	316.173	0.000	0.529	1.024	0.997	66.986	0.000	3	50.0
763820215	7031.833	182.000	118.579	0.000	0.608	0.996	0.997	44.482	0.000	0	0.0
763840445	8469.833	189.167	81.739	0.000	0.710	0.977	0.986	11.845	0.375	1	16.7
763901136	4582.500	330.167	239.546	0.000	0.581	0.992	0.999	39.163	0.000	4	66.7
763921366	7591.667	217.333	177.966	0.000	0.537	1.027	0.993	69.110	0.000	3	100.0
764042746	5170.167	256.000	159.129	0.000	0.610	1.001	0.997	84.991	0.000	3	50.0
764062976	6589.833	159.333	76.126	0.000	0.679	0.986	0.992	8.414	0.588	0	0.0
764143897	9334.167	325.000	176.753	0.000	0.653	0.988	0.998	11.459	0.323	0	0.0
764184357	7417.500	302.833	188.753	0.000	0.616	1.006	0.997	20.576	0.024	2	33.3
764245047	7536.000	196.000	210.129	0.000	0.445	1.087	0.988	630.742	0.000	3	100.0
764346198	5269.667	305.667	224.976	0.000	0.567	1.005	0.995	187.243	0.000	5	83.3
764366428	7117.333	183.000	162.586	0.000	0.475	1.119	0.980	555.243	0.000	3	100.0
764467579	6914.000	314.200	272.763	0.000	0.508	1.038	0.999	210.625	0.000	5	100.0
764588959	5912.000	163.500	73.182	0.000	0.701	0.979	0.972	12.109	0.278	0	0.0
764649650	4311.333	262.667	263.232	0.000	0.487	1.025	0.996	97.638	0.000	3	100.0
764710340	4962.667	137.333	73.517	0.000	0.648	1.007	0.947	43.168	0.000	2	66.7

764811490	8479.000	427.000	294.098	0.000	0.594	0.999	0.995	14.411	0.211	0	0.0
765013792	4748.000	227.000	225.705	0.000	0.520	1.025	0.995	23.159	0.010	1	33.3
765034022	4475.500	173.000	118.765	0.000	0.570	1.045	0.944	24.037	0.008	1	50.0
765195863	5772.000	264.000	266.795	0.000	0.484	1.053	0.995	130.891	0.000	2	66.7
765256553	5461.667	315.667	284.380	0.000	0.517	1.017	0.998	43.776	0.000	2	66.7
765317243	6290.667	235.000	187.561	0.000	0.548	1.020	0.998	34.421	0.000	1	33.3
765337473	3409.667	81.667	61.359	0.000	0.572	1.017	0.985	49.107	0.000	1	33.3
809635352	11829.333	227.000	107.221	0.000	0.652	1.009	0.980	12.236	0.346	1	33.3
892969023	1944.333	407.667	375.924	0.001	0.533	0.976	0.994	26.268	0.001	1	33.3
953045535	6904.500	277.500	190.694	0.000	0.593	1.001	0.997	8.024	0.626	0	0.0
970836795	6533.000	263.000	151.913	0.000	0.641	0.986	0.988	8.853	0.451	0	0.0
Passing Rate									27(37.5%)	1	35.7

**J*: the average number of individuals per niche (local community) in each metacommunity, *S*: the average species numbers per niche (local community) in each metacommunity, θ: the average fundamental biodiversity parameter per niche (local community) in each metacommunity, *m*: the average of the migration coefficients, *x*: the average of the birth to death ratio, γ: the average of the migration rate, R^2: the goodness-of-fitting index, χ^2-value: the χ^2-value of chi-squared test for observed value against predicted value, *p*-value for the χ^2-test; when *p*-value > 0.05, the metacommunity satisfies the NNH model. The last two columns are the number and percentage of local communities (niches) that passed the local neutrality test. Note that $R^2=1$ resulted from approximation with four effective digits only (*e.g.*, 0.99995, exact 1 is nearly impossible to achieve). See Fig 3 for an example of fitting the NNH model.

Table S12. Test results of fitting NNH (multi-site neutral) model to HMP-whole datasets (*Sites*≥5, Subjects=172)

Group ID	J	S	θ	m	x	γ	R^2	χ^2	p-value	N_{pass}	$\%_{(pass)}$
103092734	1632.500	286.500	465.152	0.001	0.344	1.084	0.994	122.172	0.000	2	100.0
132902142	4662.500	749.600	841.491	0.001	0.434	1.027	0.999	87.094	0.000	7	70.0
147406386	5012.067	768.000	865.055	0.000	0.463	1.015	0.999	142.792	0.000	8	53.3
158013734	3174.722	594.611	783.723	0.000	0.480	1.004	1.000	43.099	0.000	8	44.4
158114885	4149.941	803.353	1001.944	0.000	0.476	1.004	0.999	62.171	0.000	6	35.3
158155345	4545.067	768.533	860.602	0.000	0.472	1.008	0.999	86.274	0.000	4	26.7
158216035	5923.857	954.714	1029.645	0.000	0.477	1.011	0.999	83.099	0.000	4	28.6
158236265	5080.313	731.625	883.325	0.000	0.461	1.027	0.998	254.209	0.000	9	56.3
158276726	5003.889	627.500	663.117	0.000	0.476	1.021	0.999	1223.559	0.000	9	50.0
158398106	5190.538	796.846	893.917	0.000	0.474	1.010	0.999	363.102	0.000	13	50.0
158418336	5285.923	1000.654	1240.645	0.000	0.439	1.014	0.999	177.178	0.000	17	65.4
158438567	7518.080	966.200	969.699	0.001	0.476	1.009	0.999	111.358	0.000	15	60.0
158458797	5930.750	732.344	741.720	0.000	0.502	1.012	0.999	164.020	0.000	9	28.1
158479027	4886.385	951.923	1197.572	0.000	0.437	1.014	0.998	96.019	0.000	7	53.8
158499257	4689.250	769.667	955.682	0.000	0.424	1.027	0.998	83.054	0.000	7	58.3
158721788	4941.769	871.385	1138.813	0.000	0.436	1.021	0.999	133.169	0.000	7	53.8
158742018	6215.939	812.788	940.138	0.000	0.494	1.005	0.999	168.904	0.000	22	66.7
158802708	7700.400	1126.600	1221.458	0.000	0.484	1.007	1.000	42.557	0.000	6	40.0
158822939	13452.571	1852.857	1912.546	0.000	0.497	1.004	0.999	65.929	0.000	8	57.1
158883629	8427.545	837.424	954.826	0.000	0.509	1.007	0.999	140.915	0.000	17	51.5
158924089	14571.400	1657.333	1631.609	0.001	0.475	1.008	1.000	28.183	0.003	7	46.7
158944319	7475.000	878.563	1004.189	0.000	0.478	1.014	0.999	125.538	0.000	8	50.0
158964549	8952.517	1053.759	1043.060	0.000	0.492	1.009	0.999	111.651	0.000	16	55.2
158984779	6832.769	666.769	697.027	0.000	0.529	1.007	0.998	76.755	0.000	7	53.8
159005010	8987.529	1155.588	1174.353	0.000	0.488	1.013	1.000	40.513	0.000	7	41.2
159025240	5965.938	772.313	973.289	0.000	0.494	1.002	0.999	60.744	0.000	7	43.8
159085930	10745.833	1077.722	1069.847	0.000	0.503	1.006	1.000	37.860	0.000	9	50.0
159146620	10382.037	1275.852	1323.635	0.000	0.486	1.013	0.999	172.298	0.000	16	59.3
159166850	12403.800	1558.200	1661.682	0.000	0.476	1.016	0.999	361.339	0.000	22	73.3
159227541	6832.903	921.677	1008.392	0.000	0.498	1.011	0.999	140.075	0.000	15	48.4
159247771	6659.412	856.412	963.745	0.000	0.491	1.009	1.000	41.668	0.000	10	58.8
159389382	5823.346	925.923	991.308	0.000	0.472	1.008	1.000	57.586	0.000	13	50.0
159470302	16209.833	1449.556	1358.824	0.000	0.506	1.013	0.999	189.329	0.000	10	55.6
159713063	13532.667	1363.056	1395.593	0.000	0.520	1.013	0.998	206.358	0.000	10	55.6
159733294	11694.111	1312.111	1360.807	0.000	0.500	1.013	0.999	148.912	0.000	11	61.1
160744799	2228.571	424.000	665.866	0.001	0.432	1.007	1.000	392.960	0.000	7	50.0
160825720	1913.692	626.731	995.323	0.001	0.406	1.000	1.000	22.236	0.004	3	11.5
160866180	1946.065	442.129	693.983	0.001	0.417	1.007	1.000	49.679	0.000	11	35.5
160886410	2064.643	560.786	862.252	0.002	0.400	1.003	1.000	52.059	0.000	4	28.6
160906640	2423.923	693.000	1135.187	0.000	0.383	1.011	1.000	121.390	0.000	6	46.2
160947100	2075.308	683.423	1185.677	0.001	0.385	1.003	1.000	22.816	0.004	5	19.2
160967330	1987.429	634.929	966.519	0.001	0.408	1.002	1.000	9.885	0.195	2	14.3
160987560	1719.786	639.071	1201.369	0.001	0.383	0.983	1.000	31.118	0.000	3	21.4
161007791	2357.867	544.133	811.056	0.001	0.452	0.992	1.000	27.899	0.002	3	20.0
161270782	1797.643	593.929	1012.992	0.001	0.384	1.004	1.000	35.286	0.000	3	21.4
161311242	1719.167	604.667	1075.992	0.001	0.368	1.009	0.999	63.910	0.000	3	25.0
161331472	1695.000	518.000	819.946	0.001	0.428	0.995	0.999	14.964	0.036	2	50.0
161351702	1445.000	587.667	939.129	0.001	0.399	0.988	1.000	7.266	0.297	0	0.0
161412393	3124.375	780.500	1293.935	0.000	0.372	1.023	0.998	211.560	0.000	5	62.5
161473083	2550.353	490.824	730.123	0.000	0.430	1.021	0.999	100.668	0.000	8	47.1
161554003	2834.154	591.308	788.272	0.000	0.430	1.014	0.999	98.348	0.000	7	53.8
178713055	4636.917	677.000	763.204	0.002	0.440	1.008	1.000	12.229	0.347	4	33.3
184349034	4416.250	793.625	1019.770	0.000	0.434	1.020	0.999	118.340	0.000	5	62.5
206906765	2840.000	697.000	988.841	0.000	0.402	1.025	0.997	28.585	0.000	1	100.0
208027353	4232.471	745.765	881.398	0.000	0.466	1.018	0.999	79.734	0.000	6	35.3
246515023	1653.158	447.789	898.069	0.001	0.393	0.994	1.000	1091.427	0.000	9	47.4
250593475	3409.000	672.000	985.132	0.000	0.395	1.025	0.998	35.450	0.000	1	100.0
256789458	3791.333	637.667	895.521	0.001	0.407	1.027	0.999	194.250	0.000	7	77.8
275382046	3850.857	557.286	706.930	0.000	0.451	1.014	0.999	692.460	0.000	4	57.1
289996019	8115.870	803.478	786.318	0.000	0.503	1.010	0.999	29040.171	0.000	9	39.1
295137534	2108.833	478.833	682.648	0.001	0.416	1.005	1.000	15.348	0.082	3	25.0

336497421	3097.692	736.385	1015.711	0.000	0.424	1.009	1.000	36.235	0.000	5	38.5
338793263	6271.500	567.500	826.587	0.001	0.447	1.018	0.998	125.722	0.000	3	75.0
355657046	4640.000	852.000	1037.468	0.000	0.447	1.008	1.000	22.642	0.007	2	50.0
368533040	2045.313	580.938	928.606	0.001	0.383	1.002	1.000	55.760	0.000	5	31.3
370027359	4432.733	752.067	874.115	0.000	0.461	1.010	1.000	59.517	0.000	8	53.3
370425937	3227.286	582.714	797.619	0.000	0.464	1.006	0.999	86.874	0.000	9	42.9
375450439	4215.833	733.933	885.330	0.001	0.460	1.013	0.999	92.768	0.000	8	26.7
404239096	3714.462	800.231	996.966	0.000	0.452	1.006	1.000	30.751	0.000	5	38.5
414519462	3854.316	659.789	898.066	0.000	0.444	1.029	0.999	275.015	0.000	12	63.2
451588811	4490.462	750.154	1021.480	0.000	0.419	1.017	0.999	176.918	0.000	8	61.5
465578759	5857.389	605.222	697.232	0.001	0.462	1.029	0.999	101.431	0.000	9	50.0
492786515	3335.500	617.500	795.361	0.000	0.436	1.013	0.999	102.583	0.000	5	35.7
508703490	3972.538	554.923	636.508	0.000	0.486	1.015	0.998	68.812	0.000	5	38.5
514014184	2001.000	486.000	649.836	0.001	0.425	1.020	0.999	9.763	0.202	1	50.0
516889361	5451.143	738.571	952.497	0.001	0.415	1.011	0.999	22.499	0.013	5	71.4
517810313	5185.200	800.800	913.991	0.000	0.484	1.008	0.999	146.926	0.000	6	40.0
533247696	3876.000	967.000	1364.634	0.000	0.408	1.014	0.999	12.955	0.073	0	0.0
550534656	4023.200	623.400	945.900	0.001	0.395	1.028	0.998	301.929	0.000	3	60.0
553359145	4925.235	659.706	777.631	0.000	0.498	1.005	0.999	111.415	0.000	8	47.1
561079058	4802.250	311.000	421.391	0.000	0.440	1.038	0.997	289.355	0.000	4	100.0
604812005	1080.286	476.071	1042.635	0.001	0.317	1.005	1.000	57.001	0.000	5	35.7
612472597	6825.500	844.722	929.075	0.000	0.465	1.014	0.999	142.129	0.000	10	55.6
638754422	2869.438	599.688	916.713	0.000	0.416	1.014	0.999	85.433	0.000	8	50.0
643185023	4000.909	546.909	652.042	0.001	0.421	1.058	0.999	48.358	0.000	9	81.8
650853796	3500.571	620.000	938.962	0.000	0.434	1.005	1.000	246.565	0.000	5	35.7
658594300	3026.143	509.429	621.052	0.004	0.456	0.990	1.000	92.815	0.000	2	28.6
663835652	4676.214	735.929	875.953	0.000	0.473	1.012	0.999	67.510	0.000	7	50.0
668248235	5411.933	886.133	1008.437	0.000	0.470	1.007	1.000	72.393	0.000	5	33.3
675950834	4844.500	776.500	884.774	0.001	0.429	1.043	0.999	14.054	0.120	2	50.0
682102541	6973.200	682.200	794.991	0.000	0.440	1.027	0.998	471.549	0.000	5	100.0
682449369	4265.765	732.353	789.874	0.000	0.488	1.008	1.000	49.628	0.000	7	41.2
686765762	2499.167	604.833	928.135	0.001	0.399	1.008	1.000	96.172	0.000	11	36.7
706846339	8707.000	841.600	836.087	0.000	0.554	0.989	0.999	29.038	0.002	1	20.0
737052003	8367.188	809.250	737.975	0.000	0.535	1.005	1.000	26.091	0.010	3	18.8
739574095	7912.000	454.000	572.270	0.000	0.441	1.039	0.998	177.502	0.000	5	83.3
763395383	5261.500	935.786	1082.968	0.000	0.470	1.007	0.999	53.381	0.000	6	42.9
763435843	7986.769	1042.231	1080.981	0.000	0.485	1.015	0.999	3260.031	0.000	3	23.1
763456073	5010.500	705.563	699.509	0.000	0.532	0.995	0.999	40.817	0.000	3	18.8
763476303	4652.786	606.000	712.427	0.000	0.455	1.020	0.998	465.340	0.000	8	57.1
763516763	4632.400	682.200	774.807	0.000	0.467	1.019	0.998	594.451	0.000	14	56.0
763536994	7445.205	896.923	929.053	0.000	0.472	1.018	0.999	251.250	0.000	25	64.1
763557224	4849.231	487.462	476.215	0.001	0.476	1.020	0.999	229.050	0.000	7	53.8
763597684	12569.040	1478.440	1393.451	0.000	0.514	1.010	0.999	215.486	0.000	13	52.0
763638144	3641.607	622.143	769.524	0.001	0.465	1.012	1.000	59.580	0.000	12	42.9
763678604	8816.714	751.286	856.269	0.000	0.447	1.039	0.994	400.068	0.000	7	100.0
763698834	12800.516	1107.194	1013.462	0.000	0.521	1.012	0.999	169.263	0.000	21	67.7
763719065	3045.387	502.839	550.767	0.000	0.475	1.012	0.999	231.702	0.000	14	45.2
763759525	6073.839	706.742	742.761	0.000	0.489	1.014	0.999	125.817	0.000	14	45.2
763820215	4781.759	501.724	559.172	0.000	0.498	1.010	0.999	123.180	0.000	12	41.4
763840445	5994.529	748.765	767.210	0.000	0.526	1.005	0.999	47.552	0.000	15	44.1
763860675	5276.370	815.593	852.824	0.000	0.484	1.006	0.999	211.552	0.000	14	51.9
763880905	7309.118	799.824	900.586	0.000	0.462	1.033	0.997	472.715	0.000	11	64.7
763901136	4079.594	608.781	763.418	0.000	0.456	1.016	0.999	219.054	0.000	18	56.3
763921366	5632.389	743.333	800.360	0.000	0.487	1.015	0.999	128.247	0.000	9	50.0
763982056	6359.393	834.750	882.231	0.000	0.484	1.012	0.999	217.423	0.000	12	42.9
764002286	7840.444	914.926	986.932	0.000	0.468	1.024	0.999	552.738	0.000	16	59.3
764042746	3932.429	540.857	634.300	0.000	0.483	1.010	0.999	155.255	0.000	10	35.7
764062976	5557.483	663.483	643.994	0.000	0.532	1.009	0.999	100.916	0.000	11	37.9
764083206	8026.586	1044.966	1089.168	0.000	0.478	1.025	0.999	346.887	0.000	18	62.1
764143897	6159.765	782.000	785.448	0.000	0.502	1.007	1.000	51.564	0.000	11	32.4
764184357	4275.242	676.879	806.854	0.000	0.466	1.014	0.999	110.978	0.000	13	39.4
764224817	5152.143	817.000	894.336	0.000	0.476	1.008	0.998	93.120	0.000	12	42.9
764245047	5976.667	514.400	463.626	0.000	0.497	1.032	0.995	525.173	0.000	10	66.7
764285508	5815.800	879.500	835.008	0.000	0.514	1.011	0.998	57.707	0.000	5	50.0
764305738	13510.724	1194.793	1271.966	0.000	0.466	1.012	0.999	108.256	0.000	14	48.3

764325968	6048.680	932.920	959.510	0.000	0.488	1.007	0.999	61.084	0.000	11	44.0
764346198	3982.448	584.793	679.340	0.000	0.471	1.009	1.000	309.012	0.000	15	51.7
764366428	4487.800	476.067	533.164	0.001	0.442	1.047	0.998	426.162	0.000	11	73.3
764447348	4096.269	590.962	634.328	0.001	0.475	1.009	0.999	85.207	0.000	12	46.2
764467579	4336.400	644.320	767.960	0.000	0.465	1.015	1.000	218.667	0.000	13	52.0
764487809	5337.037	755.926	826.091	0.000	0.466	1.012	1.000	51.463	0.000	12	44.4
764508039	6627.929	717.643	815.001	0.000	0.457	1.015	0.999	52.692	0.000	5	35.7
764588959	3944.438	536.188	572.497	0.001	0.482	1.012	0.998	32.146	0.000	6	37.5
764649650	3984.500	649.056	775.981	0.000	0.453	1.015	0.999	103.596	0.000	11	61.1
764669880	4086.786	802.607	1006.137	0.000	0.437	1.013	1.000	153.600	0.000	16	57.1
764710340	3035.500	222.625	216.167	0.001	0.528	1.012	0.995	26.854	0.003	5	62.5
764750800	5174.286	973.500	1233.578	0.000	0.435	1.010	0.999	144.569	0.000	6	42.9
764811490	6769.765	801.882	896.951	0.000	0.491	1.009	0.999	41.643	0.000	7	41.2
764831721	4451.462	879.000	1110.755	0.000	0.435	1.017	0.999	97.348	0.000	5	38.5
764872181	4615.786	758.429	900.532	0.000	0.460	1.014	0.999	675.609	0.000	4	28.6
764892411	3396.200	751.333	922.326	0.000	0.441	1.009	0.999	42.748	0.000	6	40.0
764953101	4569.000	889.333	1132.786	0.000	0.435	1.017	0.999	116.747	0.000	6	50.0
765013792	3584.294	621.294	771.243	0.000	0.449	1.016	1.000	46.205	0.000	6	35.3
765034022	3548.294	610.647	741.519	0.000	0.445	1.024	0.999	73.042	0.000	8	47.1
765074482	3701.500	721.636	848.931	0.000	0.454	1.008	1.000	41.406	0.000	9	40.9
765094712	4350.500	806.917	1040.242	0.000	0.434	1.019	0.998	204.388	0.000	14	58.3
765135172	2171.333	470.667	749.722	0.001	0.366	1.032	0.997	57.260	0.000	3	100.0
765155402	3940.500	705.750	800.231	0.000	0.470	1.012	0.999	43.356	0.000	7	43.8
765195863	4010.944	649.833	765.138	0.000	0.458	1.022	0.999	169.609	0.000	8	44.4
765216093	4528.267	779.400	850.444	0.000	0.480	1.007	1.000	43.412	0.000	6	40.0
765256553	5034.429	748.357	809.709	0.000	0.476	1.021	0.999	74.962	0.000	7	50.0
765276783	4353.867	722.000	879.145	0.000	0.445	1.019	0.999	151.552	0.000	7	46.7
765317243	4394.143	562.929	636.450	0.000	0.479	1.012	1.000	65.533	0.000	5	35.7
765337473	3898.722	668.889	812.135	0.000	0.471	1.013	0.999	101.182	0.000	9	50.0
765377934	4966.900	895.900	1144.060	0.000	0.426	1.026	0.997	188.363	0.000	7	70.0
765398164	5593.500	526.000	456.615	0.000	0.539	1.008	0.999	9.215	0.418	0	0.0
765519544	5965.750	933.500	1164.142	0.000	0.433	1.022	0.998	86.139	0.000	4	100.0
765539774	3651.667	526.667	688.829	0.000	0.421	1.040	0.993	130.102	0.000	3	100.0
765560005	5027.375	954.625	1182.247	0.000	0.436	1.016	0.999	87.492	0.000	10	62.5
765620695	4423.500	347.500	445.176	0.000	0.408	1.070	0.989	243.669	0.000	2	100.0
765640925	7139.000	1043.500	1327.182	0.000	0.434	1.012	1.000	49.039	0.000	2	100.0
765661155	6054.000	641.500	897.076	0.000	0.404	1.029	0.999	145.822	0.000	2	100.0
765701615	8096.250	1018.250	1194.786	0.000	0.447	1.016	1.000	51.824	0.000	3	75.0
809635352	5138.056	730.833	928.697	0.000	0.466	1.010	0.999	58.642	0.000	7	38.9
823052294	2376.750	554.750	912.080	0.004	0.305	1.017	0.998	24.820	0.003	2	50.0
857980665	5607.250	723.000	950.056	0.002	0.399	1.031	0.995	392.327	0.000	2	50.0
861967750	3600.000	654.500	862.361	0.000	0.419	1.029	0.998	103.588	0.000	2	50.0
863126187	7000.154	932.615	1105.728	0.000	0.448	1.008	0.999	118.396	0.000	8	61.5
875002022	3632.000	353.500	452.360	0.000	0.453	1.037	0.997	41.479	0.000	3	75.0
892969023	1878.176	498.235	805.060	0.001	0.409	0.994	1.000	97.975	0.000	4	23.5
901775393	5027.000	558.000	517.766	0.000	0.549	0.996	0.997	15.082	0.058	2	100.0
937495960	2651.583	598.333	831.509	0.000	0.421	1.010	1.000	24.178	0.004	3	25.0
938202701	11850.000	802.000	999.507	0.000	0.426	1.042	0.995	163.100	0.000	1	100.0
953045535	4506.063	609.250	675.959	0.001	0.466	1.022	0.998	156.820	0.000	10	62.5
964271349	228.000	35.000	116.801	0.006	0.170	1.284	0.952	1462.212	0.000	1	100.0
970836795	3266.250	659.000	989.281	0.000	0.425	1.012	0.999	84.582	0.000	9	56.3
Passing Rate %									9(5.2%)	8	51.0

**J*: the average number of individuals per niche (local community) in each metacommunity, *S*: the average species numbers per niche (local community) in each metacommunity, θ: the average fundamental biodiversity parameter per niche (local community) in each metacommunity, *m*: the average of the migration coefficients, *x*: the average of the birth to death ratio, γ: the average of the migration rate, R^2: the goodness-of-fitting index, χ^2-value: the χ^2-value of chi-squared test for observed value against predicted value, *p*-value for the χ^2-test; when *p*-value > 0.05, the metacommunity satisfies the NNH model. The last two columns are the number and percentage of local communities (niches) that passed the local neutrality test. Note that $R^2=1$ resulted from approximation with four effective digits only (*e.g.*, 0.99995, exact 1 is nearly impossible to achieve). See Fig 3 for an example of fitting the NNH model.

Table S13. Test results of fitting NNH (multi-site neutral) model to Gut datasets (*Sites*=7, *Subjects*=11)

Group ID	J	S	θ	m	x	γ	R^2	χ^2	p-value	N_{pass}	$\%_{(pass)}$
S400	3630.571	583.143	557.865	0.000	0.513	0.998	1.000	6.383	0.701	0	0.0
S401	3863.857	686.429	602.807	0.000	0.538	0.988	0.995	45.244	0.000	2	28.6
S402	3873.000	716.714	674.731	0.000	0.521	0.994	0.999	22.312	0.004	0	0.0
S403	4190.143	827.429	837.208	0.000	0.499	0.993	0.999	11.252	0.188	1	14.3
S404	4276.286	672.571	632.176	0.000	0.519	0.999	1.000	5.554	0.697	0	0.0
S405	4462.429	780.000	781.444	0.000	0.498	0.997	1.000	11.017	0.275	1	14.3
S406	5326.429	636.286	593.949	0.000	0.513	1.006	1.000	19.805	0.031	4	57.1
S407	1223.000	332.286	299.728	0.001	0.543	0.967	0.993	38.469	0.000	3	42.9
S408	3776.571	741.286	828.736	0.000	0.470	1.009	0.999	45.457	0.000	2	28.6
S409	1807.714	529.143	643.545	0.001	0.474	0.976	0.996	59.115	0.000	3	42.9
S410	1803.143	634.571	748.939	0.001	0.473	0.971	0.997	46.542	0.000	3	42.9
Passing Rate									4(36.4%)	2	24.7

**J*: the average number of individuals per niche (local community) in each metacommunity, *S*: the average species numbers per niche (local community) in each metacommunity, θ: the average fundamental biodiversity parameter per niche (local community) in each metacommunity, *m*: the average of the migration coefficients, *x*: the average of the birth to death ratio, γ: the average of the migration rate, R^2: the goodness-of-fitting index, χ^2-value: the χ^2-value of chi-squared test for observed value against predicted value, *p*-value for the χ^2-test; when *p*-value > 0.05, the metacommunity satisfies the NNH model. The last two columns are the number and percentage of local communities (niches) that passed the local neutrality test. Note that $R^2=1$ resulted from approximation with four effective digits only (*e.g.*, 0.99995, exact 1 is nearly impossible to achieve). See Fig 3 for an example of fitting the NNH model.

Table S14. Test results of fitting NNH (multi-site neutral) model to Lung datasets (*Sites*=4, *Subjects*=139)

Group ID	J	S	θ	m	x	γ	R^2	χ^2	p-value	N_{pass}	$\%_{(pass)}$
A1	1666	27.000	9.935	0.000	0.856	0.738	0.959	5.077	0.828	0	0.0
A2	1666	34.667	21.489	0.001	0.668	0.957	0.949	14.136	0.118	1	33.3
A3	1666	51.000	31.833	0.001	0.653	0.966	0.989	22.149	0.014	1	25.0
A4	1666	33.250	20.395	0.001	0.610	0.979	0.957	91.916	0.000	3	75.0
A5	1666	44.750	30.621	0.001	0.562	1.089	0.922	85.178	0.000	3	75.0
A6	1666	47.000	31.144	0.001	0.681	0.916	0.988	7.975	0.631	1	33.3
A7	1666	54.500	36.638	0.001	0.590	1.027	0.991	32.719	0.000	2	50.0
A8	1666	77.250	43.711	0.001	0.642	1.002	0.985	9.408	0.400	1	25.0
A9	1666	51.500	29.486	0.001	0.643	0.983	0.960	829022.97	0.000	1	25.0
A10	1666	66.500	37.464	0.001	0.639	0.984	0.972	24.824	0.006	1	25.0
A11	1666	24.000	15.072	0.001	0.628	0.973	0.950	10.769	0.096	0	0.0
A12	1666	50.750	24.575	0.001	0.692	0.961	0.969	14.465	0.153	0	0.0
A13	1666	45.500	33.243	0.001	0.615	0.969	0.972	63.277	0.000	3	75.0
A14	1666	62.500	26.636	0.001	0.719	0.938	0.972	5.673	0.578	1	25.0
A15	1666	55.667	26.785	0.001	0.693	0.971	0.940	11.467	0.322	0	0.0
A16	1666	43.333	23.533	0.001	0.648	0.996	0.964	37.162	0.000	2	66.7
A17	1666	61.000	37.740	0.001	0.651	0.990	0.978	17.242	0.045	0	0.0
A18	1666	60.250	31.847	0.001	0.660	1.005	0.986	6.302	0.709	0	0.0
A19	1666	59.750	34.856	0.001	0.598	1.018	0.989	890.732	0.000	1	25.0
A20	1666	83.500	48.477	0.001	0.642	0.994	0.987	10.829	0.288	1	25.0
A21	1666	28.000	22.134	0.001	0.648	0.924	0.983	35.293	0.000	1	25.0
A22	1666	47.333	31.301	0.001	0.532	1.172	0.885	113.656	0.000	3	100.0
A23	1666	57.750	31.832	0.001	0.670	0.967	0.981	9.803	0.458	0	0.0
A24	1666	36.750	25.710	0.001	0.622	0.982	0.987	21.052	0.007	2	50.0
A25	1666	33.500	23.384	0.001	0.649	0.987	0.973	66.063	0.000	1	50.0
A26	1666	56.250	37.154	0.001	0.566	1.089	0.947	58.184	0.000	3	75.0
A27	1666	51.250	27.587	0.001	0.676	0.983	0.980	12.315	0.264	1	25.0
A28	1666	95.250	57.935	0.001	0.609	0.983	0.991	1107.891	0.000	1	25.0
A29	1666	51.000	29.063	0.001	0.647	0.996	0.881	50.015	0.000	3	100.0
A30	1666	68.000	37.347	0.001	0.668	0.961	0.992	9.072	0.525	0	0.0
A31	1666	26.750	9.490	0.000	0.854	0.754	0.952	3.918	0.951	0	0.0
A32	1666	73.250	34.847	0.001	0.671	0.939	0.944	181.826	0.000	2	50.0
A33	1666	46.500	18.141	0.001	0.761	0.919	0.955	8.343	0.595	1	25.0
A35	1666	51.500	42.502	0.001	0.529	1.072	0.989	27.275	0.001	2	50.0
A36	1666	20.000	1.267	0.001	0.731	2.225	0.152	3.379	0.908	0	0.0
A37	1666	18.000	5.170	0.001	0.802	1.330	0.980	1.019	0.999	0	0.0
A38	1666	37.000	28.160	0.001	0.560	1.084	0.944	152.210	0.000	2	66.7
A39	1666	30.333	17.865	0.001	0.605	1.069	0.927	76.851	0.000	2	66.7
A40	1666	45.667	19.334	0.001	0.724	0.957	0.974	5.099	0.826	0	0.0
A41	1666	44.000	15.745	0.001	0.814	0.843	0.941	4.227	0.836	0	0.0
A42	1666	36.333	18.305	0.001	0.635	1.072	0.864	53.877	0.000	2	66.7
A43	1666	29.667	20.877	0.001	0.623	0.984	0.922	46.201	0.000	2	66.7
A44	1666	86.667	42.125	0.001	0.681	0.971	0.979	8.379	0.496	0	0.0
A45	1666	63.000	37.667	0.001	0.610	0.998	0.990	7.424	0.593	1	33.3
A46	1666	30.667	15.907	0.001	0.728	0.870	0.929	10.427	0.236	0	0.0
A47	1666	17.500	17.755	0.001	0.447	1.113	0.958	636.512	0.000	2	100.0
A48	1666	24.667	29.853	0.001	0.454	1.057	0.968	455.113	0.000	3	100.0

A49	1666	56.667	34.603	0.001	0.631	0.967	0.949	40.569	0.000	1	33.3
A50	1666	87.000	61.353	0.001	0.567	1.042	0.980	18.530	0.018	1	50.0
A51	1666	39.000	22.633	0.001	0.710	0.880	0.926	37.667	0.000	2	100.0
A52	1666	54.333	24.848	0.001	0.695	0.981	0.962	7.221	0.614	0	0.0
A53	1666	38.000	36.513	0.001	0.451	1.149	0.982	563.505	0.000	3	100.0
A54	1666	56.000	24.778	0.001	0.703	0.981	0.944	7.082	0.629	0	0.0
A55	1666	32.000	18.439	0.001	0.646	0.974	0.915	164.501	0.000	1	50.0
A56	1666	100.667	50.200	0.001	0.702	0.947	0.948	9.905	0.358	0	0.0
A57	1666	53.500	33.437	0.001	0.667	0.946	0.918	14.910	0.093	0	0.0
A58	1666	35.000	13.257	0.001	0.805	0.859	0.916	3.954	0.914	0	0.0
A59	1666	57.000	39.550	0.001	0.555	1.002	0.983	116.453	0.000	1	50.0
A60	1666	50.667	28.901	0.001	0.658	0.988	0.979	8.870	0.545	0	0.0
A61	1666	36.000	30.354	0.001	0.487	1.092	0.991	147.289	0.000	2	100.0
A62	1666	24.333	22.864	0.001	0.558	1.059	0.970	5761.735	0.000	2	66.7
A63	1666	41.000	28.547	0.001	0.677	0.969	0.946	13.408	0.202	1	33.3
A64	1666	52.667	25.209	0.001	0.694	0.970	0.945	15.587	0.076	0	0.0
A65	1666	88.333	47.019	0.001	0.658	0.986	0.983	5.761	0.764	0	0.0
A66	1666	61.333	31.859	0.001	0.669	0.978	0.975	11.272	0.258	0	0.0
A67	1666	102.000	56.597	0.001	0.638	1.004	0.997	2.785	0.947	1	33.3
A68	1666	30.333	11.830	0.000	0.813	0.818	0.944	4.239	0.936	0	0.0
A70	1666	61.333	51.623	0.001	0.511	1.085	0.981	38.502	0.000	2	66.7
A71	1666	69.667	59.087	0.001	0.532	1.031	0.966	38.605	0.000	2	66.7
A72	1666	13.333	22.616	0.001	0.271	1.349	0.930	95625.340	0.000	3	100.0
A73	1666	44.000	21.943	0.001	0.725	0.915	0.978	4.128	0.903	0	0.0
A75	1666	33.667	20.721	0.001	0.652	0.990	0.968	21.986	0.015	1	33.3
A77	1666	55.667	46.325	0.001	0.506	1.149	0.959	58.241	0.000	2	66.7
A78	1666	38.500	16.376	0.001	0.737	0.945	0.928	6.567	0.682	0	0.0
A79	1666	52.000	31.743	0.001	0.582	1.082	0.864	27.517	0.001	1	100.0
A80	1666	40.000	17.565	0.001	0.747	0.911	0.958	8.371	0.593	0	0.0
A81	1666	41.000	30.202	0.001	0.568	1.089	0.984	17.346	0.067	1	33.3
A82	1666	44.000	27.460	0.001	0.652	0.922	0.960	10.037	0.262	1	33.3
A83	1666	57.000	37.156	0.001	0.613	1.002	0.961	11.365	0.252	2	66.7
A84	1666	70.333	53.917	0.001	0.567	1.058	0.975	120.804	0.000	1	33.3
A85	1666	37.333	18.353	0.001	0.734	0.929	0.985	6.765	0.747	0	0.0
A86	1666	25.500	12.265	0.001	0.730	0.903	0.957	6.193	0.720	0	0.0
A87	1666	54.333	22.795	0.001	0.725	0.953	0.914	17.843	0.037	0	0.0
A88	1666	45.000	43.593	0.001	0.481	1.085	0.984	136.317	0.000	2	100.0
A89	1666	35.000	17.217	0.001	0.733	0.898	0.947	6.498	0.772	0	0.0
A90	1666	70.667	35.368	0.001	0.689	0.979	0.972	7.258	0.610	0	0.0
A91	1666	50.000	27.722	0.000	0.796	0.816	0.977	3.003	0.981	0	0.0
A92	1666	58.000	26.589	0.001	0.736	0.923	0.941	8.129	0.521	0	0.0
A93	1666	44.667	23.060	0.001	0.636	1.056	0.885	42.580	0.000	2	66.7
A94	1666	38.667	16.571	0.001	0.746	0.911	0.959	5.780	0.833	0	0.0
A95	1666	26.500	136.961	0.001	0.328	1.153	0.984	19449370.	0.000	2	100.0
A96	1666	53.000	34.189	0.001	0.589	1.013	0.970	1259.071	0.000	1	33.3
A97	1666	67.000	32.733	0.001	0.709	0.946	0.982	3.055	0.962	0	0.0
A98	1666	11.000	0.575	0.001	0.732	2.472	0.570	0.510	0.998	0	0.0
A99	1666	24.000	11.465	0.001	0.761	0.849	0.884	11.850	0.158	1	50.0
A100	1666	40.500	23.440	0.001	0.645	0.980	0.984	12.890	0.168	0	0.0
A101	1666	39.000	33.481	0.001	0.480	1.159	0.927	155.152	0.000	2	100.0
A102	1666	44.333	18.727	0.001	0.797	0.849	0.957	4.207	0.938	0	0.0

A103	1666	51.333	37.302	0.001	0.621	0.958	0.979	25.658	0.002	1	33.3
A104	1666	17.000	1.523	0.001	0.687	2.254	0.244	5.851	0.557	0	0.0
A105	1666	75.667	41.422	0.001	0.638	1.010	0.996	4.237	0.895	1	33.3
A106	1666	26.000	8.600	0.001	0.796	0.901	0.889	4.865	0.900	0	0.0
A107	1666	76.667	30.600	0.001	0.755	0.929	0.983	4.315	0.890	0	0.0
A108	1666	35.667	13.688	0.001	0.748	0.943	0.987	3.040	0.963	0	0.0
A109	1666	35.667	17.590	0.001	0.695	0.984	0.913	11.780	0.226	0	0.0
A110	1666	8.000	7.431	0.000	0.738	0.670	0.924	6.716	0.082	0	0.0
A111	1666	29.000	18.487	0.001	0.593	1.053	0.928	42.357	0.000	2	100.0
R01	1666	34.000	25.904	0.001	0.634	0.919	0.958	14.500	0.106	0	0.0
R02	1666	65.333	40.360	0.001	0.626	0.994	0.959	29.608	0.001	1	33.3
R03	1666	33.750	30.789	0.001	0.518	1.033	0.992	25.306	0.005	3	75.0
R04	1666	53.500	34.446	0.001	0.603	1.036	0.989	21.124	0.020	1	25.0
R05	1666	18.667	5.908	0.001	0.784	1.126	0.858	5.643	0.775	1	33.3
R06	1666	12.500	15.294	0.001	0.392	1.173	0.830	2122.417	0.000	2	100.0
R07	1666	34.000	18.398	0.001	0.693	0.947	0.982	5.311	0.869	1	33.3
R08	1666	32.250	22.132	0.001	0.603	0.980	0.981	36.328	0.000	1	25.0
R09	1666	60.000	27.958	0.001	0.689	0.988	0.937	12.134	0.206	0	0.0
R10	1666	49.000	48.358	0.001	0.469	1.106	0.972	235.878	0.000	3	75.0
R11	1666	95.000	52.945	0.001	0.646	0.997	0.990	6.364	0.607	1	25.0
R12	1666	58.333	34.680	0.001	0.631	1.015	0.960	16.610	0.055	1	33.3
R13	1666	22.000	12.950	0.001	0.604	1.693	0.971	6.557	0.476	1	33.3
R14	1666	58.000	30.183	0.000	0.766	0.788	0.832	17.668	0.039	0	0.0
R15	1666	67.750	40.194	0.001	0.626	1.019	0.972	9.928	0.356	2	50.0
R16	1666	38.667	21.369	0.001	0.690	0.942	0.956	8.901	0.446	0	0.0
R17	1666	51.750	30.541	0.001	0.640	1.036	0.975	472.964	0.000	2	50.0
R18	1666	65.250	34.091	0.001	0.651	1.010	0.989	8.815	0.454	1	25.0
R19	1666	62.750	44.632	0.001	0.557	1.054	0.974	68.481	0.000	2	50.0
R20	1666	31.667	23.630	0.001	0.551	1.003	0.970	38.187	0.000	2	66.7
R22	1666	50.000	28.040	0.001	0.671	0.957	0.989	14.001	0.173	1	25.0
R24	1666	53.500	32.307	0.001	0.623	1.015	0.971	42.024	0.000	1	25.0
R25	1666	99.750	55.661	0.001	0.667	0.973	0.988	4.392	0.884	0	0.0
R26	1666	39.500	61.892	0.001	0.371	1.121	0.994	3874889.0	0.000	3	75.0
R27	1666	59.750	39.194	0.001	0.675	0.931	0.995	10.183	0.425	1	25.0
R28	1666	36.500	15.130	0.001	0.684	1.315	0.927	54.675	0.000	1	25.0
R29	1666	65.750	42.636	0.001	0.602	1.012	0.980	23.293	0.003	0	0.0
R30	1666	60.667	39.076	0.001	0.593	1.040	0.987	19.741	0.020	1	33.3
Passing Rate									72 (53.3%)	1	32.6

*J: the average number of individuals per niche (local community) in each metacommunity, S: the average species numbers per niche (local community) in each metacommunity, θ: the average fundamental biodiversity parameter per niche (local community) in each metacommunity, m: the average of the migration coefficients, x: the average of the birth to death ratio, $\boldsymbol{\gamma}$: the average of the migration rate, R^2: the goodness-of-fitting index, χ^2-value: the χ^2-value of chi-squared test for observed value against predicted value, p-value for the χ^2-test; when p-value > 0.05, the metacommunity satisfies the NNH model. The last two columns are the number and percentage of local communities (niches) that passed the local neutrality test. Note that $R^2=1$ resulted from approximation with four effective digits only (*e.g.*, 0.99995, exact 1 is nearly impossible to achieve). See Fig 3 for an example of fitting the NNH model.
